# Systematic analysis between inflammation-related index and sex hormones in American adults: cross-sectional research based NHANES 2013-2016

**DOI:** 10.3389/fimmu.2023.1175764

**Published:** 2023-05-26

**Authors:** Chengcheng Wei, Wenting Zhang, Jiabi Chen, Qingliu He, Li Cao, Pu Zhang, Changqi Deng, Ming Xiong, Yu Huang, Haixin Guo, Miao Wang, Zhaohui Chen

**Affiliations:** ^1^ Department of Urology, Union Hospital, Tongji Medical College, Huazhong University of Science and Technology, Wuhan, Hubei, China; ^2^ Department of Obstetrics and Gynecology, Union Hospital, Tongji Medical College, Huazhong University of Science and Technology, Wuhan, Hubei, China; ^3^ Department of Urology, The Second Affiliated Hospital of Fujian Medical University, Quanzhou, China; ^4^ Department of Orthopaedic, Union Hospital, Tongji Medical College, Huazhong University of Science and Technology, Wuhan, Hubei, China; ^5^ Department of Ultrasound, the Second Affiliated Hospital of Fujian Medical University, Quanzhou, China

**Keywords:** inflammation-related index, sex hormones, systemic immune-inflammation index, machine learning, National Health and Nutrition Examination Survey (NHANES)

## Abstract

**Background:**

A series of novel inflammation-related indexes has been confirmed to be efficient indicators of human immune and inflammatory status, with great potential as predictors for a variety of diseases. However, the association between inflammation-related indexes and sex hormones in the general population remained uncertain.

**Methods:**

We incorporated data from the NHANES 2013-2016 survey of American adults. On the basis of distribution and comparison analysis, we chose to undertake separate analyses of men and women (including premenopausal and postmenopausal groups). Multivariable weighted linear regression models, eXtreme Gradient Boosting (XGBoost) models, generalized linear analysis, stratified models, logistic regression models and sensitivity analysis were utilized to assess the relationships between inflammation-related indexes and sex hormones.

**Results:**

Total 9372 participants out of 20146 were fitted into our research. We conducted separate gender analysis due to different distribution. Multivariable weighted linear regression indicated every component of the inflammation-related index was negatively correlated with at least one component of the male hormone indexes. However, SII, NLR, PPN, and NC were associated positively with female estradiol. XGBoost identify SII, PLR and NLR were the critical indexes on sex hormones. Inflammation-related indexes was associated with Testosterone deficiency in male and postmenstrual group and associated with Excessive Estradiol in premenstrual group. Finally, the subgroup analysis revealed that the association between sex hormones and inflammatory indicators was prominent in American adults over the age of 60 or those with BMI (>28 kg/m^2^).

**Conclusion:**

In all, inflammation-related indexes act as independent risks associated with sex hormone alterations and metabolic disorder in both genders. Using multiple models, we revealed the relative importance of inflammation-related indexes. Subgroup analysis also identified the high-risk population. More prospective and experimental research should be conducted to validate the results.

## 1 Introduction

A series of novel inflammation-related indexes, derived from neutrophil (NC), platelet (PLT), and lymphocyte (LC) counts, such as the systemic immune-inflammation index (SII), platelet-to-lymphocyte ratio (PLR), neutrophil-to-lymphocyte ratio (NLR), and the product of platelet count and neutrophil count (PPN), have been discovered to be able to evaluate the systemic immune and inflammatory status of the human body. These indicators, for example, are elevated in patients with inflammatory diseases and are associated with disease activity (1, 2). Additionally, these markers may potentially suggest a poor prognosis for cardiovascular and cerebrovascular illnesses (3, 4). It was also widely recognized that high Systemic immune-inflammation index (SII) levels typically indicate shorter overall survival (OS) in patients with various malignancies, including colorectal cancer, lung cancer, pancreatic cancer, gynecological and breast cancers (5–8). While in general populations, these indexes are positively correlated with increased cancer incidence and all-cause mortality (9–12).

A few prior epidemiological studies have investigated the association between the inflammation-related indexes and reproductive health. For instance, leukocytosis was recognized to bridge low-grade chronic inflammation and polycystic ovary syndrome (PCOS) which presented with hyperandrogenism, and NLR level seemed to be a potential effective predictor among several parameters (13, 14). Moreover, patients with erectile dysfunction (ED), which might result from sex hormonal disorders, had significant higher NLR and PLR than healthy subjects (15, 16). It has been reported that positive correlation existed between NLR and presence and progression of benign prostatic hyperplasia (BPH), which correlated with testosterone levels as well (17–19). Additionally, research have shown that an increase in pro-inflammatory cytokines was associated with sex hormone disorders (20, 21).

Sex hormones are of significance for the proper functioning of the body. Total testosterone (TT) and estradiol (E_2_) are the two primary sex hormones in humans. SHBGs are a kind of transporter protein found in plasma that binds and transports testosterone and estradiol, hence controlling the amount of biologically active non-protein-bound sex hormones (22). The disturbance of sex hormone levels associates with various kinds of diseases in both males and females, including male hypogonadism and polycystic ovary syndrome (23, 24). Furthermore, it has been proven that abnormal concentrations of sex hormones are risk factors for several chronic health conditions: metabolic syndrome, diabetes, cognitive disorder, osteoporosis, and cardiovascular disease (25–29).

However, due to the limited studies, the relationship between inflammation-related indexes and sex hormones remains unclear and requires further investigation. In this study, we aimed to explore the association by adjusting covariates between inflammation-related index and sex hormones, including total testosterone (TT), estradiol (E_2_), SHBG, FAI and TT/E_2_ using data of two cycles (2013–2016) from the U.S. National Health and Nutrition Examination Survey (NHANES). We hypothesized that inflammation-related indexes might contribute to changes in sex hormone concentrations and further constructed multiple statistical models to systemically evaluate the association between inflammation-related indexes and sex hormones in American general adult population.

## 2 Methods

### 2.1 Source of data

The National Health and Nutrition Examination Survey (NHANES) was conducted by National Center for Health Statistics (NCHS) and the Centers for Disease Control/Prevention (CDC) aiming for evaluating general American health and nutritional status through 2-years cycle examinations and interviews (30). It has been lasted for 10 years and brought into more than 100,000 participants. All of the open data was obtained from the official website of NHANES (https://www.cdc.gov/nchs/nhanes/index.htm) which is updated biennially. Institutional Review Board (IRB) and NCHS Research Ethics Review Board (ERB) granted research authorization, thus no more permission was required.

### 2.2 Research population

**Figure 1:**
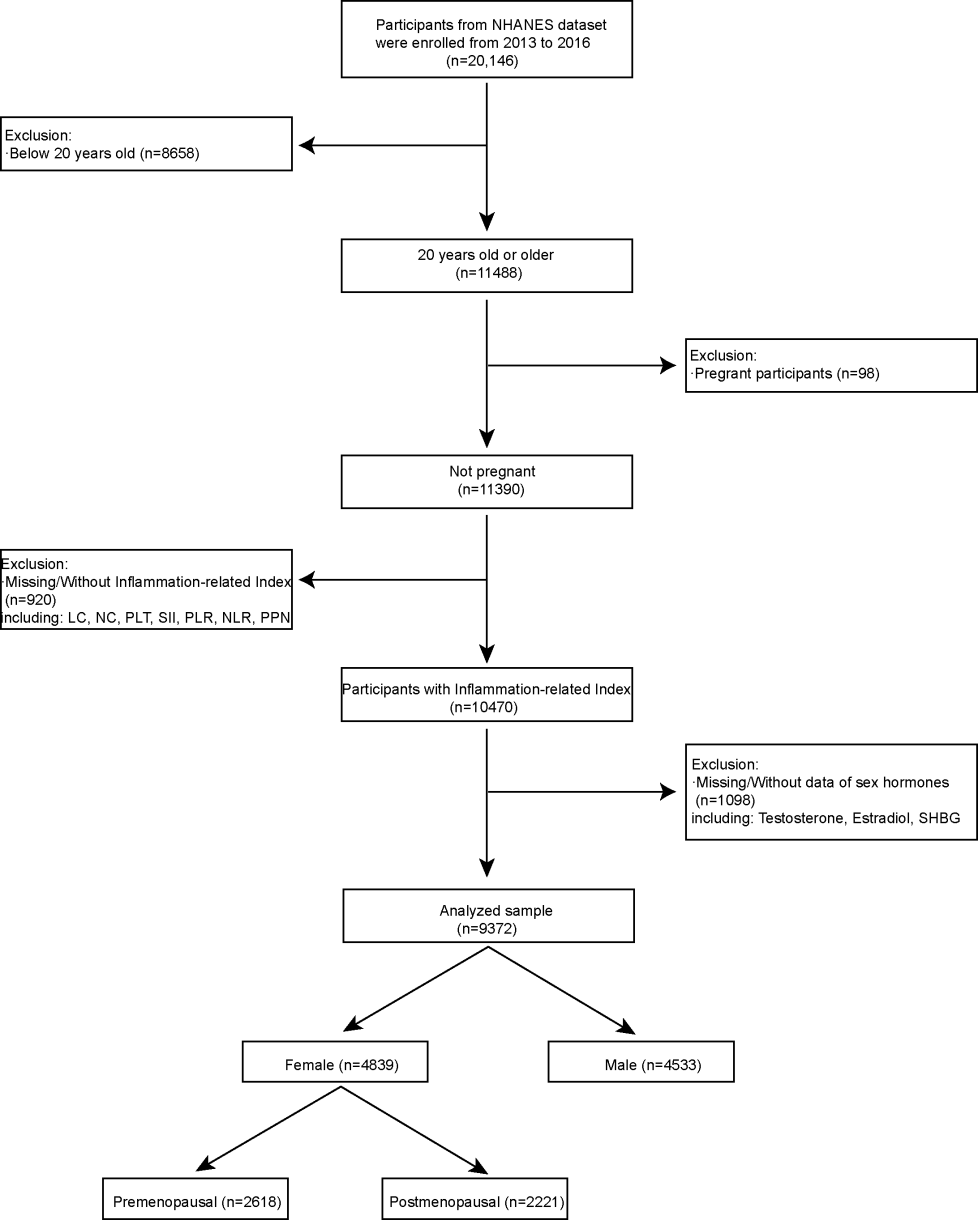
Flowchart of the research populations: NHANES 2013–2016.

Since 1999, NHANES published their data biennially (31). Complete data of sex hormone measurement only released in two cycles from 2013 to 2016, thus we excluded the data of other cycles. In our research, we included independent variable of inflammation-related indexes and dependent variable of sex hormones. We also included age, race, poverty to income ratio, education level, marital status, body mass index, comorbidities data as covariant into our research for the secondary analysis. We set following inclusion criteria for the eligible participants (1): 20 years old or older (n = 11488) (2); Not pregnant participants (n = 11390) (3); Participants with inflammation-related index (n =10470) (4); Participants with sex hormones. We also set exclusion criteria (1): below 20 years old (n = 8658) (2); pregnant participants (n =98) (3); Missing/Without Inflammation-related Index including LC, NC, PLT, SII, PLR, NLR, PPN (n = 920) (4); Missing/Without sex hormones including Testosterone, Estradiol and SHBG (n = 1098); We have included 9372 participants out of 20146 into our research through above criteria for the further analysis (**Figure 1**). Among them, 4839 participants were female and 4533 participants were male. In addition, the entirety of our research complied with the World Medical Association’s Helsinki Declaration (32).

### 2.3 Menopausal status definitions

We identified menopausal status based on the self-reported of the reproductive health questionnaire. We identified the postmenopausal status were female who answered “no” to the question “Have you had at least one menstrual period in the past 12 months?” and then answered “hysterectomy” or “menopause/change of life” to the question “What is the reason that you have not had a period in the past 12 months?”. The details of the self-reported reproductive health questionnaire are available on the NHANES website. Among them, there are 2221 postmenopausal females and 2618 premenopausal females.

### 2.4 Sex hormones measuring

CDC-developed routine chapter analyzes the isotope dilution liquid chromatography tandem mass spectrometry (ID-LC-MS/MS) technology for simultaneous detection of total testosterone and estradiol in blood, following dissociation from binding proteins and elimination of possibly interfering substances. The technique was designed for a high sample throughput and exhibits great exactness over a number of years. Measuring the chemiluminescence of the reaction product of SHBG with an immune antibody that happens after two incubation periods and subjected to a magnetic field are used to quantify SHBG. A photomultiplier tube can detect the chemiluminescent reaction that takes place when the microparticles are captured on an electrode. The results are compared to a calibration curve that is specific to the device and lot. Free testosterone can be calculated by detecting the concentration of total testosterone, SHBG and albumin. The ratio of Testosterone to Estradiol was used to measure the percentage of sexual hormones. Calculate free androgen index% (FAI)=(TT/SHBG) × 100. Measuring testosterone level on the basis of correcting SHBG abnormality is a good indicator for evaluating hyperandrogenism. The specifics of measurement may be found on the NHANES website in the laboratory protocol handbook. The detection limits were constant for all of the analytes in the data set. The value of results below the lower limit of detection (LLOD) is the lower limit of detection divided by the square root of 2 [LLOD/sqrt (2)]. The lower limit of detection for Testosterone, total (ng/dL), Estradiol (pg/mL) and Estradiol (pg/mL) are: 0.75 ng/mL*, 0.75 ng/mL*, 0.800 nmol/L. Based on the NHANES protocol, we listed the literature normal ranges of total sex hormones in the **Table S8**. We also defined the six kinds of sex hormones disorder status including Testosterone deficiency, Excessive Testosterone, Estradiol deficiency, Excessive Estradiol, SHBG deficiency, Excessive SHBG according to sex hormones normal ranges.

### 2.5 Inflammation-related Index measurement

On the NHANES MEC, samples of whole blood are analyzed. The NHANES Laboratory Procedures Manual (LPM) discusses in depth specimen collecting and processing. As a fundamental Inflammation-related index, we used the platelet count (PLT), neutrophil count (NC), and lymphocyte count (LC). For a more complete examination of the connection between inflammation-related indexes and sex hormones, we computed systemic immune-inflammation index (SII), platelet-to-lymphocyte ratio (PLR), neutrophil-to-lymphocyte ratio (NLR), and the product of platelet count and neutrophil count (PPN). PLT, NC, and LC were measured at a concentration of 1000 cells/uL. SII was computed by multiplying PLT by (NC/LC). PLR was determined by dividing PLT by LC. NC/LC was used to compute NLR. PPN was determined by multiplying PLT by NC. The detection limits were constant for all of the analytes in the data set. None of the results were below the limits of detection.

### 2.6 Covariates

Based on what we already knew from other studies (31, 33), we also included other factors that could affect the levels of sex hormones. In more detail, sociodemographic variables included age (in years), the ratio of poverty to income, race or ethnicity, level of education, and whether or not a person was married. Also, other covariates included drinking status (had at least 12 drinks of alcohol in the past year) and smoking status (smoked at least 100 cigarettes in their lifetime). Finally, we bring body mass index (Kg/m^2^) as a piece of information from a medical exam and a person’s life history into our research. On the NHANES website, you can find variables that are more complicated.

### 2.7 Statistical analysis

We employed a Systematic statistical analysis between inflammation-related indexes and sex hormone levels according to CDC standards (https://www.cdc.gov/nchs/nhanes/index.htm). For statistical analysis, the NHANES analytic guidelines were used (https://wwwn.cdc.gov/nchs/nhanes/tutorials/default2.aspx ). We utilized the mobile examination center (MEC) exam weight (WTMEC2YR) for weighted analysis since some of the variables in this research were gathered at the MEC. In addition, the sample weight utilized in the final analysis was equivalent to double the value of “WTMEC2YR” since two NHANES survey cycles were merged. The weighted mean and standard error (SE) (continuous variables) as well as the weighted percentage (categorical variables) represented the characteristics of the sample at participant baseline. If it is a continuous variable, the Kruskal-Wallis rank sum test was used. If the number of theoretical counting variables is fewer than 10, Fisher’s exact probability test was employed. In the case of categorical data, the p-value was calculated using weighted chi-square (**Table 1**). Next, we visualized the data distribution using the “ggplot2” R page and analyzed comparison data using unpaired T-test by “Prism8”. Using multivariable weighted linear regression models, the relationship between the inflammation-related indexes and sex hormone levels was evaluated. Models of association between inflammation-related index and sex hormones in female postmenopausal group and premenopausal group were constructed by multivariable weighted linear regression models. We total constructed three models. Model 1 was unadjusted model. Model 2 adjusted for age, race, education, marital status, family income to poverty ratio. Model 3 adjusted for age, race, education, marital status, family income to poverty ratio, body mass index (kg/m^2^), smoking and drinking. We aimed to explore independent relationships between inflammation-related indexes and sex hormones by adjusting covariates. Smooth curve fitting and generalized additive models were used to describe the nonlinear link between the inflammation-related indexes and sex hormones. Then, we developed the machine learning XGBoost algorithm model to investigate the relative impact of inflammation-related index on sex hormone concentrations (33). Using stratified multivariate logistic regression, we developed a subgroup study to discover the stratified relationships between inflammation-related indexes and sex hormones. Using logistic regression, we investigated the association between inflammation-related indexes and sex hormones disorder. For the sensitivity analysis, we changed SII and others inflammation-related indexes to categorical variables (Q1-Q4). R software (Version 4.0.2) and the R package (http://www.R-project.org , The R Foundation ) were used to perform all types of statistical studies (23). Our study was significantly aided by the EmpowerStats program (http://www.empowerstats.com , X&Y Solutions, Inc., Boston, MA, USA). In our research, statistical significance is determined when the p-value is less than 0.05.

**Table 1:** Baseline characteristics.

	Male	Female	Standardize diff.	P-value
N	4533	4839		
Sociodemographic variables				
Age, mean ± SD (years)	49.5 ± 17.6	49.6 ± 17.4	0.0 (-0.0, 0.0)	0.86
Poverty to income ratio, mean ± SD	2.6 ± 1.6	2.4 ± 1.6	0.1 (0.1, 0.1)	<0.001
Race/Ethnicity (%)			0.1 (0.0, 0.1)	0.004
Mexican American	712 (15.7%)	799 (16.5%)		
Other Hispanic	466 (10.3%)	602 (12.4%)		
Non-Hispanic White	1755 (38.7%)	1786 (36.9%)		
Non-Hispanic Black	884 (19.5%)	953 (19.7%)		
Other race/ethnicity	716 (15.8%)	699 (14.4%)		
Education (%)			0.1 (0.1, 0.2)	<0.001
Less than 9th grade	463 (10.2%)	501 (10.4%)		
9-11th grade	608 (13.4%)	566 (11.7%)		
High school graduate	1062 (23.4%)	998 (20.6%)		
AA degree	1246 (27.5%)	1595 (33.0%)		
College graduate	1152 (25.4%)	1176 (24.3%)		
Marital status (%)			0.3 (0.3, 0.4)	<0.001
Married	2600 (57.4%)	2277 (47.1%)		
Widowed	163 (3.6%)	508 (10.5%)		
Divorced	401 (8.8%)	634 (13.1%)		
Seperated	112 (2.5%)	192 (4.0%)		
Never married	865 (19.1%)	851 (17.6%)		
Living with partner	390 (8.6%)	375 (7.7%)		
Don’t know	2 (0.0%)	2 (0.0%)		
Medical examination and personal life history				
Body mass index, mean ± SD (Kg/m2)	28.7 ± 6.1	30.0 ± 7.8	0.2 (0.1, 0.2)	<0.001
Comorbidities (%)			0.5 (0.5, 0.6)	<0.001
Had at least 12 alcohol drinks/1 year?	3458 (81.9%)	2546 (58.4%)		
Yes	757 (17.9%)	1811 (41.5%)		
No				
Smoked at least 100 cigarettes in life			0.4 (0.4, 0.4)	<0.001
Yes	2383 (52.6%)	1596 (33.0%)		
No	2145 (47.3%)	3238 (66.9%)		
Sex hormones				
Testosterone, mean ± SD (ng/dL)	418.3 ± 186.8	23.3 ± 20.9	3.0 (2.9, 3.0)	<0.001
Estradiol, mean ± SD (pg/mL)	24.9 ± 9.9	60.7 ± 200.7	0.3 (0.2, 0.3)	<0.001
SHBG, mean ± SD (nmol/L)	45.1 ± 25.9	73.4 ± 50.5	0.7 (0.7, 0.7)	<0.001
FAI, mean ± SD	1099.1 ± 598.3	42.4 ± 49.1	2.5 (2.4, 2.5)	<0.001
Testosterone/Estradiol, mean ± SD	14.3 ± 8.8	1.3 ± 2.2	2.0 (2.0, 2.1)	<0.001
Immune-inflammation index				
Lymphocyte, mean ± SD(1000 cells/uL)	2.1 ± 1.8	2.3 ± 1.1	0.1 (0.0, 0.1)	<0.001
Neutrophils, mean ± SD(1000 cells/uL)	4.2 ± 1.7	4.4 ± 1.8	0.1 (0.0, 0.1)	<0.001
Platelet, mean ± SD(1000 cells/uL)	222.8 ± 55.6	251.2 ± 63.0	0.5 (0.4, 0.5)	<0.001
SII, mean ± SD	492.3 ± 316.7	526.7 ± 336.6	0.1 (0.1, 0.1)	<0.001
PLR, mean ± SD	115.7 ± 45.6	121.0 ± 46.6	0.1 (0.1, 0.2)	<0.001
NLR, mean ± SD	2.2 ± 1.3	2.1 ± 1.1	0.1 (0.1, 0.1)	<0.001
PPN, mean ± SD	963.5 ± 512.7	1126.1 ± 631.0	0.3 (0.2, 0.3)	<0.001

If it is a continuous variable, it was obtained by Kruskal Wallis rank sum test. For those continuous variables having a theoretical number < 10, Fisher’s exact probability test was employed to calculate the p-value. In the case of categorical data, the p-value was calculated using weighted chi-square.

## 3 Results

### 3.1 Baseline characteristics of research participants

**Figure 1** depicted the participant selection flowchart. Total 9372 participants out of 20146 was fitted into our research. We divided all participants into a second grouping respectively based on their gender. **Table 1** showed a weighted distribution of the baseline characteristics of selected American groups from the NHANES 2013-2016 survey. There were 4533 male participants and 4839 female participants. For secondary analysis, we added age, race, poverty-to-income ratio, education level, marital status, body mass index, and comorbidities data as covariant. Index of sex hormones included testosterone, estradiol, SHBG, FAI, and TT/E_2_. Platelet count (PLT), neutrophil count (NC), lymphocyte count (LC), systemic immune-inflammation index (SII), platelet-to-lymphocyte ratio (PLR), neutrophil-to-lymphocyte ratio (NLR), and the product of platelet count and neutrophil count (PPN) comprised the immune-inflammation indexes. All indicators of sex hormones and the inflammation-related indexes revealed statistically significant between the two gender groups differences with p values less than 0.05. Intriguingly, the distribution of the ratio of poverty to income, race, education level, marital status, body mass index, drinking status, and smoking status differed statistically between the two gender groups.

### 3.2 Distribution and comparison analysis

**Figure 2:**
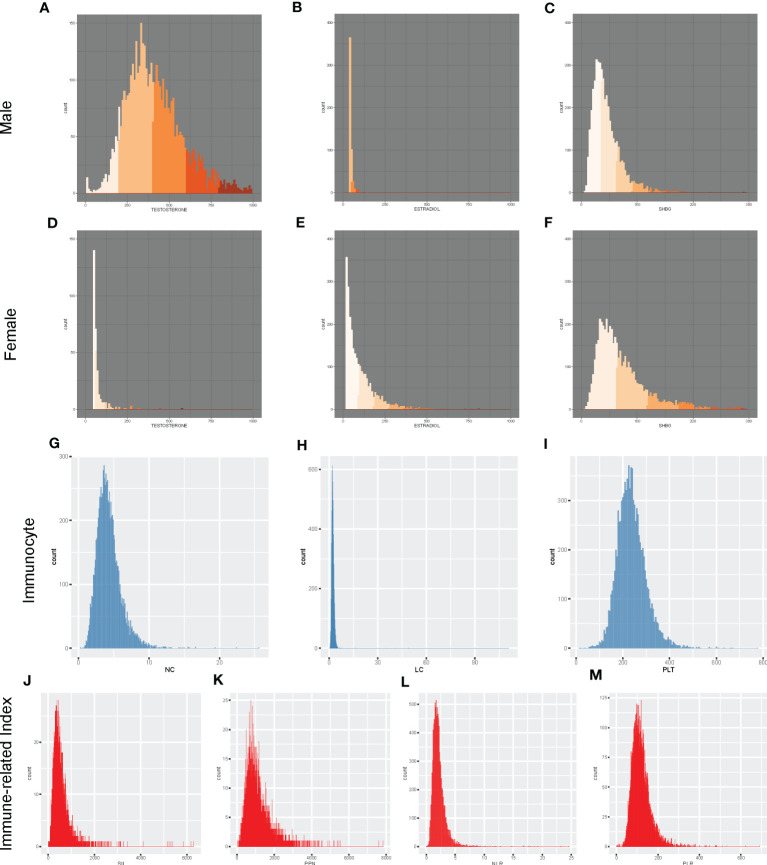
Distribution of sex hormones and inflammation-related index among American population. **(A)** Male Testosterone; **(B)** Male Estradiol; **(C)** Male SHBG; **(D)** Female Testosterone; **(E)** Female Estradiol; **(F)** Female SHBG; **(G)** NC; **(H)** LC; **(I)**PLT; **(J)** SII; **(K)** PPN; **(L)** NLR; **(M)** PLR. NC PC, NC, and LC were measured in 1000 cells/uL. LC, lymphocyte count; NC, neutrophil count; NLR, neutrophil-to-lymphocyte ratio; PLT, platelet count; PLR, platelet-to-lymphocyte ratio; PPN, the product of platelet count and neutrophil count; SII, systemic immune-inflammation index.

**Figure 3:**
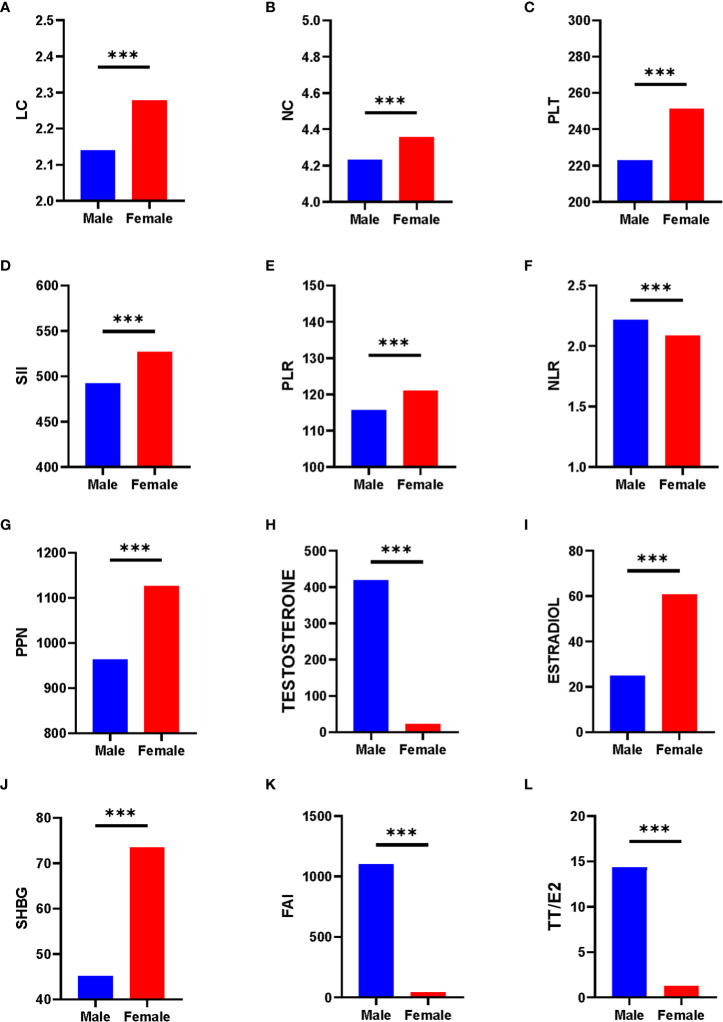
Comparison of sex hormones and inflammation-related index between various gender. **(A)** LC; **(B)** NC; **(C)** PLT; **(D)** SII; **(E)** PLR; **(F)** NLR; **(G)** PPN; **(H)** Testosterone; **(I)** Estradiol; **(J)** SHBG; **(K)** FAI; **(L)** TT/E_2_. *** mean p < 0.0001.

To have a clearer understanding of the data distribution, we have represented the data using histograms (**Figure 2**). We discovered that graphs of the dependent variable of sex hormones for different genders showed distinct distribution forms. For the independent variable Inflammation-related indexes, we illustrated its distribution and provided data in (**Table S1**). The histogram revealed that the distribution of all inflammatory indicators was not normal distribution. To test the normality of the inflammation-related index distribution, we utilized five statistical tests: the Anderson-Darling normality test, the Cramer-von Mises normality test, the Lilliefors (Kolmogorov-Smirnov) normality test, the Pearson chi-square normality test, and the Shapiro-Francia normality test (**Table S2**). In both genders, the distributions of SII, PLR, NLR, PPN, PLT, NC, and LC were exhibited right-skewed patterns. Hence, log2-transformation was applied conducting regression analysis and other analyses. Subsequently, we performed comparison analysis and displayed the results between the various sex hormones indexes and the inflammation-related indexes in both genders (**Figure 3**). Inflammation-related indexes of LC, NC, PLT, SII, PLR, and PPN were statistically greater in females than in males, although NLR was statistically lower. Regarding the sex hormones indexes, we discovered that males had greater blood levels of testosterone, FAI, and TT/E_2_, while females had higher levels of estradiol and SHBG. The potential relationship between inflammation-related indexes and sex hormones must be analyzed separately.

### 3.3 Associations between inflammation-related index and sex hormones

We further investigated the possible association between the inflammation-related indexes and sex hormones using multivariable weighted linear regression models. The connection between inflammation-related indexes and sex hormones in American males was presented in **Table 2**. In model 1, when no variables were accounted for, we discovered that every component of the inflammation-related indexes was correlated with at least one component of the sex hormones indexes in males. Even after adjusting for all variables, these correlations remained. SII, NLR, PPN, LC, NC, and PLT were negatively linked with testosterone and SHBG in a fully adjusted model. Only PPN and PLT had a negative correlation with male estradiol levels. Surprisingly, we discovered a favorable association between SII and TT/E_2_. This relationship varies among American women (**Table 3**). In the model 1 and model 2, we discovered that inflammation-related indexes was positively associated with estradiol and FAI while negatively associated with SHBG and testosterone. In the model 3, SII, NLR, PPN, and NC were associated positively with female estradiol. The inflammation-related indexes were negatively associated with SHBG across the board. PLR, PPN, LC, and NC had a positive correlation with FAI. Overall, in the fully adjusted model, a greater number of the inflammation-related indexes were significantly associated with dominant sex hormone levels in the respective gender population. Furthermore, we constructed the model of the association between Eosinophil and sex hormones in both gender (**Table S7**). We found Eosinophil was positive associated with estradiol (β=4.2, (2.7,5.8), p<0.001) and negative associated with SHBG (β=-1.7, (-2.6, -0. 9), p=0.001).

**Table 2:** Association between inflammation-related index and sex hormones in American male.

Index	Outcome	Model1				Model 2				Model3			
		β	95%CI low	95%CI upp	p-value	β	95%CI low	95%CI upp	p-value	β	95%CI low	95%CI upp	p-value
Log2-SII	Testosterone	-28.5	-35.6	-21.5	<0.001	-24.8	-32.2	-17.4	<0.001	-15.7	-22.8	-8.5	<0.001
	Estradiol	-0.7	-1.1	-0.4	<0.001	-0.5	-0.9	-0.1	0.026	-0.4	-0.8	0	0.042
	SHBG	-1.1	-2.1	-0.1	0.028	-2.9	-3.8	-2	<0.001	-2	-2.9	-1.1	<0.001
	FAI	-38.7	-61.3	-16	<0.001	5.4	-14.8	25.7	0.6	7.4	-14.1	28.9	0.5
	TT/E2	-0.3	-0.7	0	0.062	-0.1	-0.5	0.2	0.47	0.1	-0.2	0.5	0.45
Log2-NLR	Testosterone	-28.7	-36.6	-20.7	<0.001	-20.6	-29.2	-12	<0.001	-12	-20.3	-3.7	0.005
	Estradiol	-0.5	-0.9	-0.1	0.024	-0.2	-0.6	0.3	0.488	-0.1	-0.6	0.4	0.651
	SHBG	2.1	1	3.2	<0.001	-2	-3.1	-1	<0.001	-1.1	-2.2	-0.1	0.04
	FAI	-108.3	-133.7	-82.8	<0.001	-1.5	-25	22	0.9	-0.8	-25.6	24	0.95
	TT/E2	-0.7	-1	-0.3	<0.001	-0.1	-0.5	0.3	0.641	0.1	-0.3	0.5	0.565
Log2-PLR	Testosterone	7.8	-2.4	17.9	0.134	12.8	2.4	23.2	0.016	8.4	-1.6	18.4	0.099
	Estradiol	-0.8	-1.3	-0.2	0.005	-0.8	-1.4	-0.3	0.004	-0.4	-1	0.2	0.175
	SHBG	2.9	1.4	4.3	<0.001	0.4	-0.8	1.7	0.501	-0.2	-1.5	1.1	0.772
	FAI	-41.2	-73.6	-8.7	0.013	17.8	-10.5	46.1	0.218	19.7	-10.1	49.5	0.195
	TT/E2	0.3	-0.2	0.8	0.207	0.8	0.3	1.3	0.001	0.7	0.2	1.2	0.004
Log2-PPN	Testosterone	-43	-50.4	-35.6	<0.001	-45.9	-53.7	-38.2	<0.001	-31	-38.7	-23.3	<0.001
	Estradiol	-0.8	-1.2	-0.4	<0.001	-0.4	-0.8	0	0.059	-0.7	-1.1	-0.2	0.004
	SHBG	-5.9	-6.9	-4.8	<0.001	-5.1	-6.1	-4.2	<0.001	-3.6	-4.5	-2.6	<0.001
	FAI	31	7	55	0.011	3.2	-18.2	24.7	0.77	6	-17.2	29.1	0.613
	TT/E2	-0.3	-0.7	0.1	0.101	-0.7	-1.1	-0.3	<0.001	-0.2	-0.6	0.1	0.245
Log2-LC	Testosterone	-23.1	-34	-12.3	<0.001	-38.5	-49.9	-27.1	<0.001	-26.1	-37.1	-15	<0.001
	Estradiol	0.1	-0.5	0.6	0.842	0.2	-0.4	0.8	0.487	-0.3	-1	0.3	0.307
	SHBG	-9.7	-11.2	-8.3	<0.001	-3.9	-5.3	-2.5	<0.001	-2.5	-3.9	-1.1	<0.001
	FAI	156.1	121.6	190.6	<0.001	-6.1	-37.4	25.2	0.701	-5.3	-38.3	27.7	0.753
	TT/E2	0.1	-0.4	0.6	0.63	-1.1	-1.7	-0.6	<0.001	-0.7	-1.3	-0.2	0.005
Log2-NC	Testosterone	-62.9	-72.6	-53.2	<0.001	-63.2	-73.6	-52.9	<0.001	-42.1	-52.5	-31.8	<0.001
	Estradiol	-0.7	-1.2	-0.2	0.009	-0.1	-0.6	0.5	0.835	-0.5	-1.1	0.1	0.126
	SHBG	-4.8	-6.2	-3.5	<0.001	-6.3	-7.6	-5	<0.001	-4	-5.3	-2.7	<0.001
	FAI	-36.2	-67.8	-4.5	0.025	-7.4	-36.2	21.3	0.613	-6	-37.1	25.2	0.706
	TT/E2	-0.9	-1.4	-0.4	<0.001	-1.1	-1.6	-0.6	<0.001	-0.5	-1	0	0.052
Log2-PLT	Testosterone	-26.8	-41.7	-11.9	<0.001	-41.8	-57.3	-26.3	<0.001	-29.2	-44.2	-14.2	<0.001
	Estradiol	-1.6	-2.3	-0.8	<0.001	-1.5	-2.3	-0.6	<0.001	-1.5	-2.4	-0.7	<0.001
	SHBG	-12.2	-14.2	-10.2	<0.001	-6.2	-8.1	-4.3	<0.001	-5.1	-7	-3.2	<0.001
	FAI	205	157.6	252.4	<0.001	28.6	-13.7	71	0.186	34.9	-10	79.8	0.128
	TT/E2	0.9	0.2	1.6	0.012	-0.3	-1	0.5	0.502	0.2	-0.5	0.9	0.591

Model 1: Unadjusted model. Model 2: Adjusts for age, race, education, marital status, family income to poverty ratio. Model 3: Adjusts for age, race, education, marital status, family income to poverty ratio, body mass index (kg/m^2^), smoking and drinking. TT, Testosterone; E_2_, Estradiol; SHBG, sex hormone-binding globulin; FAI: free androgen index; LC, lymphocyte count; NC, neutrophil count; NLR, neutrophil-to-lymphocyte ratio; PLT, platelet count; PLR, platelet-to-lymphocyte ratio; PPN, the product of platelet count and neutrophil count; SII, systemic immune-inflammation index. Bold fonts indicate P value < 0.05.

**Table 3:** Association between inflammation-related index and sex hormones in American female.

Index	Outcome	Model1				Model 2				Model3			
		β	95%CI low	95%CI upp	p-value	β	95%CI low	95%CI upp	p-value	β	95%CI low	95%CI upp	p-value
Log2-SII	Testosterone	-0.2	-0.9	0.6	0.698	-0.2	-1	0.6	0.64	-0.7	-1.5	0.2	0.146
	Estradiol	**14.1**	**6.7**	**21.6**	**<0.001**	**15.8**	**7.7**	**24**	**<0.001**	**8.3**	**4.8**	**11.8**	**<0.001**
	SHBG	**-4.4**	**-6.3**	**-2.6**	**<0.001**	**-5.2**	**-7.1**	**-3.2**	**<0.001**	**-3.1**	**-5**	**-1.1**	**0.002**
	FAI	**2.9**	**1**	**4.7**	**0.002**	**2.9**	**1.4**	**4.4**	<0.001	0.4	-1.1	2	0.585
	TT/E2	-0.1	-0.2	0	0.064	-0.1	-0.2	0	0.059	0	-0.1	0.1	0.815
Log2-NLR	Testosterone	-0.5	-1.4	0.4	0.258	0	-1	0.9	0.929	-0.7	-1.7	0.4	0.207
	Estradiol	**15.6**	**6.9**	**24.4**	**<0.001**	**25.9**	**16.1**	**35.7**	**<0.001**	**12.8**	**8.6**	**17**	<**0.001**
	SHBG	-0.9	-3.1	1.3	0.422	-2.3	-4.7	0.2	0.066	-1.6	-4	0.7	0.172
	FAI	0.1	-2	2.3	0.911	1.9	**0.1**	**3.7**	**0.042**	-0.2	-2.1	1.6	0.8
	TT/E2	0	-0.1	0.1	0.548	-0.1	-0.2	0	0.144	0	-0.1	0.1	0.582
Log2-PLR	Testosterone	-0.6	-1.7	0.5	0.306	-0.3	-1.4	0.9	0.662	-0.5	-1.7	0.8	0.457
	Estradiol	4.2	-6.6	15.1	0.444	7.2	-4.6	19	0.23	2	-3	7	0.433
	SHBG	**3.6**	**0.9**	**6.4**	**0.009**	2.5	-0.4	5.4	0.089	0.8	-1.9	3.6	0.554
	FAI	-4.4	-7	**-1.7**	**0.001**	-3.5	-5.7	**-1.3**	**0.002**	**-3.3**	**-5.5**	**-1**	**0.004**
	TT/E2	0	-0.1	0.2	0.46	0	-0.1	0.1	0.958	0	-0.1	0.1	0.961
Log2-PPN	Testosterone	0.3	-0.4	1.1	0.379	-0.2	-1	0.6	0.58	-0.6	-1.5	0.3	0.178
	Estradiol	**14.8**	**7.4**	**22.2**	**<0.001**	**10.1**	2	**18.3**	**0.015**	**6.7**	**3.1**	**10.3**	**<0.001**
	SHBG	**-9.7**	**-11.5**	**-7.9**	**<0.001**	**-9.8**	**-11.8**	**-7.9**	**<0.001**	**-5.6**	**-7.6**	**-3.6**	**<0.001**
	FAI	**7.5**	**5.7**	**9.3**	**<0.001**	**6.1**	**4.6**	**7.6**	**<0.001**	**2.8**	**1.2**	**4.4**	**<0.001**
	TT/E2	**-0.2**	**-0.3**	**-0.1**	**<0.001**	**-0.1**	**-0.2**	**0**	**0.012**	0	-0.1	0.1	0.97
Log2-LC	Testosterone	**1.2**	**0**	**2.5**	**0.045**	-0.1	-1.4	1.2	0.887	0.1	-1.2	1.5	0.833
	Estradiol	2.1	-9.6	13.8	0.728	**-14.2**	**-27.2**	**-1.2**	**0.033**	-4.5	-10.1	1	0.111
	SHBG	**-13.5**	**-16.4**	**-10.6**	**<0.001**	**-12.1**	**-15.3**	**-8.9**	**<0.001**	**-5.7**	**-8.8**	**-2.6**	**<0.001**
	FAI	**11.9**	**9**	**14.7**	**<0.001**	**8.3**	**5.9**	**10.7**	**<0.001**	**5.5**	**3.1**	**8**	**<0.001**
	TT/E2	**-0.3**	**-0.4**	**-0.2**	**<0.001**	-0.1	-0.2	0.1	0.311	0	-0.1	0.2	0.758
Log2-NC	Testosterone	0.2	-0.8	1.2	0.676	-0.1	-1.2	1	0.825	-0.8	-2	0.4	0.198
	Estradiol	**21.5**	**11.6**	**31.4**	**<0.001**	**22.6**	**11.5**	**33.7**	**<0.001**	**14**	**9.2**	**18.9**	**<0.001**
	SHBG	**-10.8**	**-13.3**	**-8.3**	**<0.001**	**-11.6**	**-14.3**	**-8.9**	**<0.001**	**-6.7**	**-9.4**	**-3.9**	**<0.001**
	FAI	**8.7**	**6.2**	**11.1**	**<0.001**	**8.3**	**6.3**	**10.4**	**<0.001**	**4**	**1.8**	**6.1**	**<0.001**
	TT/E2	**-0.2**	**-0.3**	**-0.1**	**0.002**	**-0.1**	**-0.3**	**0**	**0.012**	0	-0.2	0.1	0.71
Log2-PLT	Testosterone	1	-0.6	2.5	0.241	-0.7	-2.3	1	0.426	-0.7	-2.5	1.1	0.431
	Estradiol	11.9	-3.3	27.2	0.126	-8.8	-25.4	7.8	0.3	-3.3	-10.4	3.7	0.356
	SHBG	**-15.8**	**-19.6**	**-12**	**<0.001**	**-14.7**	**-18.8**	**-10.7**	**<0.001**	**-7.6**	**-11.6**	**-3.7**	**<0.001**
	FAI	**11.6**	**7.9**	**15.3**	**<0.001**	**6.5**	**3.4**	**9.5**	**<0.001**	2.5	-0.7	5.6	0.128
	TT/E2	**-0.4**	**-0.6**	**-0.2**	**<0.001**	-0.1	-0.3	0.1	0.171	0	-0.1	0.2	0.643

Model 1: Unadjusted model. Model 2: Adjusts for age, race, education, marital status, family income to poverty ratio. Model 3: Adjusts for age, race, education, marital status, family income to poverty ratio, body mass index (kg/m^2^), smoking and drinking. TT, Testosterone; E_2_, Estradiol; SHBG: sex hormone-binding globulin; FAI, free androgen index; LC, lymphocyte count; NC, neutrophil count; NLR, neutrophil-to-lymphocyte ratio; PLT, platelet count; PLR, platelet-to-lymphocyte ratio; PPN, the product of platelet count and neutrophil count; SII, systemic immune-inflammation index. Bold fonts indicate P value < 0.05.

In order to exclude the influence of premenstrual and postmenstrual status, we constructed the multivariable weighted linear regression models in both groups (**Tables S9**, **S10** **)**. For the postmenstrual group, we found inflammation-related indexes including SII, PPN, LC, NC, PLT were negatively associated with serum SHBG in the fully adjusted model, while SII and NLR were negatively associated with Testosterone. For the premenstrual group, SII, NLR, PPN and NC were positively associated with Estradiol. Thus, inflammation-related indexes represent different relationship with sex hormone levels in premenstrual and postmenstrual groups.

### 3.4 Generalized-linear analysis between Inflammation-related Index and sex hormones

**Figure 4:**
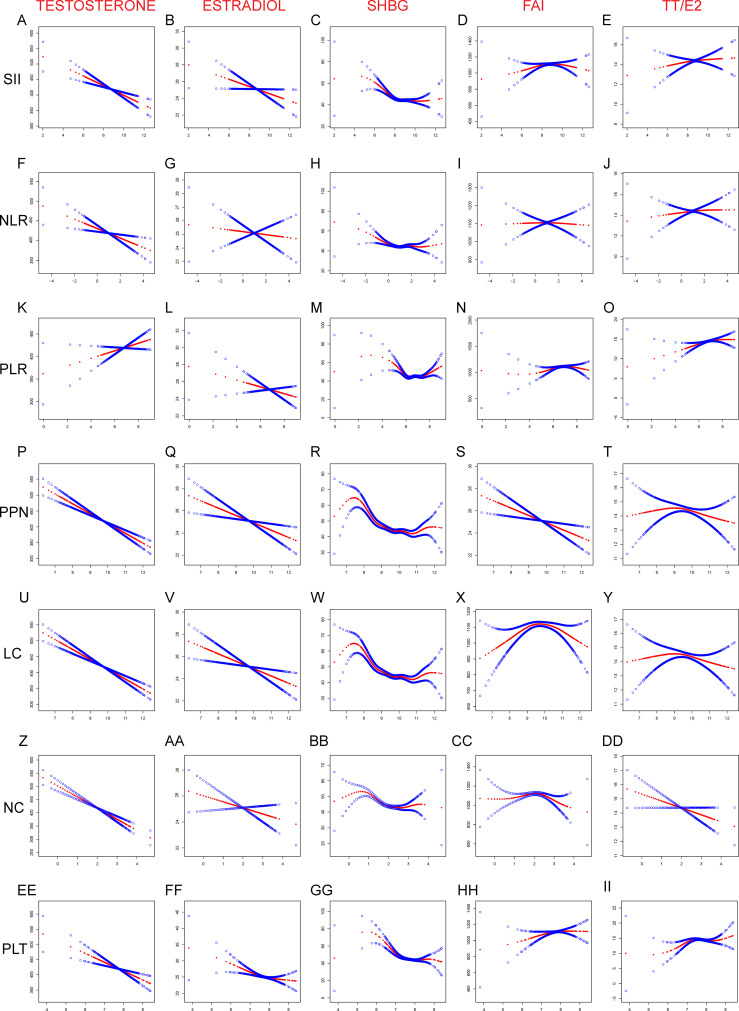
Linear and Non-linear relationship between inflammation-related index and sex hormones among American male. **(A)** TT and SII; **(B)** E_2_ and SII; **(C)** SHBG and SII; **(D)** FAI and SII; **(E)** TT/E_2_ and SII; **(F)** TT and NLR; **(G)** E_2_ and NLR; **(H)** SHBG and NLR; **(I)** FAI and NLR; **(J)** TT/E_2_ and NLR; **(K)** TT and PLR; **(L)** E_2_ and NLR; **(M)** SHBG and NLR; **(N)** FAI and NLR; **(O)** TT/E_2_ and NLR; **(P)** TT and PPN; **(Q)** E_2_ and PPN; **(R)** SHGB and PPN; **(S)** FAI and PPN; **(T)** TT/E_2_ and PPN; **(U)** TT and LC; **(V)** E_2_ and LC; **(W)** SHBG and LC; **(X)** FAI and LC; **(Y)** TT/E_2_ and LC; **(Z)** TT and NC; **(AA)** E2 and NC; **(BB)** SHBG and NC; **(CC)** FAI and NC; **(DD)** TT/E2 and NC; **(EE)** TT and PLT; (FF) E2 and PLT; (GG) SHBG and PLT; (HH) FAI and PLT; (II) TT/E2 and PLT; SII, PLR, NLR, PPN, PC, NC, and LC were considered continuous variables and conducted log2 conversion. Generalized-linear models adjusts for age, race, education, marital status, family income to poverty ratio, body mass index (kg/m^2^), smoking and drinking. TT, Testosterone; E_2_, Estradiol; SHBG, sex hormone-binding globulin; FAI, free androgen index; LC, lymphocyte count; NC, neutrophil count; NLR, neutrophil-to-lymphocyte ratio; PLT, platelet count; PLR, platelet-to-lymphocyte ratio; PPN, the product of platelet count and neutrophil count; SII, systemic immune-inflammation index.

**Figure 5:**
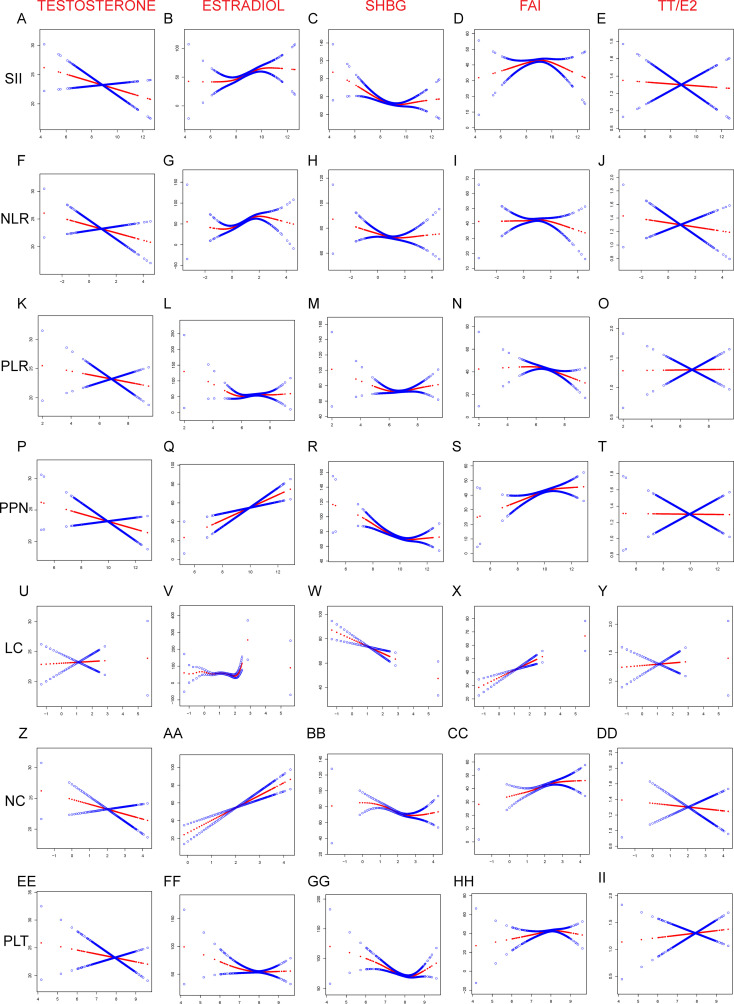
Linear and Non-linear relationship between inflammation-related index and sex hormones among American female. **(A)** TT and SII; **(B)** E_2_ and SII; **(C)** SHBG and SII; **(D)** FAI and SII; **(E)** TT/E_2_ and SII; **(F)** TT and NLR; **(G)** E_2_ and NLR; **(H)** SHBG and NLR; **(I)** FAI and NLR; **(J)** TT/E_2_ and NLR; **(K)** TT and PLR; **(L)** E_2_ and NLR; **(M)** SHBG and NLR; **(N)** FAI and NLR; **(O)** TT/E_2_ and NLR; **(P)** TT and PPN; **(Q)** E_2_ and PPN; **(R)** SHGB and PPN; **(S)** FAI and PPN; **(T)** TT/E_2_ and PPN; **(U)** TT and LC; **(V)** E_2_ and LC; **(W)** SHBG and LC; **(X)** FAI and LC; **(Y)** TT/E_2_ and LC; **(Z)** TT and NC; **(AA)** E2 and NC; **(BB)** SHBG and NC; **(CC)** FAI and NC; **(DD)** TT/E2 and NC; **(EE)** TT and PLT; **(FF)** E2 and PLT; **(GG)** SHBG and PLT; **(HH)** FAI and PLT; **(II)** TT/E2 and PLT; SII, PLR, NLR, PPN, PC, NC, and LC were considered continuous variables and conducted log2 conversion. Generalized-linear models adjusts for age, race, education, marital status, family income to poverty ratio, body mass index (kg/m^2^), smoking and drinking. TT, Testosterone; E_2_, Estradiol; SHBG, sex hormone-binding globulin; FAI, free androgen index; LC, lymphocyte count; NC, neutrophil count; NLR, neutrophil-to-lymphocyte ratio; PLT, platelet count; PLR, platelet-to-lymphocyte ratio; PPN, the product of platelet count and neutrophil count; SII, systemic immune-inflammation index.

In addition, we further examined the nature of the association between inflammation-related markers and sex hormone levels in the blood, focusing on potential linear and non-linear relationships. We discovered linear associations between testosterone and estradiol and the inflammation-related indexes in American males (**Figure 4**). In particular, we found that only testosterone and PLR exhibited a positive association; all other interactions were negative. Furthermore, we identified non-linear associations between the inflammation-related indexes and SHBG, FAI, and TT/E_2_ that were U-shaped. Inflammation-related indexes demonstrated a negative linear connection with testosterone in American women (**Figure 5**). Although PPN and NC had a positive linear connection with Estradiol, other inflammation-related indices demonstrated a positive nonlinear link. Only the LC revealed a negative linear association with SHBG, while the other inflammation-related indexes had a U-shaped relationship. PPN had a negative linear correlation with FAI, while NC demonstrated a negative linear correlation with TT/E_2_. Other inflammation-related indexes all shown a U-shaped correlation with FAI and TT/E_2_. The graphic displayed all of the particular outcomes

### 3.5 XGBoost algorithm models reveal inflammation-related index relative importance

**Figure 6:**
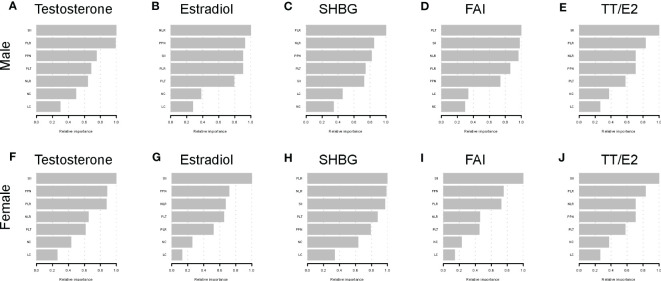
XGBoost models indicate the corresponding variable importance score of the inflammation-related indexes on sex hormones. **(A)** Testosterone in male; **(B)** Estradiol in male; **(C)** SHBG in male; **(D)** FAI in male; **(E)** TT/E_2_ in male; **(F)** Testosterone in female; **(G)** Estradiol in female; **(H)** SHBG in female; **(I)** FAI in female; **(J)** TT/E_2_ in female; The X-axis indicates the importance score, the relative number of a variable used to distribute the data; the Y-axis shows the Inflammation-related Index.

In order to determine the relative significance of the inflammation-related indexes on sex respective hormones, we developed the XGBoost method, which is a machine learning model, to determine the answer (**Figure 6**). For females, SII was the most crucial index for testosterone, estradiol, FAI, and TT/E_2_, whereas PLR was the most critical index for SHBG. SII was the most important indicator in determining the blood testosterone level and TT/E_2_ ratio for males. Also, we observed that PLR was the most significant index for estradiol and SHBG. Curiously, PLR was also the most significant index in the SHBG for male Americans.

### 3.6 Stratified analysis

We have listed the subgroup analysis about the association between the inflammation-related indexes and sex hormones among American males in the **Table S3** and females in the **Table S4**. The results revealed a negative relationship between the inflammation-related indexes and sex hormones in older and BMI>25 populations. The relationship results were comparable to the regression outcomes. SII was the most predictive index for male testosterone and female estradiol levels. There are differing degrees of relevancy between populations of varying ages and BMIs. Using the stratified analysis, we revealed the group at high risk due to the influence of inflammations status.

### 3.7 Association between Inflammation-related Indexes and sex hormones disorder

We listed the literature normal ranges of total sex hormones in the **Table S8** based on the NHANES protocol. We defined the six kinds of sex hormones disorder status including Testosterone deficiency, Excessive Testosterone, Estradiol deficiency, Excessive Estradiol, SHBG deficiency, Excessive SHBG according to sex hormones normal ranges. Then, we investigated the association between inflammation-related indexes and sex hormones disorder using logistic regression in male group, premenstrual and postmenstrual female groups (**Tables S11**–**S13**). For the male group, we found inflammation-related indexes of SII, NLR, PPN, NC, PLT were associated with Testosterone deficiency (<280 ng/dL) in the fully adjusted model. For the postmenstrual group, the relationships between inflammation-related indexes and sex hormones were similar with male groups and the inflammation-related indexes of SII, NLR,PLR and NC were associated with Testosterone deficiency as well. For the female premenopausal group, we found various relationship and the inflammation-related Indexes of SII, NLR, PLR, PPN and NC were associated with Excessive Estradiol in the model 3. The results revealed that the inflammation-related indexes were associated with Testosterone deficiency in male and postmenstrual group. In the premenopausal group, the Inflammation-related Indexes was associated with Excessive Estradiol.

### 3.8 Sensitivity analysis

In the sensitivity analysis, SII and others inflammation-related indexes were converted from continuous variables to categorical variables (Q1-Q4). The results of the sensitivity analysis matched those studies of weighted linear regression models. Specifically, we discovered that when hormone levels rose, the influence of the inflammation-related indexes became increasingly substantial. The findings of the sensitivity analysis indicated that the quarter of the American population with higher inflammation-related indexes ere more significantly affected by inflammatory disturbance and exhibited a more pronounced variation compared to in sex hormones than the quarter with a lower inflammation-related indexes. More information on the sensitivity analysis is included in **Tables S5**, **S6**.

## 4 Discussion

The relationship between inflammation-related indexes and sex hormones in the American population was exhaustively examined by our research. We chose to provide the log2 value inflammation-related indexes for normalization. SII and other inflammation-related indexes were found to be inversely correlated with male testosterone and estradiol levels. These negative correlations were depicted as linear, although PLR was positively correlated with testosterone. In contrast, an elevated inflammation-related index was associated with a reduction in serum testosterone and an increase in estradiol concentrations in females. Both genders exhibited a decreasing trend in SHBG levels as the inflammation-related index increased. SII was the most useful indicator for female sex hormones other than SHBG. PLR was the most valuable SHBG index for both men and women. SII predicted the highest gain value for testosterone and TT/E_2_ in the male population, while NLR predicted the highest gain value for estradiol. Populations with BMI>25 and age>60 demonstrated a greater degree of relevance. Inflammation-related indexes were associated with Testosterone deficiency in male and postmenstrual group. In the premenopausal group, the inflammation-related indexes were associated with Excessive Estradiol.

Several previous studies have investigated the relationship between inflammatory indicators and sex hormones. Li et al. found that high SII levels in men were related with an increased prevalence of testosterone deficiency (TD), defined as a serum testosterone level ≤ 300ng/dl (34). M. Infante et al. observed that total testosterone levels were inversely correlated with NLR in COVID-19 hospitalized males and represented as an independent risk factor for in-hospital death (35). Zhou et al. demonstrated that NLR was negatively correlated to bioavailable testosterone (BIOT) calculated based on albumin, SHBG and testosterone after adjusting for potential confounding factors (36). All these results in males were consistent to our results to some extent.

In comparison to earlier research, our paper has numerous advantages. Firstly, just one study observed the link between the SII and low testosterone deficiency in men. Our study presented new information about the quantitative relationships between the inflammation-related indexes and a wide variety of sex hormone indicators in the American general population. We identified additional inflammatory indicators and assessed their relative significance using XGBoost models. Secondly, in contrast to prior investigations, we evaluated the connection between sex hormones and inflammatory markers obtained from Complete blood count (CBC) including PLT, NC, and LC, one of the most common examinations in clinical work. Thirdly, we evaluated various types of other sex hormone indexes in both genders to evaluate the inflammation effect completely. Finally, our research undertook a stratified analysis to access the potential impact of other factors on the association between the inflammation-related indexes and the sex hormone concentrations, which was a fundamental distinction between our study and those of the previous researchers.

The current study examined the connection between Inflammatory status and sex hormone endocrine disorder. It is critical to highlight that due to the study’s cross-sectional design, causality could not be determined. Higher inflammation-related parameters, which might indicate an inflammatory state, may have led to sex hormone imbalances. In contrast, abnormal sex hormone levels may cause alterations in inflammatory state and immune response. Although the exact mechanism behind the association between inflammation-related markers and sex hormone concentrations remains undetermined, there are several possible explanations that might involve the interaction between the immune and reproductive systems. When inflammation occurs, pro-inflammatory cytokines would typically stimulate the hypothalamic-pituitary-adrenal axis (HPA) in order to prevent the overreaction of inflammation through the anti-inflammatory effects of glucocorticoids (GC) (37, 38). During the process, corticotrophin-releasing hormone (CRH) released by the hypothalamus is a main regulator (39). In individuals with inflammatory diseases, CRH and GC are observed to be elevated, according to several studies (40, 41). Moreover, CRH affect the secretion of GH, TRH and TSH, thus influencing reproductive, growth and thyroid functions (42). While sex hormones are capable of regulating inflammation. Numerous autoimmune diseases are more common in women (43, 44). It has been believed that most immune cells express estrogen receptors and may respond to estrogen stimulation (45, 46). Androgen regulate inflammation as well by targeting immune cells to attenuate inflammation (47, 48).

Our discovery has significant implications for clinical use and future research. Firstly, the results indicated that population with high levels of inflammation-related indexes had higher potential risk of sex hormone changes and endocrine disorder, although the causality of two factor haven’t been figure out. Second, a traditional blood count test can be administered in primary health care facilities and big hospitals. SII and other inflammation-related indexes can be utilized as a novel predictive index to evaluate the likelihood of sex hormone changes and endocrine problem. Finally, a comprehensive comparison was conducted between the sex hormone indexes and inflammatory markers generated from PLT, NC, and LC, including SII, NLR, PLR, and PPN. Fourthly, we discovered that PLR was positively correlated with male testosterone, which separated PPN from other inflammation-related indexes. Fifthly, the subgroup analysis revealed that the correlation between sex hormones and inflammation-related indexes were stronger in populations with BMI>25 and age>60, indicating greater significance. Hence, specific populations in inflammatory status, such as those aged 60 and those who are overweight, should be mindful of the possibility of endocrine problem.

However, our study existed some limitations. First of all, we could not establish the causality between inflammation status and sex hormones due to the cross-sectional design of NHANES. Then we only included two-cycles population due to lack of data. Although we performed a weighted analysis, the sample size is relatively small. Thus, more prospective studies are required with larger sample sizes because the level of inflammation may have a long-term effect on sex hormones. Moreover, our analyzed based on NHANES dataset collecting from American population which may cause region bias. Finally, residual confounding of unmeasured factors may bias our results to some extent, such as medical conditions, diet, occupational status, timing of the sample collection, and medication use.

## 5 Conclusion

Overall, GLM models suggested that inflammation-related index as an independent risk factor may be responsible for sex hormone alterations in both gender. Using XGBoost models, the relative importance of sex hormones index among US adults was determined. A stratified study indicated the sex hormones at high risk for inflammation based on its status. Our study used a cross-sectional design. To validate our results and elucidate the underlying molecular pathways, more prospective and experimental research should be conducted.

## Data availability statement

The original contributions presented in the study are included in the article/**Supplementary Material**. Further inquiries can be directed to the corresponding authors.

## Author contributions

CW: Conceptualization, Data curation, Formal analysis, Methodology, Software, Visualization, Writing – original draft, Writing – review & editing. WZ, JC and QH: Conceptualization, Methodology, Writing – review & editing. LC and PZ: Validation, Writing – review & editing. CD and MX: Writing – review & editing. YH: Writing – review & editing. HG, MW and ZC: Conceptualization, Data curation, Formal analysis, Methodology, Software, Visualization, Writing – original draft, Writing – review & editing.
