# Supplementary material for: Systematic analysis between inflammation-related index and sex hormones in American adults: cross-sectional research based NHANES 2013-2016
Source: Front Immunol. 2023 May 26;14:1175764. doi: 10.3389/fimmu.2023.1175764 (PMC10250748; doi:10.3389/fimmu.2023.1175764)
Supplement: Supplementary file 1 [file Table_1.docx]

Supplementary Material

Systematic analysis between [Inflammation](https://www.frontiersin.org/journals/immunology/sections/inflammation)-related Index and sex hormones in American adults: cross-sectional research based NHANES 2013-2016

Chengcheng Wei^1†^, Wenting Zhang^2†^, QingLiu He^3†^, Li Cao^4^, Pu Zhang^1^, Changqi Deng^1^, Ming Xiong^1^, Yu Huang^1^, Miao Wang^1*^, Zhaohui Chen^1*^

^1^ Department of Urology, Union Hospital, Tongji Medical College, Huazhong University of Science and Technology, Wuhan

^2^ Department of Obstetrics and Gynecology, Union Hospital, Tongji Medical College, Huazhong University of Science and Technology, Wuhan, Hubei, China

^3^ Department of Urology, The Second Affiliated Hospital of Fujian Medical University, Quanzhou 362000, China

^4^ Department of Orthopaedic, Union Hospital, Tongji Medical College, Huazhong University of Science and Technology, Wuhan, Hubei, China

†These authors have contributed equally to this work

*** Correspondence:**Zhaohui Chen: zhaohuichen@hust.edu.cn
Miao Wang: [wangmiaotj@163.com](mailto:wangmiaotj@163.com)

**Supplementary Table S1.** Distribution of [Inflammation](https://www.frontiersin.org/journals/immunology/sections/inflammation)-related Index among American

**Supplementary Table S2.** Normality test of [Inflammation](https://www.frontiersin.org/journals/immunology/sections/inflammation)-related Index among American.

**Supplementary Table S3** Subgroup analysis of the association of [Inflammation](https://www.frontiersin.org/journals/immunology/sections/inflammation)-related Index and sex hormones in Male

**Supplementary Table S4** Subgroup analysis of the association of [Inflammation](https://www.frontiersin.org/journals/immunology/sections/inflammation)-related Index and sex hormones in Female

**Supplementary Table S5.** Sensitivity analysis in Male

**Supplementary Table S6.** Sensitivity analysis in Female

**Supplementary Table S7.** Association between Eosinophil and sex hormones in both gender

**Supplementary Table S8.** Literature normal ranges of total sex hormones in serum

**Supplementary Table S9.** Association between [Inflammation](https://www.frontiersin.org/journals/immunology/sections/inflammation)-related Index and sex hormones in Female postmenopausal group

**Supplementary Table S10.** Association between [Inflammation](https://www.frontiersin.org/journals/immunology/sections/inflammation)-related Index and sex hormones in Female premenopausal group

**Supplementary Table S11.** Association between [Inflammation](https://www.frontiersin.org/journals/immunology/sections/inflammation)-related Index and sex hormones disorder in Male

**Supplementary Table S12.** Association between [Inflammation](https://www.frontiersin.org/journals/immunology/sections/inflammation)-related Index and sex hormones disorder in Female postmenopausal group

**Supplementary Table S13** Association between [Inflammation](https://www.frontiersin.org/journals/immunology/sections/inflammation)-related Index and sex hormones disorder in Female premenopausal group

**Supplementary Table S1.** Distribution of [Inflammation](https://www.frontiersin.org/journals/immunology/sections/inflammation)-related Index among American.

|  | Index | N | Mean | Standard | Min | 25% digits | 50% digits | 75% digits | Max |
| --- | --- | --- | --- | --- | --- | --- | --- | --- | --- |
| Male |  |  |  |  |  |  |  |  |  |
|  | LC | 4533 | 2.1 | 1.8 | 0.3 | 1.1 | 1.3 | 1.6 | 2.0 |
|  | NC | 4533 | 4.2 | 1.7 | 0.6 | 2.1 | 2.4 | 3.1 | 4.0 |
|  | PLT | 4533 | 222.8 | 55.6 | 14.0 | 145.0 | 159.2 | 185.0 | 219.0 |
|  | SII | 4533 | 492.3 | 316.7 | 4.1 | 186.4 | 225.0 | 302.7 | 424.9 |
|  | PLR | 4533 | 115.7 | 45.6 | 1.0 | 60.4 | 70.0 | 85.7 | 108.0 |
|  | NLR | 4533 | 2.2 | 1.3 | 0.0 | 0.9 | 1.1 | 1.5 | 2.0 |
|  | PPN | 4533 | 963.5 | 512.7 | 79.8 | 368.0 | 450.8 | 616.2 | 864.8 |
| Female |  |  |  |  |  |  |  |  |  |
|  | LC | 4839 | 2.3 | 1.1 | 0.4 | 1.2 | 1.4 | 1.8 | 2.2 |
|  | NC | 4839 | 4.4 | 1.8 | 0.3 | 2.1 | 2.4 | 3.1 | 4.1 |
|  | PLT | 4839 | 251.2 | 63.0 | 18.0 | 162.0 | 180.0 | 210.0 | 244.0 |
|  | SII | 4839 | 526.7 | 336.6 | 19.5 | 192.2 | 241.4 | 332.8 | 458.9 |
|  | PLR | 4839 | 121.0 | 46.6 | 4.0 | 64.1 | 72.7 | 90.3 | 113.8 |
|  | NLR | 4839 | 2.1 | 1.1 | 0.1 | 0.9 | 1.1 | 1.4 | 1.9 |
|  | PPN | 4839 | 1126.1 | 631.0 | 33.0 | 411.3 | 500.3 | 706.9 | 983.5 |

**Supplementary Table S2.** Normality test of [Inflammation](https://www.frontiersin.org/journals/immunology/sections/inflammation)-related Index among American.

|  | Male | | | Female | | |
| --- | --- | --- | --- | --- | --- | --- |
| LC |  | | |  | | |
|  | Anderson-Darling normality test | 629.8 | <0.001 | Anderson-Darling normality test | 239.4 | <0.001 |
|  | Cramer-von Mises normality test | 112.6 | <0.001 | Cramer-von Mises normality test | 37.4 | <0.001 |
|  | Lilliefors (Kolmogorov-Smirnov) normality test | 0.3 | <0.001 | Lilliefors (Kolmogorov-Smirnov) normality test | 0.1 | <0.001 |
|  | Pearson chi-square normality test | 7189.8 | <0.001 | Pearson chi-square normality test | 7269.2 | <0.001 |
|  | Shapiro-Francia normality test | 0.2 | <0.001 | Shapiro-Francia normality test | 0.5 | <0.001 |
| NC |  |  |  |  |  |  |
|  | Anderson-Darling normality test | 57.8 | <0.001 | Anderson-Darling normality test | 66.2 | <0.001 |
|  | Cramer-von Mises normality test | 9.5 | <0.001 | Cramer-von Mises normality test | 10.7 | <0.001 |
|  | Lilliefors (Kolmogorov-Smirnov) normality test | 0.1 | <0.001 | Lilliefors (Kolmogorov-Smirnov) normality test | 0.1 | <0.001 |
|  | Pearson chi-square normality test | 1551.0 | <0.001 | Pearson chi-square normality test | 1484.9 | <0.001 |
|  | Shapiro-Francia normality test | 0.9 | <0.001 | Shapiro-Francia normality test | 0.9 | <0.001 |
| PLT |  |  |  |  |  |  |
|  | Anderson-Darling normality test | 19.8 | <0.001 | Anderson-Darling normality test | 25.2 | <0.001 |
|  | Cramer-von Mises normality test | 2.9 | <0.001 | Cramer-von Mises normality test | 4.2 | <0.001 |
|  | Lilliefors (Kolmogorov-Smirnov) normality test | 0.0 | <0.001 | Lilliefors (Kolmogorov-Smirnov) normality test | 0.1 | <0.001 |
|  | Pearson chi-square normality test | 309.9 | <0.001 | Pearson chi-square normality test | 382.7 | <0.001 |
|  | Shapiro-Francia normality test | 1.0 | <0.001 | Shapiro-Francia normality test | 1.0 | <0.001 |
| SII |  |  |  |  |  |  |
|  | Anderson-Darling normality test | 193.6 | <0.001 | Anderson-Darling normality test | 199.7 | <0.001 |
|  | Cramer-von Mises normality test | 32.7 | <0.001 | Cramer-von Mises normality test | 33.7 | <0.001 |
|  | Lilliefors (Kolmogorov-Smirnov) normality test | 0.1 | <0.001 | Lilliefors (Kolmogorov-Smirnov) normality test | 0.1 | <0.001 |
|  | Pearson chi-square normality test | 1655.7 | <0.001 | Pearson chi-square normality test | 1607.6 | <0.001 |
|  | Shapiro-Francia normality test | 0.7 | <0.001 | Shapiro-Francia normality test | 0.7 | <0.001 |
| PLR |  |  |  |  |  |  |
|  | Anderson-Darling normality test | 90.0 | <0.001 | Anderson-Darling normality test | 83.7 | <0.001 |
|  | Cramer-von Mises normality test | 14.7 | <0.001 | Cramer-von Mises normality test | 13.7 | <0.001 |
|  | Lilliefors (Kolmogorov-Smirnov) normality test | 0.1 | <0.001 | Lilliefors (Kolmogorov-Smirnov) normality test | 0.1 | <0.001 |
|  | Pearson chi-square normality test | 790.8 | <0.001 | Pearson chi-square normality test | 726.2 | <0.001 |
|  | Shapiro-Francia normality test | 0.9 | <0.001 | Shapiro-Francia normality test | 0.9 | <0.001 |
| NLR |  |  |  |  |  |  |
|  | Anderson-Darling normality test | 188.0 | <0.001 | Anderson-Darling normality test | 179.6 | <0.001 |
|  | Cramer-von Mises normality test | 32.1 | <0.001 | Cramer-von Mises normality test | 30.0 | <0.001 |
|  | Lilliefors (Kolmogorov-Smirnov) normality test | 0.1 | <0.001 | Lilliefors (Kolmogorov-Smirnov) normality test | 0.1 | <0.001 |
|  | Pearson chi-square normality test | 1617.7 | <0.001 | Pearson chi-square normality test | 1390.6 | <0.001 |
|  | Shapiro-Francia normality test | 0.7 | <0.001 | Shapiro-Francia normality test | 0.7 | <0.001 |
| PPN |  |  |  |  |  |  |
|  | Anderson-Darling normality test | 109.9 | <0.001 | Anderson-Darling normality test | 131.8 | <0.001 |
|  | Cramer-von Mises normality test | 18.1 | <0.001 | Cramer-von Mises normality test | 22.4 | <0.001 |
|  | Lilliefors (Kolmogorov-Smirnov) normality test | 0.1 | <0.001 | Lilliefors (Kolmogorov-Smirnov) normality test | 0.1 | <0.001 |
|  | Pearson chi-square normality test | 1061.9 | <0.001 | Pearson chi-square normality test | 1256.3 | <0.001 |
|  | Shapiro-Francia normality test | 0.9 | <0.001 | Shapiro-Francia normality test | 0.9 | <0.001 |

**Supplementary Table S3** Subgroup analysis of the association of [Inflammation](https://www.frontiersin.org/journals/immunology/sections/inflammation)-related Index and sex hormones in Male

| Index | Outcome | Subgroup | | Model1 | | | | Model 2 | | | | Model3 | | | |
| --- | --- | --- | --- | --- | --- | --- | --- | --- | --- | --- | --- | --- | --- | --- | --- |
|  |  |  |  | β | 95%CI low | 95%CI upp | p-value | β | 95%CI low | 95%CI upp | p-value | β | 95%CI low | 95%CI upp | p-value |
| log2-SII | Testosterone | Age | 20-40 | -30.5 | -43.5 | -17.6 | <0.001 | -27.4 | -41.4 | -13.5 | <0.001 | -14.4 | -27.8 | -1.0 | 0.035 |
|  |  |  | 40-60 | -17.1 | -29.9 | -4.4 | 0.009 | -18.4 | -31.5 | -5.2 | 0.006 | -15.7 | -28.6 | -2.8 | 0.017 |
|  |  |  | >60 | -29.5 | -40.5 | -18.4 | <0.001 | -26.1 | -37.9 | -14.4 | <0.001 | -16.3 | -27.7 | -4.8 | 0.005 |
|  |  | BMI | <25 | -36.9 | -51.7 | -22.1 | <0.001 | -28.2 | -43.6 | -12.7 | <0.001 | -31.3 | -47.4 | -15.2 | <0.001 |
|  |  |  | 25-28 | -20.7 | -33.2 | -8.2 | 0.001 | -13.6 | -27.2 | 0.1 | 0.053 | -15.5 | -29.6 | -1.4 | 0.032 |
|  |  |  | >28 | -19.4 | -28.1 | -10.8 | <0.001 | -19.7 | -29.0 | -10.4 | <0.001 | -10.8 | -20.1 | -1.5 | 0.023 |
|  | Estradiol | Age | 20-40 | -0.2 | -0.8 | 0.5 | 0.597 | 0.2 | -0.5 | 0.9 | 0.550 | -0.1 | -0.9 | 0.6 | 0.736 |
|  |  |  | 40-60 | -0.8 | -1.5 | -0.1 | 0.025 | -0.6 | -1.4 | 0.1 | 0.094 | -0.7 | -1.5 | 0.0 | 0.064 |
|  |  |  | >60 | -1.1 | -1.7 | -0.5 | <0.001 | -0.9 | -1.5 | -0.2 | 0.010 | -0.5 | -1.2 | 0.2 | 0.137 |
|  |  | BMI | <25 | -1.1 | -1.8 | -0.3 | 0.004 | -0.8 | -1.5 | 0.0 | 0.056 | -0.5 | -1.4 | 0.3 | 0.199 |
|  |  |  | 25-28 | -0.8 | -1.5 | -0.1 | 0.024 | -0.6 | -1.4 | 0.2 | 0.144 | -0.5 | -1.3 | 0.3 | 0.218 |
|  |  |  | >28 | -0.4 | -1.0 | 0.1 | 0.122 | -0.1 | -0.7 | 0.5 | 0.653 | -0.4 | -1.0 | 0.2 | 0.173 |
|  | SHBG | Age | 20-40 | -2.5 | -3.7 | -1.4 | <0.001 | -2.5 | -3.8 | -1.3 | <0.001 | -1.4 | -2.6 | -0.2 | 0.027 |
|  |  |  | 40-60 | -2.1 | -3.7 | -0.5 | 0.012 | -2.7 | -4.3 | -1.1 | 0.001 | -2.5 | -4.1 | -0.9 | 0.002 |
|  |  |  | >60 | -2.6 | -4.3 | -0.8 | 0.004 | -2.8 | -4.6 | -1.0 | 0.002 | -1.9 | -3.7 | -0.1 | 0.035 |
|  |  | BMI | <25 | 0.5 | -1.6 | 2.7 | 0.625 | -4.0 | -5.9 | -2.1 | <0.001 | -4.3 | -6.3 | -2.4 | <0.001 |
|  |  |  | 25-28 | 0.2 | -1.6 | 2.1 | 0.802 | -2.1 | -3.8 | -0.4 | 0.017 | -2.1 | -3.8 | -0.3 | 0.021 |
|  |  |  | >28 | -2.5 | -3.7 | -1.2 | <0.001 | -2.8 | -4.0 | -1.6 | <0.001 | -2.3 | -3.5 | -1.1 | <0.001 |
|  | FAI | Age | 20-40 | 11.5 | -33.5 | 56.5 | 0.617 | 4.7 | -43.6 | 53.0 | 0.847 | 1.1 | -49.9 | 52.1 | 0.967 |
|  |  |  | 40-60 | -0.8 | -35.9 | 34.3 | 0.963 | 8.8 | -28.2 | 45.8 | 0.642 | 8.4 | -31.1 | 47.9 | 0.677 |
|  |  |  | >60 | -14.3 | -33.2 | 4.7 | 0.141 | -7.4 | -25.4 | 10.6 | 0.422 | -1.8 | -20.7 | 17.2 | 0.853 |
|  |  | BMI | <25 | -68.4 | -107.4 | -29.4 | <0.001 | 29.5 | -4.2 | 63.1 | 0.087 | 34.1 | -0.9 | 69.1 | 0.057 |
|  |  |  | 25-28 | -56.6 | -106.9 | -6.4 | 0.028 | 16.7 | -31.3 | 64.7 | 0.495 | 10.4 | -39.7 | 60.5 | 0.684 |
|  |  |  | >28 | -3.3 | -37.1 | 30.4 | 0.847 | -7.7 | -37.6 | 22.1 | 0.612 | 9.1 | -21.4 | 39.7 | 0.558 |
|  | TT/E2 | Age | 20-40 | 0.0 | -0.6 | 0.6 | 0.939 | -0.3 | -1.0 | 0.4 | 0.374 | 0.2 | -0.4 | 0.8 | 0.557 |
|  |  |  | 40-60 | 0.2 | -0.3 | 0.8 | 0.388 | 0.1 | -0.4 | 0.7 | 0.656 | 0.3 | -0.3 | 0.8 | 0.387 |
|  |  |  | >60 | -0.3 | -0.9 | 0.2 | 0.238 | -0.3 | -0.9 | 0.3 | 0.362 | -0.1 | -0.7 | 0.5 | 0.746 |
|  |  | BMI | <25 | -0.5 | -1.1 | 0.2 | 0.147 | 0.0 | -0.7 | 0.7 | 0.976 | 0.0 | -0.8 | 0.7 | 0.913 |
|  |  |  | 25-28 | 0.1 | -0.5 | 0.7 | 0.845 | 0.5 | -0.2 | 1.1 | 0.165 | 0.4 | -0.3 | 1.0 | 0.280 |
|  |  |  | >28 | -0.2 | -0.7 | 0.3 | 0.365 | -0.4 | -0.9 | 0.2 | 0.159 | 0.1 | -0.4 | 0.5 | 0.838 |
| log2-NLR | Testosterone | Age | 20-40 | -23.1 | -38.3 | -7.9 | 0.003 | -19.3 | -35.8 | -2.9 | 0.021 | -12.8 | -28.6 | 2.9 | 0.110 |
|  |  |  | 40-60 | -8.5 | -23.1 | 6.2 | 0.260 | -7.9 | -23.3 | 7.5 | 0.312 | -2.1 | -17.1 | 12.9 | 0.781 |
|  |  |  | >60 | -33.5 | -46.0 | -21.0 | <0.001 | -29.1 | -42.7 | -15.5 | <0.001 | -17.0 | -30.0 | -4.0 | 0.011 |
|  |  | BMI | <25 | -42.5 | -58.9 | -26.1 | <0.001 | -29.3 | -46.8 | -11.8 | 0.001 | -32.8 | -51.1 | -14.5 | 0.001 |
|  |  |  | 25-28 | -21.0 | -35.3 | -6.7 | 0.004 | -10.2 | -26.7 | 6.2 | 0.223 | -13.1 | -30.0 | 3.8 | 0.128 |
|  |  |  | >28 | -16.5 | -26.3 | -6.8 | <0.001 | -14.6 | -25.3 | -3.8 | 0.008 | -4.8 | -15.4 | 5.9 | 0.384 |
|  | Estradiol | Age | 20-40 | -0.5 | -1.3 | 0.3 | 0.221 | -0.1 | -0.9 | 0.7 | 0.828 | -0.2 | -1.1 | 0.6 | 0.603 |
|  |  |  | 40-60 | -0.1 | -0.9 | 0.7 | 0.728 | 0.2 | -0.7 | 1.0 | 0.698 | 0.1 | -0.8 | 1.0 | 0.885 |
|  |  |  | >60 | -0.8 | -1.4 | -0.1 | 0.036 | -0.5 | -1.3 | 0.2 | 0.184 | -0.3 | -1.0 | 0.5 | 0.524 |
|  |  | BMI | <25 | -1.1 | -1.9 | -0.3 | 0.010 | -0.5 | -1.4 | 0.4 | 0.243 | -0.4 | -1.3 | 0.5 | 0.405 |
|  |  |  | 25-28 | -0.3 | -1.1 | 0.5 | 0.482 | 0.0 | -1.0 | 1.0 | 0.986 | 0.0 | -1.0 | 1.0 | 0.950 |
|  |  |  | >28 | -0.1 | -0.8 | 0.5 | 0.667 | 0.1 | -0.6 | 0.8 | 0.773 | -0.2 | -0.8 | 0.5 | 0.641 |
|  | SHBG | Age | 20-40 | -1.2 | -2.6 | 0.1 | 0.079 | -1.3 | -2.8 | 0.2 | 0.087 | -0.6 | -2.0 | 0.9 | 0.456 |
|  |  |  | 40-60 | -0.5 | -2.3 | 1.4 | 0.627 | -1.2 | -3.1 | 0.6 | 0.193 | -0.7 | -2.6 | 1.1 | 0.444 |
|  |  |  | >60 | -1.8 | -3.8 | 0.2 | 0.075 | -2.8 | -4.8 | -0.7 | 0.008 | -1.6 | -3.7 | 0.4 | 0.115 |
|  |  | BMI | <25 | 2.7 | 0.3 | 5.0 | 0.025 | -3.9 | -6.0 | -1.8 | <0.001 | -4.3 | -6.5 | -2.1 | <0.001 |
|  |  |  | 25-28 | 3.1 | 1.0 | 5.2 | 0.005 | -1.9 | -3.9 | 0.2 | 0.072 | -2.0 | -4.1 | 0.1 | 0.056 |
|  |  |  | >28 | 1.5 | 0.1 | 2.9 | 0.032 | -1.2 | -2.6 | 0.1 | 0.072 | -0.6 | -2.0 | 0.7 | 0.366 |
|  | FAI | Age | 20-40 | -5.8 | -58.5 | 47.0 | 0.831 | -5.0 | -61.8 | 51.7 | 0.862 | -12.6 | -72.5 | 47.3 | 0.680 |
|  |  |  | 40-60 | -12.2 | -52.6 | 28.2 | 0.554 | 2.5 | -40.6 | 45.6 | 0.909 | 6.8 | -39.0 | 52.7 | 0.770 |
|  |  |  | >60 | -29.1 | -50.5 | -7.7 | 0.008 | -13.4 | -34.1 | 7.4 | 0.206 | -7.8 | -29.3 | 13.7 | 0.477 |
|  |  | BMI | <25 | -124.2 | -167.0 | -81.4 | <0.001 | 20.7 | -17.5 | 58.8 | 0.288 | 25.0 | -14.8 | 64.7 | 0.219 |
|  |  |  | 25-28 | -118.8 | -176.0 | -61.6 | <0.001 | 26.1 | -31.5 | 83.8 | 0.374 | 20.2 | -39.6 | 79.9 | 0.509 |
|  |  |  | >28 | -86.6 | -124.5 | -48.7 | <0.001 | -18.8 | -53.2 | 15.7 | 0.286 | -1.9 | -37.0 | 33.1 | 0.914 |
|  | TT/E2 | Age | 20-40 | 0.3 | -0.4 | 1.1 | 0.350 | 0.0 | -0.8 | 0.8 | 0.980 | 0.1 | -0.6 | 0.8 | 0.715 |
|  |  |  | 40-60 | 0.2 | -0.5 | 0.8 | 0.630 | 0.1 | -0.6 | 0.7 | 0.848 | 0.4 | -0.3 | 1.1 | 0.232 |
|  |  |  | >60 | -0.4 | -1.0 | 0.2 | 0.187 | -0.4 | -1.0 | 0.3 | 0.322 | -0.1 | -0.8 | 0.6 | 0.689 |
|  |  | BMI | <25 | -0.8 | -1.5 | -0.1 | 0.033 | -0.1 | -0.9 | 0.7 | 0.782 | -0.1 | -0.9 | 0.7 | 0.788 |
|  |  |  | 25-28 | -0.5 | -1.2 | 0.2 | 0.137 | 0.3 | -0.5 | 1.1 | 0.427 | 0.2 | -0.6 | 1.0 | 0.607 |
|  |  |  | >28 | -0.5 | -1.1 | 0.1 | 0.083 | -0.2 | -0.8 | 0.4 | 0.533 | 0.2 | -0.4 | 0.7 | 0.490 |
| log2-PLR | Testosterone | Age | 20-40 | 27.2 | 6.9 | 47.5 | 0.009 | 28.1 | 7.0 | 49.2 | 0.009 | 22.1 | 2.3 | 42.0 | 0.029 |
|  |  |  | 40-60 | 16.3 | -1.8 | 34.5 | 0.078 | 13.2 | -5.3 | 31.7 | 0.161 | 2.6 | -15.6 | 20.8 | 0.779 |
|  |  |  | >60 | 0.6 | -14.6 | 15.8 | 0.941 | 3.7 | -12.1 | 19.5 | 0.647 | 3.0 | -12.1 | 18.1 | 0.699 |
|  |  | BMI | <25 | -16.4 | -38.5 | 5.8 | 0.147 | 0.2 | -22.4 | 22.8 | 0.988 | -4.2 | -27.5 | 19.1 | 0.725 |
|  |  |  | 25-28 | -4.3 | -21.7 | 13.1 | 0.626 | 0.1 | -18.3 | 18.4 | 0.996 | -2.4 | -21.3 | 16.5 | 0.803 |
|  |  |  | >28 | 11.3 | -1.2 | 23.7 | 0.076 | 14.6 | 1.5 | 27.6 | 0.028 | 18.3 | 5.4 | 31.2 | 0.006 |
|  | Estradiol | Age | 20-40 | 0.0 | -1.0 | 1.0 | 0.976 | 0.0 | -1.1 | 1.0 | 0.959 | 0.1 | -1.0 | 1.2 | 0.847 |
|  |  |  | 40-60 | -0.6 | -1.6 | 0.4 | 0.258 | -0.9 | -2.0 | 0.1 | 0.072 | -0.6 | -1.7 | 0.5 | 0.287 |
|  |  |  | >60 | -1.3 | -2.2 | -0.5 | 0.002 | -1.3 | -2.1 | -0.4 | 0.005 | -0.7 | -1.6 | 0.2 | 0.118 |
|  |  | BMI | <25 | -0.6 | -1.6 | 0.5 | 0.308 | -0.5 | -1.6 | 0.7 | 0.438 | -0.1 | -1.3 | 1.1 | 0.831 |
|  |  |  | 25-28 | -0.9 | -1.9 | 0.1 | 0.067 | -0.9 | -2.0 | 0.1 | 0.083 | -0.9 | -2.0 | 0.2 | 0.117 |
|  |  |  | >28 | -0.4 | -1.2 | 0.4 | 0.373 | -0.4 | -1.2 | 0.4 | 0.346 | -0.3 | -1.2 | 0.5 | 0.434 |
|  | SHBG | Age | 20-40 | 0.4 | -1.4 | 2.2 | 0.671 | 0.7 | -1.2 | 2.6 | 0.491 | 0.1 | -1.7 | 2.0 | 0.879 |
|  |  |  | 40-60 | 0.3 | -2.0 | 2.6 | 0.787 | -0.2 | -2.4 | 2.1 | 0.895 | -1.4 | -3.6 | 0.9 | 0.239 |
|  |  |  | >60 | 1.8 | -0.6 | 4.1 | 0.150 | 1.2 | -1.2 | 3.6 | 0.332 | 0.6 | -1.8 | 3.0 | 0.616 |
|  |  | BMI | <25 | 4.7 | 1.6 | 7.9 | 0.003 | -1.0 | -3.8 | 1.7 | 0.463 | -2.0 | -4.8 | 0.8 | 0.167 |
|  |  |  | 25-28 | 1.7 | -0.9 | 4.3 | 0.189 | -0.7 | -3.0 | 1.6 | 0.547 | -0.6 | -2.9 | 1.8 | 0.629 |
|  |  |  | >28 | -0.2 | -2.0 | 1.5 | 0.787 | -1.0 | -2.7 | 0.6 | 0.219 | -0.8 | -2.5 | 0.9 | 0.347 |
|  | FAI | Age | 20-40 | 53.1 | -17.2 | 123.4 | 0.139 | 34.4 | -38.4 | 107.3 | 0.355 | 38.9 | -36.8 | 114.6 | 0.314 |
|  |  |  | 40-60 | 33.9 | -16.0 | 83.7 | 0.183 | 30.8 | -21.0 | 82.6 | 0.244 | 26.3 | -29.3 | 81.8 | 0.354 |
|  |  |  | >60 | -19.8 | -45.7 | 6.0 | 0.133 | -8.3 | -32.2 | 15.6 | 0.497 | -7.1 | -32.0 | 17.8 | 0.576 |
|  |  | BMI | <25 | -99.8 | -157.6 | -42.1 | 0.001 | 32.5 | -16.6 | 81.5 | 0.195 | 50.7 | 0.3 | 101.1 | 0.049 |
|  |  |  | 25-28 | -80.6 | -150.1 | -11.1 | 0.023 | -11.4 | -75.6 | 52.8 | 0.728 | -22.0 | -88.8 | 44.8 | 0.518 |
|  |  |  | >28 | 25.0 | -23.5 | 73.4 | 0.313 | 45.2 | 3.4 | 86.9 | 0.034 | 52.9 | 10.5 | 95.2 | 0.015 |
|  | TT/E2 | Age | 20-40 | 1.0 | 0.1 | 2.0 | 0.039 | 1.0 | -0.1 | 2.0 | 0.064 | 0.9 | 0.1 | 1.8 | 0.032 |
|  |  |  | 40-60 | 1.0 | 0.3 | 1.8 | 0.008 | 1.0 | 0.2 | 1.8 | 0.012 | 0.7 | -0.1 | 1.5 | 0.076 |
|  |  |  | >60 | 0.3 | -0.4 | 1.0 | 0.429 | 0.5 | -0.3 | 1.3 | 0.229 | 0.4 | -0.4 | 1.2 | 0.299 |
|  |  | BMI | <25 | -0.5 | -1.5 | 0.5 | 0.313 | 0.5 | -0.6 | 1.5 | 0.383 | 0.6 | -0.5 | 1.6 | 0.278 |
|  |  |  | 25-28 | 0.1 | -0.8 | 0.9 | 0.881 | 0.5 | -0.3 | 1.4 | 0.209 | 0.4 | -0.5 | 1.3 | 0.338 |
|  |  |  | >28 | 0.5 | -0.2 | 1.2 | 0.168 | 0.8 | 0.0 | 1.5 | 0.048 | 0.9 | 0.2 | 1.5 | 0.012 |
| log2-PPN | Testosterone | Age | 20-40 | -54.5 | -67.1 | -41.9 | <0.001 | -52.3 | -65.9 | -38.7 | <0.001 | -30.3 | -43.8 | -16.8 | <0.001 |
|  |  |  | 40-60 | -38.4 | -51.4 | -25.3 | <0.001 | -40.2 | -53.7 | -26.7 | <0.001 | -34.6 | -48.1 | -21.2 | <0.001 |
|  |  |  | >60 | -42.9 | -55.3 | -30.4 | <0.001 | -42.8 | -56.3 | -29.4 | <0.001 | -28.7 | -41.9 | -15.4 | <0.001 |
|  |  | BMI | <25 | -37.0 | -53.1 | -20.9 | <0.001 | -37.9 | -54.2 | -21.6 | <0.001 | -40.2 | -57.3 | -23.0 | <0.001 |
|  |  |  | 25-28 | -27.7 | -41.4 | -14.1 | <0.001 | -24.1 | -39.0 | -9.1 | 0.002 | -25.0 | -40.5 | -9.4 | 0.002 |
|  |  |  | >28 | -33.7 | -42.5 | -24.8 | <0.001 | -39.2 | -48.8 | -29.6 | <0.001 | -30.7 | -40.5 | -21.0 | <0.001 |
|  | Estradiol | Age | 20-40 | 0.0 | -0.7 | 0.6 | 0.974 | 0.5 | -0.2 | 1.2 | 0.161 | -0.1 | -0.9 | 0.6 | 0.713 |
|  |  |  | 40-60 | -1.3 | -2.0 | -0.6 | 0.001 | -1.0 | -1.7 | -0.2 | 0.013 | -1.3 | -2.1 | -0.5 | 0.001 |
|  |  |  | >60 | -1.2 | -1.9 | -0.5 | 0.001 | -0.8 | -1.6 | -0.1 | 0.034 | -0.6 | -1.4 | 0.2 | 0.155 |
|  |  | BMI | <25 | -1.2 | -2.0 | -0.4 | 0.003 | -1.0 | -1.8 | -0.2 | 0.016 | -0.8 | -1.7 | 0.1 | 0.071 |
|  |  |  | 25-28 | -1.1 | -1.9 | -0.4 | 0.005 | -0.8 | -1.7 | 0.1 | 0.077 | -0.7 | -1.6 | 0.2 | 0.137 |
|  |  |  | >28 | -0.6 | -1.2 | -0.1 | 0.031 | -0.2 | -0.8 | 0.5 | 0.618 | -0.6 | -1.2 | 0.0 | 0.063 |
|  | SHBG | Age | 20-40 | -4.3 | -5.4 | -3.1 | <0.001 | -4.3 | -5.6 | -3.1 | <0.001 | -2.5 | -3.8 | -1.3 | <0.001 |
|  |  |  | 40-60 | -4.2 | -5.8 | -2.5 | <0.001 | -4.8 | -6.4 | -3.1 | <0.001 | -4.2 | -5.8 | -2.5 | <0.001 |
|  |  |  | >60 | -6.0 | -8.0 | -4.0 | <0.001 | -5.4 | -7.5 | -3.4 | <0.001 | -4.0 | -6.1 | -1.9 | 0.000 |
|  |  | BMI | <25 | -3.9 | -6.2 | -1.6 | 0.001 | -5.1 | -7.1 | -3.1 | <0.001 | -5.0 | -7.0 | -2.9 | <0.001 |
|  |  |  | 25-28 | -3.3 | -5.4 | -1.3 | 0.001 | -2.9 | -4.8 | -1.1 | 0.002 | -2.9 | -4.8 | -1.0 | 0.003 |
|  |  |  | >28 | -6.4 | -7.7 | -5.2 | <0.001 | -4.5 | -5.7 | -3.3 | <0.001 | -4.2 | -5.5 | -2.9 | <0.001 |
|  | FAI | Age | 20-40 | 5.3 | -39.3 | 49.9 | 0.817 | -2.0 | -49.8 | 45.9 | 0.936 | -6.6 | -58.2 | 45.1 | 0.804 |
|  |  |  | 40-60 | -9.9 | -46.1 | 26.4 | 0.594 | 0.0 | -38.3 | 38.2 | 0.999 | -1.7 | -43.4 | 39.9 | 0.935 |
|  |  |  | >60 | 6.4 | -15.2 | 28.0 | 0.561 | 0.0 | -20.7 | 20.7 | 0.997 | 8.9 | -13.1 | 31.0 | 0.428 |
|  |  | BMI | <25 | 12.0 | -30.5 | 54.4 | 0.581 | 31.0 | -4.8 | 66.8 | 0.090 | 27.8 | -9.6 | 65.2 | 0.146 |
|  |  |  | 25-28 | 23.5 | -31.6 | 78.6 | 0.404 | 26.0 | -26.5 | 78.6 | 0.332 | 23.3 | -32.0 | 78.7 | 0.409 |
|  |  |  | >28 | 52.7 | 17.8 | 87.5 | 0.003 | -26.7 | -57.9 | 4.5 | 0.093 | -8.6 | -41.0 | 23.7 | 0.601 |
|  | TT/E2 | Age | 20-40 | -0.7 | -1.3 | -0.1 | 0.024 | -1.0 | -1.7 | -0.4 | 0.003 | -0.2 | -0.8 | 0.4 | 0.555 |
|  |  |  | 40-60 | -0.2 | -0.7 | 0.4 | 0.570 | -0.3 | -0.9 | 0.3 | 0.270 | -0.2 | -0.8 | 0.4 | 0.556 |
|  |  |  | >60 | -0.6 | -1.3 | 0.0 | 0.044 | -0.8 | -1.4 | -0.1 | 0.031 | -0.5 | -1.2 | 0.3 | 0.206 |
|  |  | BMI | <25 | -0.1 | -0.8 | 0.6 | 0.752 | -0.2 | -0.9 | 0.6 | 0.658 | -0.3 | -1.1 | 0.5 | 0.433 |
|  |  |  | 25-28 | 0.6 | -0.1 | 1.2 | 0.083 | 0.5 | -0.2 | 1.2 | 0.198 | 0.4 | -0.3 | 1.1 | 0.263 |
|  |  |  | >28 | -0.3 | -0.9 | 0.2 | 0.200 | -1.1 | -1.6 | -0.5 | <0.001 | -0.5 | -1.1 | 0.0 | 0.033 |
| log2-LC | Testosterone | Age | 20-40 | -76.4 | -98.9 | -53.9 | <0.001 | -73.7 | -97.1 | -50.4 | <0.001 | -43.2 | -65.8 | -20.7 | <0.001 |
|  |  |  | 40-60 | -45.9 | -65.7 | -26.1 | <0.001 | -46.1 | -66.3 | -25.8 | <0.001 | -37.7 | -57.7 | -17.6 | <0.001 |
|  |  |  | >60 | -7.9 | -24.4 | 8.6 | 0.347 | -13.5 | -30.9 | 3.9 | 0.127 | -9.9 | -26.2 | 6.5 | 0.238 |
|  |  | BMI | <25 | 13.5 | -10.3 | 37.3 | 0.266 | -13.8 | -38.6 | 11.0 | 0.275 | -9.6 | -35.1 | 15.9 | 0.461 |
|  |  |  | 25-28 | -5.7 | -25.0 | 13.6 | 0.563 | -15.5 | -36.7 | 5.7 | 0.152 | -11.8 | -33.6 | 10.1 | 0.291 |
|  |  |  | >28 | -26.9 | -39.9 | -14.0 | <0.001 | -36.9 | -50.8 | -22.9 | <0.001 | -37.3 | -51.1 | -23.5 | <0.001 |
|  | Estradiol | Age | 20-40 | 0.5 | -0.6 | 1.7 | 0.385 | 0.8 | -0.4 | 2.0 | 0.167 | 0.0 | -1.3 | 1.2 | 0.966 |
|  |  |  | 40-60 | -1.0 | -2.1 | 0.1 | 0.076 | -0.6 | -1.8 | 0.5 | 0.266 | -1.2 | -2.3 | 0.0 | 0.057 |
|  |  |  | >60 | 0.4 | -0.5 | 1.3 | 0.356 | 0.5 | -0.5 | 1.5 | 0.295 | 0.2 | -0.8 | 1.2 | 0.705 |
|  |  | BMI | <25 | 0.1 | -1.0 | 1.3 | 0.811 | -0.4 | -1.6 | 0.9 | 0.578 | -0.4 | -1.7 | 0.9 | 0.526 |
|  |  |  | 25-28 | -0.3 | -1.4 | 0.8 | 0.617 | -0.1 | -1.4 | 1.1 | 0.818 | -0.1 | -1.4 | 1.2 | 0.867 |
|  |  |  | >28 | -0.4 | -1.2 | 0.5 | 0.416 | 0.0 | -0.9 | 0.9 | 0.967 | -0.3 | -1.2 | 0.6 | 0.557 |
|  | SHBG | Age | 20-40 | -5.6 | -7.6 | -3.5 | <0.001 | -5.4 | -7.5 | -3.3 | <0.001 | -3.0 | -5.1 | -1.0 | 0.004 |
|  |  |  | 40-60 | -4.5 | -7.0 | -2.0 | <0.001 | -4.2 | -6.6 | -1.7 | 0.001 | -3.1 | -5.6 | -0.6 | 0.016 |
|  |  |  | >60 | -4.6 | -7.2 | -2.0 | 0.001 | -2.9 | -5.5 | -0.3 | 0.031 | -2.0 | -4.6 | 0.5 | 0.118 |
|  |  | BMI | <25 | -9.7 | -13.0 | -6.3 | <0.001 | -1.2 | -4.2 | 1.8 | 0.436 | -0.1 | -3.2 | 3.0 | 0.948 |
|  |  |  | 25-28 | -7.2 | -10.0 | -4.3 | <0.001 | -0.9 | -3.5 | 1.7 | 0.498 | -0.8 | -3.4 | 1.9 | 0.583 |
|  |  |  | >28 | -7.9 | -9.8 | -6.1 | <0.001 | -3.0 | -4.8 | -1.3 | 0.001 | -3.2 | -5.0 | -1.4 | 0.001 |
|  | FAI | Age | 20-40 | -18.7 | -97.5 | 60.1 | 0.642 | -19.3 | -100.9 | 62.4 | 0.644 | -21.3 | -107.5 | 64.9 | 0.628 |
|  |  |  | 40-60 | -20.4 | -75.2 | 34.3 | 0.465 | -21.1 | -78.3 | 36.2 | 0.471 | -24.3 | -85.9 | 37.4 | 0.441 |
|  |  |  | >60 | 42.0 | 14.0 | 70.0 | 0.003 | 15.9 | -10.5 | 42.2 | 0.238 | 17.0 | -10.0 | 44.0 | 0.217 |
|  |  | BMI | <25 | 198.9 | 137.6 | 260.2 | <0.001 | -5.2 | -59.1 | 48.8 | 0.852 | -24.1 | -79.3 | 31.1 | 0.392 |
|  |  |  | 25-28 | 180.5 | 103.8 | 257.3 | <0.001 | 11.9 | -62.3 | 86.1 | 0.754 | 20.6 | -56.7 | 97.9 | 0.601 |
|  |  |  | >28 | 118.1 | 67.7 | 168.5 | <0.001 | -38.1 | -83.1 | 7.0 | 0.098 | -37.7 | -83.4 | 8.0 | 0.106 |
|  | TT/E2 | Age | 20-40 | -2.1 | -3.2 | -1.0 | <0.001 | -2.1 | -3.3 | -1.0 | <0.001 | -1.0 | -2.0 | 0.0 | 0.048 |
|  |  |  | 40-60 | -1.0 | -1.8 | -0.1 | 0.027 | -1.1 | -2.0 | -0.2 | 0.019 | -1.0 | -1.9 | -0.1 | 0.026 |
|  |  |  | >60 | -0.4 | -1.2 | 0.5 | 0.381 | -0.6 | -1.5 | 0.3 | 0.160 | -0.5 | -1.4 | 0.4 | 0.277 |
|  |  | BMI | <25 | 1.0 | -0.1 | 2.0 | 0.065 | -0.4 | -1.5 | 0.8 | 0.537 | -0.6 | -1.7 | 0.6 | 0.326 |
|  |  |  | 25-28 | 1.0 | 0.1 | 1.9 | 0.033 | -0.2 | -1.2 | 0.8 | 0.743 | -0.1 | -1.1 | 1.0 | 0.916 |
|  |  |  | >28 | -0.2 | -0.9 | 0.6 | 0.618 | -1.4 | -2.2 | -0.6 | 0.001 | -1.2 | -1.9 | -0.5 | 0.001 |
| log2-NC | Testosterone | Age | 20-40 | -66.4 | -82.4 | -50.3 | <0.001 | -64.0 | -81.4 | -46.5 | <0.001 | -41.2 | -58.5 | -23.9 | <0.001 |
|  |  |  | 40-60 | -45.3 | -62.3 | -28.3 | <0.001 | -46.8 | -64.6 | -28.9 | <0.001 | -33.3 | -51.3 | -15.4 | 0.000 |
|  |  |  | >60 | -73.7 | -90.8 | -56.5 | <0.001 | -75.5 | -94.5 | -56.5 | <0.001 | -49.7 | -68.6 | -30.8 | <0.001 |
|  |  | BMI | <25 | -54.6 | -74.8 | -34.5 | <0.001 | -52.2 | -73.0 | -31.3 | <0.001 | -55.7 | -77.8 | -33.6 | <0.001 |
|  |  |  | 25-28 | -40.0 | -58.3 | -21.6 | <0.001 | -32.0 | -52.9 | -11.0 | 0.003 | -33.6 | -55.3 | -11.9 | 0.003 |
|  |  |  | >28 | -48.4 | -60.2 | -36.5 | <0.001 | -54.1 | -67.0 | -41.2 | <0.001 | -41.9 | -55.1 | -28.6 | <0.001 |
|  | Estradiol | Age | 20-40 | -0.3 | -1.1 | 0.5 | 0.490 | 0.4 | -0.5 | 1.3 | 0.423 | -0.3 | -1.2 | 0.7 | 0.543 |
|  |  |  | 40-60 | -0.9 | -1.8 | 0.0 | 0.054 | -0.3 | -1.3 | 0.7 | 0.598 | -0.8 | -1.9 | 0.2 | 0.126 |
|  |  |  | >60 | -1.0 | -1.9 | 0.0 | 0.054 | -0.4 | -1.5 | 0.7 | 0.474 | -0.3 | -1.4 | 0.9 | 0.624 |
|  |  | BMI | <25 | -1.5 | -2.5 | -0.5 | 0.003 | -1.0 | -2.1 | 0.0 | 0.061 | -0.9 | -2.1 | 0.2 | 0.117 |
|  |  |  | 25-28 | -0.7 | -1.8 | 0.3 | 0.166 | -0.2 | -1.4 | 1.1 | 0.802 | -0.1 | -1.3 | 1.2 | 0.931 |
|  |  |  | >28 | -0.5 | -1.3 | 0.3 | 0.198 | 0.1 | -0.7 | 1.0 | 0.753 | -0.5 | -1.4 | 0.4 | 0.251 |
|  | SHBG | Age | 20-40 | -4.3 | -5.7 | -2.8 | <0.001 | -4.5 | -6.1 | -3.0 | <0.001 | -2.5 | -4.1 | -0.9 | 0.003 |
|  |  |  | 40-60 | -4.0 | -6.1 | -1.8 | <0.001 | -4.9 | -7.1 | -2.8 | <0.001 | -3.5 | -5.8 | -1.3 | 0.002 |
|  |  |  | >60 | -8.7 | -11.4 | -5.9 | <0.001 | -9.2 | -12.1 | -6.3 | <0.001 | -6.3 | -9.3 | -3.3 | <0.001 |
|  |  | BMI | <25 | -3.0 | -5.9 | -0.1 | 0.040 | -6.5 | -9.0 | -3.9 | <0.001 | -6.4 | -9.1 | -3.7 | <0.001 |
|  |  |  | 25-28 | -1.4 | -4.2 | 1.3 | 0.305 | -3.9 | -6.5 | -1.4 | 0.003 | -4.1 | -6.8 | -1.5 | 0.003 |
|  |  |  | >28 | -4.5 | -6.2 | -2.8 | <0.001 | -4.5 | -6.2 | -2.9 | <0.001 | -4.0 | -5.7 | -2.2 | <0.001 |
|  | FAI | Age | 20-40 | -16.4 | -73.1 | 40.4 | 0.572 | -16.7 | -77.9 | 44.5 | 0.593 | -28.0 | -94.2 | 38.3 | 0.408 |
|  |  |  | 40-60 | -31.6 | -78.6 | 15.4 | 0.187 | -13.0 | -63.5 | 37.6 | 0.616 | -9.6 | -64.8 | 45.7 | 0.735 |
|  |  |  | >60 | -8.9 | -38.7 | 21.0 | 0.561 | -7.1 | -36.6 | 22.3 | 0.635 | 6.4 | -25.1 | 37.9 | 0.689 |
|  |  | BMI | <25 | -42.5 | -95.9 | 10.8 | 0.119 | 26.1 | -19.7 | 71.9 | 0.264 | 18.3 | -30.0 | 66.6 | 0.458 |
|  |  |  | 25-28 | -31.5 | -105.6 | 42.7 | 0.406 | 54.4 | -19.2 | 128.0 | 0.148 | 54.2 | -23.0 | 131.3 | 0.169 |
|  |  |  | >28 | -30.0 | -77.0 | 17.0 | 0.212 | -61.4 | -103.5 | -19.3 | 0.004 | -37.8 | -81.7 | 6.1 | 0.091 |
|  | TT/E2 | Age | 20-40 | -0.7 | -1.5 | 0.1 | 0.078 | -1.2 | -2.0 | -0.3 | 0.007 | -0.4 | -1.2 | 0.3 | 0.264 |
|  |  |  | 40-60 | -0.5 | -1.2 | 0.2 | 0.182 | -0.7 | -1.5 | 0.1 | 0.066 | -0.2 | -1.0 | 0.6 | 0.581 |
|  |  |  | >60 | -1.2 | -2.1 | -0.4 | 0.006 | -1.5 | -2.5 | -0.5 | 0.003 | -1.0 | -2.0 | 0.1 | 0.064 |
|  |  | BMI | <25 | -0.5 | -1.4 | 0.4 | 0.295 | -0.4 | -1.4 | 0.5 | 0.392 | -0.6 | -1.6 | 0.4 | 0.235 |
|  |  |  | 25-28 | 0.1 | -0.8 | 0.9 | 0.898 | 0.3 | -0.6 | 1.3 | 0.491 | 0.3 | -0.7 | 1.3 | 0.576 |
|  |  |  | >28 | -0.9 | -1.6 | -0.2 | 0.009 | -1.5 | -2.3 | -0.8 | <0.001 | -0.8 | -1.5 | -0.1 | 0.020 |
| log2-PLT | Testosterone | Age | 20-40 | -68.8 | -97.7 | -39.9 | <0.001 | -64.1 | -94.6 | -33.7 | <0.001 | -24.2 | -53.4 | 5.0 | 0.105 |
|  |  |  | 40-60 | -48.5 | -75.6 | -21.5 | <0.001 | -54.8 | -82.3 | -27.3 | <0.001 | -62.7 | -89.6 | -35.7 | <0.001 |
|  |  |  | >60 | -14.3 | -37.4 | 8.9 | 0.226 | -17.7 | -42.1 | 6.6 | 0.153 | -13.1 | -36.6 | 10.4 | 0.274 |
|  |  | BMI | <25 | -11.1 | -45.2 | 23.0 | 0.524 | -25.8 | -59.9 | 8.4 | 0.139 | -28.8 | -64.6 | 7.0 | 0.115 |
|  |  |  | 25-28 | -19.8 | -45.6 | 6.1 | 0.134 | -26.7 | -54.5 | 1.2 | 0.061 | -26.3 | -55.1 | 2.6 | 0.075 |
|  |  |  | >28 | -26.8 | -44.3 | -9.2 | 0.003 | -37.0 | -56.0 | -17.9 | <0.001 | -29.8 | -48.8 | -10.9 | 0.002 |
|  | Estradiol | Age | 20-40 | 0.9 | -0.6 | 2.3 | 0.249 | 1.4 | -0.2 | 2.9 | 0.087 | 0.2 | -1.4 | 1.8 | 0.819 |
|  |  |  | 40-60 | -3.1 | -4.6 | -1.6 | <0.001 | -3.3 | -4.8 | -1.8 | <0.001 | -3.4 | -5.0 | -1.8 | <0.001 |
|  |  |  | >60 | -2.2 | -3.5 | -1.0 | <0.001 | -2.0 | -3.3 | -0.6 | 0.004 | -1.4 | -2.7 | 0.0 | 0.058 |
|  |  | BMI | <25 | -1.0 | -2.7 | 0.6 | 0.219 | -1.7 | -3.4 | 0.0 | 0.053 | -1.1 | -3.0 | 0.7 | 0.224 |
|  |  |  | 25-28 | -2.6 | -4.0 | -1.1 | <0.001 | -2.4 | -4.0 | -0.8 | 0.003 | -2.3 | -4.0 | -0.6 | 0.009 |
|  |  |  | >28 | -1.4 | -2.5 | -0.2 | 0.018 | -0.9 | -2.1 | 0.3 | 0.150 | -1.2 | -2.4 | 0.0 | 0.051 |
|  | SHBG | Age | 20-40 | -8.2 | -10.8 | -5.7 | <0.001 | -7.6 | -10.3 | -4.9 | <0.001 | -4.8 | -7.4 | -2.1 | 0.001 |
|  |  |  | 40-60 | -7.7 | -11.1 | -4.3 | <0.001 | -7.9 | -11.2 | -4.6 | <0.001 | -8.6 | -12.0 | -5.3 | <0.001 |
|  |  |  | >60 | -4.9 | -8.6 | -1.3 | 0.008 | -2.8 | -6.5 | 0.8 | 0.129 | -2.8 | -6.4 | 0.9 | 0.143 |
|  |  | BMI | <25 | -8.7 | -13.5 | -3.8 | <0.001 | -4.7 | -8.8 | -0.5 | 0.029 | -4.9 | -9.2 | -0.6 | 0.027 |
|  |  |  | 25-28 | -9.0 | -12.8 | -5.2 | <0.001 | -3.2 | -6.6 | 0.3 | 0.071 | -2.7 | -6.2 | 0.9 | 0.142 |
|  |  |  | >28 | -15.0 | -17.4 | -12.6 | <0.001 | -7.8 | -10.2 | -5.5 | <0.001 | -7.7 | -10.1 | -5.2 | <0.001 |
|  | FAI | Age | 20-40 | 78.3 | -22.3 | 178.9 | 0.127 | 40.1 | -65.5 | 145.6 | 0.457 | 48.5 | -62.7 | 159.7 | 0.393 |
|  |  |  | 40-60 | 37.9 | -36.7 | 112.4 | 0.320 | 30.4 | -47.1 | 107.8 | 0.442 | 14.7 | -68.4 | 97.9 | 0.729 |
|  |  |  | >60 | 36.9 | -2.6 | 76.3 | 0.067 | 11.4 | -25.6 | 48.3 | 0.547 | 17.9 | -20.9 | 56.6 | 0.367 |
|  |  | BMI | <25 | 172.4 | 83.4 | 261.3 | <0.001 | 64.6 | -9.5 | 138.8 | 0.088 | 72.1 | -5.3 | 149.5 | 0.068 |
|  |  |  | 25-28 | 144.5 | 41.2 | 247.8 | <0.001 | -5.9 | -103.5 | 91.8 | 0.907 | -15.6 | -117.9 | 86.8 | 0.766 |
|  |  |  | >28 | 266.3 | 198.7 | 333.9 | <0.001 | 26.8 | -34.6 | 88.2 | 0.392 | 44.1 | -18.2 | 106.3 | 0.166 |
|  | TT/E2 | Age | 20-40 | -1.4 | -2.7 | 0.0 | 0.051 | -1.5 | -3.0 | 0.0 | 0.047 | 0.4 | -0.9 | 1.7 | 0.547 |
|  |  |  | 40-60 | 0.6 | -0.6 | 1.7 | 0.344 | 0.4 | -0.8 | 1.6 | 0.557 | -0.2 | -1.4 | 1.0 | 0.731 |
|  |  |  | >60 | 0.0 | -1.2 | 1.1 | 0.980 | -0.1 | -1.3 | 1.2 | 0.913 | 0.0 | -1.2 | 1.3 | 0.953 |
|  |  | BMI | <25 | 0.8 | -0.7 | 2.3 | 0.276 | 0.4 | -1.2 | 1.9 | 0.637 | 0.2 | -1.4 | 1.9 | 0.776 |
|  |  |  | 25-28 | 1.9 | 0.7 | 3.2 | 0.002 | 1.0 | -0.3 | 2.3 | 0.140 | 0.9 | -0.4 | 2.3 | 0.184 |
|  |  |  | >28 | 0.7 | -0.4 | 1.7 | 0.202 | -1.0 | -2.1 | 0.1 | 0.075 | -0.4 | -1.4 | 0.6 | 0.419 |

Model 1: Unadjusted model. Model 2: Adjusts for age, race, education, marital status, family income to poverty ratio. Model 3: Adjusts for age, race, education, marital status, family income to poverty ratio, body mass index (kg/m^2^), smoking and drinking. TT, Testosterone; E_2_: Estradiol; SHBG: sex hormone-binding globulin; FAI: free androgen index; LC, lymphocyte count; NC, neutrophil count; NLR, neutrophil-to-lymphocyte ratio; PLT, platelet count; PLR, platelet-to-lymphocyte ratio; PPN, the product of platelet count and neutrophil count; SII, systemic immune-inflammation index

**Supplementary Table S4** Subgroup analysis of the association of [Inflammation](https://www.frontiersin.org/journals/immunology/sections/inflammation)-related Index and sex hormones in Female

| Index | Outcome | Subgroup | | Model1 | | | | Model 2 | | | | Model3 | | | |
| --- | --- | --- | --- | --- | --- | --- | --- | --- | --- | --- | --- | --- | --- | --- | --- |
|  |  |  |  | β | 95%CI low | 95%CI upp | p-value | β | 95%CI low | 95%CI upp | p-value | β | 95%CI low | 95%CI upp | p-value |
| log2-SII | Testosterone | Age | 20-40 | 1.0 | -0.6 | 2.6 | 0.236 | 1.0 | -0.7 | 2.7 | 0.252 | -0.3 | -2.3 | 1.7 | 0.762 |
|  |  |  | 40-60 | -0.1 | -1.4 | 1.2 | 0.872 | -0.6 | -2.0 | 0.9 | 0.432 | -0.9 | -2.5 | 0.7 | 0.260 |
|  |  |  | >60 | -1.5 | -2.6 | -0.4 | 0.010 | -0.9 | -2.0 | 0.1 | 0.089 | -0.8 | -1.9 | 0.2 | 0.118 |
|  |  | BMI | <25 | 0.3 | -0.8 | 1.5 | 0.580 | 0.4 | -0.7 | 1.5 | 0.494 | 0.1 | -1.1 | 1.3 | 0.827 |
|  |  |  | 25-28 | -1.5 | -4.0 | 1.0 | 0.234 | -2.6 | -5.4 | 0.1 | 0.061 | -3.1 | -6.0 | -0.1 | 0.045 |
|  |  |  | >28 | -0.1 | -1.2 | 1.0 | 0.872 | 0.1 | -1.0 | 1.3 | 0.813 | -0.3 | -1.5 | 0.9 | 0.607 |
|  | Estradiol | Age | 20-40 | 13.7 | -5.0 | 32.3 | 0.152 | 19.3 | -1.2 | 39.7 | 0.065 | 10.8 | 1.4 | 20.3 | 0.025 |
|  |  |  | 40-60 | 24.3 | 10.8 | 37.8 | <0.001 | 23.5 | 8.5 | 38.5 | 0.002 | 9.3 | 3.4 | 15.3 | 0.002 |
|  |  |  | >60 | 0.0 | -0.9 | 0.9 | 0.988 | 0.3 | -0.5 | 1.1 | 0.398 | 0.1 | -0.7 | 1.0 | 0.749 |
|  |  | BMI | <25 | 5.9 | -2.4 | 14.3 | 0.163 | 10.1 | 1.5 | 18.7 | 0.021 | 6.8 | -0.4 | 13.9 | 0.065 |
|  |  |  | 25-28 | 3.6 | -4.1 | 11.3 | 0.361 | 2.2 | -5.0 | 9.4 | 0.551 | -0.5 | -7.9 | 6.9 | 0.892 |
|  |  |  | >28 | 15.7 | 5.2 | 26.1 | 0.003 | 14.8 | 3.1 | 26.4 | 0.013 | 11.7 | 6.9 | 16.4 | <0.001 |
|  | SHBG | Age | 20-40 | -5.2 | -9.5 | -1.0 | 0.017 | -4.6 | -9.1 | -0.2 | 0.041 | -2.4 | -7.0 | 2.3 | 0.316 |
|  |  |  | 40-60 | -4.2 | -7.1 | -1.3 | 0.005 | -5.3 | -8.4 | -2.1 | 0.001 | -3.3 | -6.3 | -0.3 | 0.031 |
|  |  |  | >60 | -4.0 | -6.5 | -1.5 | 0.002 | -5.2 | -7.9 | -2.6 | <0.001 | -3.8 | -6.4 | -1.2 | 0.005 |
|  |  | BMI | <25 | -2.6 | -6.3 | 1.2 | 0.182 | -3.5 | -7.5 | 0.5 | 0.086 | -3.0 | -7.2 | 1.3 | 0.168 |
|  |  |  | 25-28 | -0.7 | -5.1 | 3.7 | 0.749 | -2.5 | -7.1 | 2.1 | 0.281 | -3.2 | -8.1 | 1.7 | 0.205 |
|  |  |  | >28 | -4.6 | -6.8 | -2.4 | <0.001 | -4.1 | -6.6 | -1.7 | 0.001 | -3.9 | -6.3 | -1.5 | 0.001 |
|  | FAI | Age | 20-40 | 6.4 | 3.0 | 9.8 | <0.001 | 6.7 | 3.2 | 10.3 | <0.001 | 1.4 | -2.5 | 5.2 | 0.483 |
|  |  |  | 40-60 | 1.8 | -0.4 | 4.0 | 0.114 | 1.2 | -1.2 | 3.6 | 0.320 | -1.1 | -3.6 | 1.5 | 0.400 |
|  |  |  | >60 | 0.0 | -3.5 | 3.5 | 0.995 | 1.5 | -0.3 | 3.3 | 0.101 | 0.9 | -0.9 | 2.7 | 0.334 |
|  |  | BMI | <25 | 1.5 | -2.7 | 5.7 | 0.472 | 2.0 | 0.1 | 3.9 | 0.044 | 1.5 | -0.6 | 3.6 | 0.158 |
|  |  |  | 25-28 | -1.0 | -4.9 | 2.9 | 0.613 | -2.4 | -6.4 | 1.5 | 0.225 | -2.5 | -6.8 | 1.8 | 0.260 |
|  |  |  | >28 | 3.2 | 1.0 | 5.3 | 0.004 | 2.1 | -0.1 | 4.4 | 0.066 | 0.7 | -1.7 | 3.0 | 0.584 |
|  | TT/E2 | Age | 20-40 | 0.0 | -0.1 | 0.1 | 0.725 | 0.0 | -0.2 | 0.1 | 0.509 | -0.1 | -0.2 | 0.1 | 0.358 |
|  |  |  | 40-60 | -0.1 | -0.2 | 0.0 | 0.083 | -0.1 | -0.2 | 0.1 | 0.367 | 0.0 | -0.1 | 0.1 | 0.882 |
|  |  |  | >60 | 0.0 | -0.2 | 0.2 | 0.973 | 0.0 | -0.2 | 0.2 | 0.882 | 0.1 | -0.1 | 0.3 | 0.316 |
|  |  | BMI | <25 | -0.1 | -0.3 | 0.1 | 0.225 | -0.1 | -0.3 | 0.1 | 0.187 | -0.1 | -0.3 | 0.1 | 0.336 |
|  |  |  | 25-28 | -0.2 | -0.4 | 0.0 | 0.094 | -0.3 | -0.5 | 0.0 | 0.027 | -0.2 | -0.5 | 0.0 | 0.079 |
|  |  |  | >28 | 0.0 | -0.1 | 0.1 | 0.992 | 0.0 | -0.1 | 0.1 | 0.653 | 0.1 | 0.0 | 0.2 | 0.229 |
| log2-NLR | Testosterone | Age | 20-40 | 0.6 | -1.4 | 2.5 | 0.562 | 0.9 | -1.2 | 3.0 | 0.413 | -0.7 | -3.1 | 1.6 | 0.538 |
|  |  |  | 40-60 | 0.0 | -1.5 | 1.6 | 0.960 | -1.0 | -2.7 | 0.7 | 0.244 | -1.6 | -3.4 | 0.3 | 0.102 |
|  |  |  | >60 | -1.5 | -2.7 | -0.2 | 0.023 | -0.5 | -1.8 | 0.7 | 0.394 | -0.6 | -1.8 | 0.7 | 0.355 |
|  |  | BMI | <25 | -0.3 | -1.6 | 1.1 | 0.684 | 0.2 | -1.1 | 1.4 | 0.816 | -0.2 | -1.6 | 1.2 | 0.814 |
|  |  |  | 25-28 | -1.8 | -4.7 | 1.2 | 0.235 | -2.6 | -5.9 | 0.7 | 0.119 | -3.3 | -6.9 | 0.2 | 0.068 |
|  |  |  | >28 | -0.3 | -1.6 | 0.9 | 0.597 | 0.6 | -0.8 | 1.9 | 0.424 | -0.1 | -1.6 | 1.3 | 0.861 |
|  | Estradiol | Age | 20-40 | 29.9 | 7.5 | 52.3 | 0.009 | 39.0 | 14.2 | 63.8 | 0.002 | 20.6 | 9.4 | 31.8 | <0.001 |
|  |  |  | 40-60 | 31.5 | 15.5 | 47.5 | <0.001 | 34.1 | 16.2 | 52.0 | <0.001 | 11.7 | 4.7 | 18.8 | 0.001 |
|  |  |  | >60 | 0.3 | -0.7 | 1.2 | 0.624 | 0.8 | -0.2 | 1.7 | 0.101 | 0.5 | -0.5 | 1.5 | 0.319 |
|  |  | BMI | <25 | 7.1 | -2.5 | 16.7 | 0.149 | 16.9 | 6.8 | 26.9 | 0.001 | 13.1 | 4.8 | 21.4 | 0.002 |
|  |  |  | 25-28 | 2.4 | -6.7 | 11.5 | 0.604 | 7.3 | -1.3 | 15.9 | 0.094 | 4.0 | -4.8 | 12.7 | 0.375 |
|  |  |  | >28 | 15.6 | 3.2 | 28.0 | 0.014 | 24.3 | 10.3 | 38.3 | 0.001 | 15.3 | 9.6 | 21.0 | <0.001 |
|  | SHBG | Age | 20-40 | -2.1 | -7.2 | 3.0 | 0.414 | -1.9 | -7.2 | 3.6 | 0.502 | -3.0 | -8.5 | 2.6 | 0.291 |
|  |  |  | 40-60 | -1.3 | -4.7 | 2.2 | 0.482 | -3.2 | -6.9 | 0.7 | 0.104 | -2.7 | -6.3 | 0.9 | 0.144 |
|  |  |  | >60 | -0.1 | -3.0 | 2.8 | 0.943 | -3.4 | -6.6 | -0.3 | 0.033 | -1.6 | -4.7 | 1.5 | 0.312 |
|  |  | BMI | <25 | -0.5 | -4.9 | 3.9 | 0.819 | -1.6 | -6.3 | 3.2 | 0.520 | -1.7 | -6.7 | 3.2 | 0.494 |
|  |  |  | 25-28 | 2.5 | -2.7 | 7.7 | 0.340 | -0.7 | -6.2 | 4.8 | 0.806 | -1.2 | -7.1 | 4.7 | 0.691 |
|  |  |  | >28 | -2.3 | -4.9 | 0.3 | 0.083 | -2.4 | -5.3 | 0.5 | 0.109 | -3.1 | -5.9 | -0.2 | 0.035 |
|  | FAI | Age | 20-40 | 3.2 | -0.9 | 7.3 | 0.127 | 4.5 | 0.3 | 8.8 | 0.038 | 0.5 | -4.1 | 5.0 | 0.840 |
|  |  |  | 40-60 | 1.4 | -1.2 | 4.0 | 0.286 | 0.2 | -2.7 | 3.1 | 0.884 | -2.0 | -5.0 | 1.0 | 0.195 |
|  |  |  | >60 | -2.2 | -6.3 | 1.9 | 0.292 | 1.1 | -1.0 | 3.2 | 0.303 | 0.2 | -2.0 | 2.3 | 0.871 |
|  |  | BMI | <25 | -0.4 | -5.3 | 4.5 | 0.863 | 1.0 | -1.2 | 3.3 | 0.379 | 0.8 | -1.6 | 3.3 | 0.511 |
|  |  |  | 25-28 | -3.7 | -8.3 | 0.9 | 0.114 | -3.1 | -7.8 | 1.6 | 0.198 | -3.4 | -8.5 | 1.7 | 0.194 |
|  |  |  | >28 | 0.9 | -1.7 | 3.5 | 0.494 | 2.1 | -0.6 | 4.8 | 0.128 | 0.5 | -2.3 | 3.3 | 0.741 |
|  | TT/E2 | Age | 20-40 | 0.0 | -0.2 | 0.1 | 0.494 | -0.1 | -0.2 | 0.1 | 0.274 | -0.1 | -0.3 | 0.1 | 0.222 |
|  |  |  | 40-60 | 0.0 | -0.2 | 0.1 | 0.821 | 0.0 | -0.1 | 0.2 | 0.968 | 0.0 | -0.1 | 0.2 | 0.865 |
|  |  |  | >60 | 0.0 | -0.2 | 0.2 | 0.860 | 0.0 | -0.2 | 0.2 | 0.871 | 0.1 | -0.1 | 0.3 | 0.373 |
|  |  | BMI | <25 | 0.0 | -0.3 | 0.2 | 0.715 | -0.1 | -0.3 | 0.1 | 0.323 | -0.1 | -0.4 | 0.1 | 0.257 |
|  |  |  | 25-28 | -0.1 | -0.4 | 0.2 | 0.484 | -0.3 | -0.6 | -0.1 | 0.019 | -0.3 | -0.6 | 0.0 | 0.027 |
|  |  |  | >28 | 0.1 | 0.0 | 0.2 | 0.060 | 0.0 | -0.1 | 0.2 | 0.576 | 0.1 | 0.0 | 0.2 | 0.207 |
| log2-PLR | Testosterone | Age | 20-40 | 0.4 | -2.0 | 2.9 | 0.735 | 0.5 | -2.1 | 3.0 | 0.734 | 0.1 | -2.7 | 2.9 | 0.927 |
|  |  |  | 40-60 | -0.3 | -2.2 | 1.5 | 0.736 | -0.3 | -2.3 | 1.7 | 0.774 | -0.5 | -2.7 | 1.7 | 0.643 |
|  |  |  | >60 | -0.8 | -2.4 | 0.8 | 0.323 | -0.5 | -2.0 | 1.1 | 0.563 | -0.5 | -2.0 | 1.1 | 0.560 |
|  |  | BMI | <25 | -0.1 | -1.8 | 1.6 | 0.921 | 0.7 | -0.9 | 2.3 | 0.388 | 0.5 | -1.3 | 2.2 | 0.600 |
|  |  |  | 25-28 | -0.9 | -4.4 | 2.7 | 0.634 | -1.6 | -5.5 | 2.4 | 0.438 | -2.1 | -6.4 | 2.2 | 0.346 |
|  |  |  | >28 | -0.6 | -2.1 | 1.0 | 0.479 | -0.1 | -1.7 | 1.6 | 0.925 | -0.4 | -2.1 | 1.3 | 0.644 |
|  | Estradiol | Age | 20-40 | 0.2 | -28.1 | 28.4 | 0.991 | 3.1 | -27.5 | 33.6 | 0.843 | -5.3 | -18.7 | 8.1 | 0.437 |
|  |  |  | 40-60 | 16.1 | -3.1 | 35.3 | 0.100 | 9.5 | -11.2 | 30.2 | 0.368 | 3.7 | -4.5 | 11.9 | 0.382 |
|  |  |  | >60 | -0.4 | -1.6 | 0.8 | 0.527 | -0.4 | -1.6 | 0.8 | 0.491 | -0.1 | -1.3 | 1.2 | 0.931 |
|  |  | BMI | <25 | -6.8 | -18.9 | 5.2 | 0.268 | 2.2 | -10.1 | 14.5 | 0.727 | -0.8 | -11.1 | 9.5 | 0.875 |
|  |  |  | 25-28 | -10.9 | -21.8 | 0.1 | 0.051 | -8.6 | -19.0 | 1.8 | 0.106 | -12.0 | -22.6 | -1.3 | 0.028 |
|  |  |  | >28 | 7.4 | -7.9 | 22.7 | 0.345 | 8.2 | -8.6 | 24.9 | 0.339 | 6.5 | -0.2 | 13.3 | 0.058 |
|  | SHBG | Age | 20-40 | 0.0 | -6.4 | 6.5 | 0.994 | -0.4 | -7.1 | 6.3 | 0.905 | -0.1 | -6.7 | 6.5 | 0.977 |
|  |  |  | 40-60 | 6.1 | 1.9 | 10.3 | 0.004 | 5.4 | 1.0 | 9.8 | 0.016 | 2.7 | -1.5 | 6.9 | 0.208 |
|  |  |  | >60 | 5.1 | 1.5 | 8.7 | 0.006 | 3.5 | -0.4 | 7.3 | 0.077 | 1.3 | -2.5 | 5.1 | 0.501 |
|  |  | BMI | <25 | 2.1 | -3.4 | 7.6 | 0.449 | 1.0 | -4.8 | 6.8 | 0.731 | -1.1 | -7.2 | 5.1 | 0.733 |
|  |  |  | 25-28 | 4.1 | -2.2 | 10.4 | 0.198 | 0.7 | -5.9 | 7.4 | 0.830 | -0.6 | -7.8 | 6.5 | 0.865 |
|  |  |  | >28 | 0.0 | -3.2 | 3.3 | 0.978 | -0.1 | -3.5 | 3.4 | 0.975 | 0.1 | -3.3 | 3.4 | 0.977 |
|  | FAI | Age | 20-40 | -2.4 | -7.6 | 2.7 | 0.354 | -2.2 | -7.4 | 3.1 | 0.422 | -3.5 | -8.9 | 1.9 | 0.206 |
|  |  |  | 40-60 | -6.1 | -9.2 | -2.9 | 0.000 | -6.3 | -9.6 | -3.0 | 0.000 | -5.7 | -9.2 | -2.2 | 0.002 |
|  |  |  | >60 | -3.0 | -8.1 | 2.1 | 0.250 | -1.9 | -4.5 | 0.7 | 0.151 | -1.1 | -3.7 | 1.5 | 0.421 |
|  |  | BMI | <25 | -1.2 | -7.3 | 4.9 | 0.708 | 0.1 | -2.6 | 2.9 | 0.930 | 0.0 | -3.1 | 3.0 | 0.994 |
|  |  |  | 25-28 | -5.1 | -10.6 | 0.5 | 0.075 | -4.6 | -10.3 | 1.1 | 0.112 | -4.4 | -10.6 | 1.8 | 0.164 |
|  |  |  | >28 | -3.9 | -7.0 | -0.7 | 0.017 | -3.5 | -6.7 | -0.3 | 0.032 | -4.3 | -7.5 | -1.0 | 0.010 |
|  | TT/E2 | Age | 20-40 | -0.1 | -0.3 | 0.1 | 0.188 | -0.1 | -0.3 | 0.1 | 0.180 | -0.1 | -0.3 | 0.1 | 0.201 |
|  |  |  | 40-60 | 0.0 | -0.2 | 0.2 | 0.948 | 0.1 | -0.1 | 0.3 | 0.330 | 0.1 | -0.1 | 0.3 | 0.364 |
|  |  |  | >60 | 0.2 | -0.1 | 0.4 | 0.158 | 0.1 | -0.1 | 0.4 | 0.338 | 0.1 | -0.2 | 0.4 | 0.514 |
|  |  | BMI | <25 | 0.0 | -0.2 | 0.3 | 0.833 | -0.1 | -0.4 | 0.2 | 0.455 | -0.1 | -0.4 | 0.2 | 0.583 |
|  |  |  | 25-28 | -0.1 | -0.4 | 0.2 | 0.509 | -0.3 | -0.6 | 0.1 | 0.126 | -0.3 | -0.6 | 0.1 | 0.127 |
|  |  |  | >28 | 0.1 | -0.1 | 0.2 | 0.457 | 0.1 | -0.1 | 0.2 | 0.384 | 0.1 | -0.1 | 0.2 | 0.313 |
| log2-PPN | Testosterone | Age | 20-40 | 1.2 | -0.3 | 2.8 | 0.112 | 1.1 | -0.5 | 2.8 | 0.182 | -0.2 | -2.1 | 1.8 | 0.879 |
|  |  |  | 40-60 | -0.1 | -1.3 | 1.2 | 0.901 | -0.3 | -1.6 | 1.1 | 0.703 | -0.4 | -2.0 | 1.1 | 0.591 |
|  |  |  | >60 | -1.6 | -2.8 | -0.4 | 0.007 | -1.3 | -2.4 | -0.2 | 0.017 | -1.2 | -2.3 | -0.1 | 0.040 |
|  |  | BMI | <25 | 0.9 | -0.2 | 2.1 | 0.119 | 0.3 | -0.8 | 1.4 | 0.562 | 0.2 | -1.1 | 1.4 | 0.775 |
|  |  |  | 25-28 | -1.3 | -3.8 | 1.2 | 0.299 | -2.8 | -5.6 | 0.0 | 0.051 | -2.9 | -5.9 | 0.1 | 0.062 |
|  |  |  | >28 | 0.3 | -0.7 | 1.4 | 0.545 | -0.1 | -1.2 | 1.1 | 0.898 | -0.4 | -1.6 | 0.9 | 0.575 |
|  | Estradiol | Age | 20-40 | 6.0 | -11.9 | 23.8 | 0.513 | 10.0 | -9.6 | 29.6 | 0.317 | 9.6 | 0.2 | 18.9 | 0.046 |
|  |  |  | 40-60 | 17.2 | 4.0 | 30.4 | 0.011 | 17.2 | 2.5 | 31.9 | 0.022 | 8.4 | 2.4 | 14.4 | 0.006 |
|  |  |  | >60 | 0.0 | -0.9 | 0.9 | 0.963 | 0.4 | -0.5 | 1.2 | 0.411 | -0.1 | -1.0 | 0.8 | 0.883 |
|  |  | BMI | <25 | 10.4 | 1.9 | 19.0 | 0.017 | 7.2 | -1.6 | 16.0 | 0.110 | 4.6 | -2.9 | 12.0 | 0.228 |
|  |  |  | 25-28 | 10.8 | 3.2 | 18.5 | 0.006 | 3.5 | -3.9 | 10.9 | 0.353 | 1.9 | -5.6 | 9.5 | 0.612 |
|  |  |  | >28 | 16.6 | 6.2 | 27.0 | 0.002 | 8.9 | -2.9 | 20.6 | 0.139 | 9.6 | 4.7 | 14.4 | 0.000 |
|  | SHBG | Age | 20-40 | -8.2 | -12.2 | -4.1 | <0.001 | -7.2 | -11.5 | -3.0 | 0.001 | -2.6 | -7.2 | 2.1 | 0.278 |
|  |  |  | 40-60 | -9.8 | -12.6 | -7.0 | <0.001 | -10.5 | -13.5 | -7.5 | <0.001 | -6.1 | -9.1 | -3.1 | <0.001 |
|  |  |  | >60 | -11.8 | -14.4 | -9.2 | <0.001 | -10.6 | -13.3 | -7.9 | <0.001 | -7.9 | -10.6 | -5.1 | <0.001 |
|  |  | BMI | <25 | -6.0 | -9.8 | -2.2 | 0.002 | -6.6 | -10.7 | -2.5 | 0.002 | -4.4 | -8.8 | -0.1 | 0.047 |
|  |  |  | 25-28 | -5.3 | -9.7 | -0.9 | 0.018 | -5.2 | -9.8 | -0.5 | 0.031 | -5.4 | -10.4 | -0.4 | 0.034 |
|  |  |  | >28 | -7.5 | -9.7 | -5.3 | <0.001 | -6.7 | -9.1 | -4.2 | <0.001 | -5.8 | -8.2 | -3.4 | <0.001 |
|  | FAI | Age | 20-40 | 10.6 | 7.4 | 13.8 | <0.001 | 10.5 | 7.1 | 13.8 | <0.001 | 4.1 | 0.3 | 7.8 | 0.035 |
|  |  |  | 40-60 | 5.2 | 3.1 | 7.3 | <0.001 | 5.2 | 2.9 | 7.5 | <0.001 | 2.2 | -0.4 | 4.7 | 0.094 |
|  |  |  | >60 | 3.5 | -0.2 | 7.3 | 0.066 | 3.4 | 1.6 | 5.3 | <0.001 | 2.4 | 0.5 | 4.3 | 0.013 |
|  |  | BMI | <25 | 4.1 | -0.2 | 8.4 | 0.060 | 3.2 | 1.3 | 5.2 | 0.001 | 2.6 | 0.4 | 4.7 | 0.020 |
|  |  |  | 25-28 | 3.2 | -0.8 | 7.1 | 0.115 | -0.5 | -4.6 | 3.5 | 0.798 | -0.5 | -4.8 | 3.9 | 0.838 |
|  |  |  | >28 | 7.4 | 5.3 | 9.6 | <0.001 | 4.5 | 2.3 | 6.8 | <0.0001 | 3.3 | 0.9 | 5.6 | 0.007 |
|  | TT/E2 | Age | 20-40 | 0.0 | -0.1 | 0.1 | 0.481 | 0.0 | -0.1 | 0.1 | 0.646 | 0.0 | -0.1 | 0.1 | 0.929 |
|  |  |  | 40-60 | -0.2 | -0.3 | -0.1 | 0.002 | -0.2 | -0.3 | 0.0 | 0.014 | -0.1 | -0.2 | 0.1 | 0.275 |
|  |  |  | >60 | -0.1 | -0.3 | 0.1 | 0.200 | -0.1 | -0.3 | 0.1 | 0.388 | 0.1 | -0.1 | 0.3 | 0.399 |
|  |  | BMI | <25 | -0.2 | -0.4 | 0.0 | 0.021 | -0.1 | -0.3 | 0.1 | 0.195 | -0.1 | -0.3 | 0.2 | 0.557 |
|  |  |  | 25-28 | -0.3 | -0.5 | 0.0 | 0.022 | -0.2 | -0.4 | 0.1 | 0.152 | -0.1 | -0.3 | 0.2 | 0.539 |
|  |  |  | >28 | -0.1 | -0.2 | 0.0 | 0.038 | 0.0 | -0.1 | 0.1 | 0.860 | 0.0 | -0.1 | 0.1 | 0.522 |
| log2-LC | Testosterone | Age | 20-40 | 1.1 | -1.6 | 3.9 | 0.418 | 0.6 | -2.3 | 3.5 | 0.680 | 0.4 | -2.8 | 3.6 | 0.807 |
|  |  |  | 40-60 | 0.1 | -2.0 | 2.1 | 0.960 | 0.7 | -1.5 | 2.8 | 0.551 | 1.1 | -1.3 | 3.5 | 0.368 |
|  |  |  | >60 | 0.1 | -1.6 | 1.8 | 0.919 | -0.8 | -2.4 | 0.9 | 0.363 | -0.5 | -2.1 | 1.2 | 0.555 |
|  |  | BMI | <25 | 1.5 | -0.4 | 3.3 | 0.120 | -0.2 | -1.9 | 1.6 | 0.857 | 0.1 | -1.9 | 2.1 | 0.924 |
|  |  |  | 25-28 | 0.5 | -3.5 | 4.4 | 0.812 | -0.1 | -4.6 | 4.4 | 0.957 | 0.7 | -4.2 | 5.6 | 0.775 |
|  |  |  | >28 | 1.1 | -0.6 | 2.7 | 0.224 | -0.5 | -2.3 | 1.3 | 0.565 | -0.1 | -1.9 | 1.8 | 0.954 |
|  | Estradiol | Age | 20-40 | -20.5 | -52.2 | 11.2 | 0.205 | -24.1 | -58.7 | 10.5 | 0.173 | -2.8 | -18.3 | 12.7 | 0.724 |
|  |  |  | 40-60 | -14.9 | -35.9 | 6.1 | 0.165 | -12.5 | -35.2 | 10.3 | 0.285 | -2.3 | -11.5 | 6.8 | 0.617 |
|  |  |  | >60 | 0.0 | -1.3 | 1.3 | 0.965 | -0.1 | -1.3 | 1.2 | 0.926 | -0.5 | -1.8 | 0.8 | 0.474 |
|  |  | BMI | <25 | 9.9 | -3.4 | 23.1 | 0.145 | -8.4 | -22.1 | 5.4 | 0.235 | -6.6 | -18.2 | 5.0 | 0.268 |
|  |  |  | 25-28 | 17.8 | 5.8 | 29.9 | 0.004 | 3.1 | -8.8 | 15.0 | 0.608 | 6.4 | -5.7 | 18.4 | 0.301 |
|  |  |  | >28 | 2.6 | -13.9 | 19.2 | 0.757 | -15.1 | -33.6 | 3.5 | 0.111 | -5.7 | -13.1 | 1.8 | 0.134 |
|  | SHBG | Age | 20-40 | -10.7 | -18.0 | -3.5 | 0.004 | -9.2 | -16.7 | -1.7 | 0.017 | -0.6 | -8.2 | 7.0 | 0.878 |
|  |  |  | 40-60 | -15.2 | -19.7 | -10.7 | <0.001 | -13.6 | -18.4 | -8.9 | <0.001 | -6.7 | -11.3 | -2.0 | 0.005 |
|  |  |  | >60 | -15.0 | -18.8 | -11.2 | <0.001 | -10.9 | -15.1 | -6.8 | <0.001 | -8.0 | -12.0 | -3.9 | <0.001 |
|  |  | BMI | <25 | -8.1 | -14.1 | -2.1 | 0.008 | -7.5 | -13.9 | -1.0 | 0.023 | -3.2 | -10.1 | 3.7 | 0.365 |
|  |  |  | 25-28 | -11.3 | -18.2 | -4.4 | 0.001 | -6.5 | -14.0 | 1.1 | 0.092 | -5.5 | -13.5 | 2.6 | 0.183 |
|  |  |  | >28 | -7.4 | -10.9 | -3.9 | <0.001 | -6.2 | -10.0 | -2.3 | 0.002 | -4.3 | -7.9 | -0.6 | 0.023 |
|  | FAI | Age | 20-40 | 15.0 | 9.3 | 20.8 | <0.001 | 13.3 | 7.4 | 19.3 | <0.001 | 7.4 | 1.2 | 13.6 | 0.020 |
|  |  |  | 40-60 | 9.1 | 5.7 | 12.5 | <0.001 | 10.0 | 6.4 | 13.6 | <0.001 | 7.8 | 3.9 | 11.6 | <0.001 |
|  |  |  | >60 | 7.2 | 1.9 | 12.6 | 0.008 | 4.0 | 1.2 | 6.8 | 0.006 | 3.1 | 0.3 | 5.9 | 0.030 |
|  |  | BMI | <25 | 6.2 | -0.5 | 12.9 | 0.072 | 3.0 | 0.0 | 6.1 | 0.054 | 2.4 | -1.0 | 5.9 | 0.164 |
|  |  |  | 25-28 | 10.3 | 4.2 | 16.4 | 0.001 | 5.2 | -1.2 | 11.7 | 0.112 | 5.4 | -1.6 | 12.4 | 0.130 |
|  |  |  | >28 | 10.8 | 7.4 | 14.2 | <0.001 | 6.0 | 2.4 | 9.5 | 0.001 | 6.0 | 2.4 | 9.6 | 0.001 |
|  | TT/E2 | Age | 20-40 | 0.2 | 0.0 | 0.3 | 0.064 | 0.2 | 0.0 | 0.4 | 0.053 | 0.2 | 0.0 | 0.4 | 0.099 |
|  |  |  | 40-60 | -0.2 | -0.4 | 0.0 | 0.020 | -0.2 | -0.4 | -0.1 | 0.013 | -0.2 | -0.4 | 0.1 | 0.145 |
|  |  |  | >60 | -0.2 | -0.5 | 0.0 | 0.074 | -0.2 | -0.5 | 0.1 | 0.289 | -0.1 | -0.3 | 0.3 | 0.751 |
|  |  | BMI | <25 | -0.3 | -0.6 | 0.0 | 0.095 | 0.0 | -0.3 | 0.3 | 0.947 | 0.1 | -0.2 | 0.5 | 0.527 |
|  |  |  | 25-28 | -0.2 | -0.5 | 0.2 | 0.335 | 0.3 | -0.1 | 0.6 | 0.182 | 0.4 | 0.0 | 0.8 | 0.059 |
|  |  |  | >28 | -0.2 | -0.4 | -0.1 | 0.001 | -0.1 | -0.2 | 0.1 | 0.322 | -0.1 | -0.2 | 0.1 | 0.375 |
| log2-NC | Testosterone | Age | 20-40 | 1.2 | -0.8 | 3.2 | 0.229 | 1.3 | -0.9 | 3.5 | 0.241 | -0.6 | -3.2 | 1.9 | 0.631 |
|  |  |  | 40-60 | 0.1 | -1.6 | 1.7 | 0.925 | -0.7 | -2.5 | 1.1 | 0.455 | -1.1 | -3.2 | 1.0 | 0.293 |
|  |  |  | >60 | -2.3 | -3.9 | -0.7 | 0.005 | -1.5 | -3.0 | 0.0 | 0.057 | -1.4 | -3.0 | 0.2 | 0.083 |
|  |  | BMI | <25 | 0.7 | -0.9 | 2.2 | 0.407 | 0.1 | -1.4 | 1.6 | 0.909 | -0.2 | -1.8 | 1.5 | 0.847 |
|  |  |  | 25-28 | -1.9 | -5.2 | 1.4 | 0.258 | -3.6 | -7.3 | 0.2 | 0.065 | -4.0 | -8.1 | 0.2 | 0.060 |
|  |  |  | >28 | 0.3 | -1.1 | 1.8 | 0.664 | 0.3 | -1.2 | 1.9 | 0.676 | -0.2 | -1.9 | 1.5 | 0.796 |
|  | Estradiol | Age | 20-40 | 21.3 | -2.1 | 44.7 | 0.074 | 29.3 | 3.2 | 55.4 | 0.028 | 23.0 | 10.7 | 35.3 | <0.001 |
|  |  |  | 40-60 | 26.5 | 9.2 | 43.8 | 0.003 | 31.2 | 11.7 | 50.7 | 0.002 | 13.2 | 5.2 | 21.2 | 0.001 |
|  |  |  | >60 | 0.4 | -0.8 | 1.7 | 0.506 | 1.2 | 0.0 | 2.3 | 0.052 | 0.4 | -0.9 | 1.6 | 0.570 |
|  |  | BMI | <25 | 16.6 | 5.4 | 27.8 | 0.004 | 16.5 | 4.9 | 28.1 | 0.005 | 13.2 | 3.5 | 22.9 | 0.008 |
|  |  |  | 25-28 | 15.7 | 5.5 | 25.8 | 0.003 | 11.9 | 2.1 | 21.8 | 0.018 | 9.9 | -0.3 | 20.0 | 0.057 |
|  |  |  | >28 | 22.3 | 8.2 | 36.5 | 0.002 | 20.6 | 4.5 | 36.7 | 0.012 | 16.5 | 9.7 | 23.2 | <0.001 |
|  | SHBG | Age | 20-40 | -8.2 | -13.5 | -2.8 | 0.003 | -7.3 | -12.9 | -1.6 | 0.012 | -4.0 | -10.1 | 2.1 | 0.200 |
|  |  |  | 40-60 | -11.5 | -15.2 | -7.9 | <0.001 | -13.5 | -17.5 | -9.4 | <0.001 | -8.3 | -12.3 | -4.3 | <0.001 |
|  |  |  | >60 | -13.9 | -17.5 | -10.3 | <0.001 | -14.8 | -18.6 | -11.0 | <0.001 | -9.9 | -13.8 | -6.0 | <0.001 |
|  |  | BMI | <25 | -6.3 | -11.3 | -1.3 | 0.013 | -7.2 | -12.6 | -1.8 | 0.009 | -4.5 | -10.2 | 1.2 | 0.124 |
|  |  |  | 25-28 | -4.8 | -10.6 | 1.0 | 0.105 | -5.5 | -11.8 | 0.9 | 0.091 | -5.5 | -12.3 | 1.3 | 0.112 |
|  |  |  | >28 | -8.4 | -11.4 | -5.5 | <0.001 | -7.8 | -11.2 | -4.5 | <0.001 | -7.7 | -11.1 | -4.4 | <0.001 |
|  | FAI | Age | 20-40 | 11.6 | 7.4 | 15.9 | <0.001 | 12.6 | 8.1 | 17.0 | <0.001 | 5.3 | 0.3 | 10.3 | 0.037 |
|  |  |  | 40-60 | 7.7 | 4.9 | 10.4 | <0.001 | 7.4 | 4.4 | 10.5 | <0.001 | 3.3 | -0.1 | 6.7 | 0.058 |
|  |  |  | >60 | 3.1 | -2.1 | 8.3 | 0.238 | 5.2 | 2.6 | 7.8 | <0.001 | 3.2 | 0.5 | 5.9 | 0.022 |
|  |  | BMI | <25 | 3.7 | -1.9 | 9.3 | 0.193 | 3.4 | 0.9 | 6.0 | 0.009 | 2.8 | -0.1 | 5.6 | 0.056 |
|  |  |  | 25-28 | 2.6 | -2.6 | 7.8 | 0.326 | -0.4 | -5.9 | 5.0 | 0.873 | -0.7 | -6.6 | 5.3 | 0.819 |
|  |  |  | >28 | 9.1 | 6.1 | 12.0 | <0.001 | 7.3 | 4.2 | 10.4 | <0.001 | 5.6 | 2.3 | 8.9 | 0.001 |
|  | TT/E2 | Age | 20-40 | 0.0 | -0.1 | 0.2 | 0.517 | 0.0 | -0.1 | 0.2 | 0.763 | 0.0 | -0.2 | 0.2 | 0.984 |
|  |  |  | 40-60 | -0.2 | -0.3 | 0.0 | 0.032 | -0.2 | -0.3 | 0.0 | 0.038 | -0.1 | -0.3 | 0.1 | 0.286 |
|  |  |  | >60 | -0.2 | -0.4 | 0.1 | 0.136 | -0.2 | -0.4 | 0.1 | 0.233 | 0.1 | -0.2 | 0.4 | 0.413 |
|  |  | BMI | <25 | -0.2 | -0.5 | 0.0 | 0.070 | -0.1 | -0.4 | 0.1 | 0.284 | -0.1 | -0.4 | 0.2 | 0.434 |
|  |  |  | 25-28 | -0.2 | -0.5 | 0.1 | 0.110 | -0.3 | -0.6 | 0.1 | 0.113 | -0.2 | -0.5 | 0.2 | 0.330 |
|  |  |  | >28 | 0.0 | -0.2 | 0.1 | 0.501 | 0.0 | -0.2 | 0.1 | 0.826 | 0.1 | -0.1 | 0.2 | 0.498 |
| log2-PLT | Testosterone | Age | 20-40 | 2.4 | -0.9 | 5.6 | 0.156 | 1.6 | -1.8 | 5.1 | 0.348 | 0.8 | -3.0 | 4.6 | 0.678 |
|  |  |  | 40-60 | -0.6 | -3.3 | 2.1 | 0.672 | 0.5 | -2.3 | 3.4 | 0.709 | 0.8 | -2.4 | 4.0 | 0.611 |
|  |  |  | >60 | -1.6 | -3.9 | 0.8 | 0.189 | -2.3 | -4.5 | -0.1 | 0.040 | -1.8 | -4.0 | 0.4 | 0.105 |
|  |  | BMI | <25 | 2.4 | -0.1 | 4.8 | 0.055 | 1.2 | -1.1 | 3.4 | 0.315 | 1.1 | -1.4 | 3.6 | 0.379 |
|  |  |  | 25-28 | -1.0 | -6.1 | 4.2 | 0.707 | -3.4 | -9.0 | 2.3 | 0.243 | -3.0 | -9.0 | 3.1 | 0.338 |
|  |  |  | >28 | 0.6 | -1.5 | 2.8 | 0.563 | -1.0 | -3.3 | 1.3 | 0.388 | -0.9 | -3.2 | 1.5 | 0.469 |
|  | Estradiol | Age | 20-40 | -28.8 | -66.6 | 9.0 | 0.135 | -27.9 | -68.6 | 12.9 | 0.180 | -13.9 | -32.2 | 4.5 | 0.138 |
|  |  |  | 40-60 | 8.0 | -20.2 | 36.1 | 0.580 | -1.7 | -31.9 | 28.6 | 0.914 | 3.8 | -8.2 | 15.9 | 0.533 |
|  |  |  | >60 | -0.8 | -2.6 | 1.0 | 0.384 | -1.0 | -2.6 | 0.7 | 0.263 | -1.0 | -2.7 | 0.8 | 0.284 |
|  |  | BMI | <25 | 2.8 | -14.8 | 20.5 | 0.755 | -9.2 | -26.9 | 8.5 | 0.308 | -12.6 | -27.5 | 2.3 | 0.099 |
|  |  |  | 25-28 | 7.8 | -8.0 | 23.6 | 0.331 | -12.5 | -27.3 | 2.3 | 0.098 | -13.5 | -28.3 | 1.4 | 0.075 |
|  |  |  | >28 | 18.4 | -2.8 | 39.6 | 0.089 | -8.2 | -31.7 | 15.4 | 0.496 | 3.7 | -5.8 | 13.2 | 0.444 |
|  | SHBG | Age | 20-40 | -15.2 | -23.8 | -6.6 | 0.001 | -13.4 | -22.3 | -4.6 | 0.003 | -1.0 | -10.1 | 8.0 | 0.824 |
|  |  |  | 40-60 | -14.3 | -20.4 | -8.3 | <0.001 | -12.5 | -18.9 | -6.2 | 0.000 | -5.8 | -11.9 | 0.3 | 0.062 |
|  |  |  | >60 | -17.7 | -22.9 | -12.4 | <0.001 | -12.1 | -17.6 | -6.5 | <0.001 | -11.3 | -16.7 | -5.9 | <0.001 |
|  |  | BMI | <25 | -9.8 | -17.8 | -1.9 | 0.016 | -10.2 | -18.5 | -2.0 | 0.016 | -7.5 | -16.3 | 1.4 | 0.098 |
|  |  |  | 25-28 | -10.7 | -19.8 | -1.7 | 0.020 | -8.6 | -18.0 | 0.8 | 0.073 | -9.5 | -19.4 | 0.4 | 0.060 |
|  |  |  | >28 | -12.0 | -16.5 | -7.6 | <0.001 | -10.1 | -14.9 | -5.2 | <0.001 | -6.8 | -11.5 | -2.1 | 0.004 |
|  | FAI | Age | 20-40 | 17.0 | 10.1 | 23.8 | <0.001 | 14.6 | 7.6 | 21.6 | <0.001 | 3.9 | -3.5 | 11.3 | 0.303 |
|  |  |  | 40-60 | 3.4 | -1.3 | 8.0 | 0.153 | 4.3 | -0.5 | 9.2 | 0.079 | 1.3 | -3.8 | 6.5 | 0.613 |
|  |  |  | >60 | 7.4 | 0.0 | 14.9 | 0.051 | 3.1 | -0.7 | 6.8 | 0.107 | 3.3 | -0.5 | 7.0 | 0.086 |
|  |  | BMI | <25 | 8.4 | -0.5 | 17.3 | 0.065 | 5.2 | 1.3 | 9.2 | 0.009 | 4.0 | -0.4 | 8.4 | 0.076 |
|  |  |  | 25-28 | 7.0 | -1.0 | 15.1 | 0.087 | -1.1 | -9.2 | 6.9 | 0.783 | -0.3 | -9.0 | 8.4 | 0.944 |
|  |  |  | >28 | 10.3 | 5.9 | 14.7 | <0.001 | 2.6 | -1.9 | 7.2 | 0.254 | 1.3 | -3.4 | 5.9 | 0.594 |
|  | TT/E2 | Age | 20-40 | 0.1 | -0.2 | 0.3 | 0.657 | 0.1 | -0.2 | 0.3 | 0.629 | 0.0 | -0.2 | 0.3 | 0.838 |
|  |  |  | 40-60 | -0.4 | -0.6 | -0.2 | 0.001 | -0.2 | -0.5 | 0.0 | 0.059 | -0.1 | -0.4 | 0.2 | 0.554 |
|  |  |  | >60 | -0.1 | -0.4 | 0.3 | 0.692 | 0.0 | -0.4 | 0.4 | 0.982 | 0.1 | -0.3 | 0.5 | 0.603 |
|  |  | BMI | <25 | -0.4 | -0.8 | 0.0 | 0.056 | -0.2 | -0.6 | 0.2 | 0.325 | 0.0 | -0.4 | 0.5 | 0.985 |
|  |  |  | 25-28 | -0.5 | -1.0 | -0.1 | 0.027 | -0.1 | -0.6 | 0.4 | 0.615 | 0.1 | -0.4 | 0.6 | 0.838 |
|  |  |  | >28 | -0.3 | -0.5 | -0.1 | 0.001 | 0.0 | -0.2 | 0.2 | 0.975 | 0.0 | -0.2 | 0.2 | 0.770 |

Model 1: Unadjusted model. Model 2: Adjusts for age, race, education, marital status, family income to poverty ratio. Model 3: Adjusts for age, race, education, marital status, family income to poverty ratio, body mass index (kg/m^2^), smoking and drinking. TT, Testosterone; E2: Estradiol; SHBG: sex hormone-binding globulin; FAI: free androgen index; LC, lymphocyte count; NC, neutrophil count; NLR, neutrophil-to-lymphocyte ratio; PLT, platelet count; PLR, platelet-to-lymphocyte ratio; PPN, the product of platelet count and neutrophil count; SII, systemic immune-inflammation index

**Supplementary Table S5.** Sensitivity analysis in Male

|  | Outcome | Variable | Model1 | | | | Model 2 | | | | Model3 | | | |
| --- | --- | --- | --- | --- | --- | --- | --- | --- | --- | --- | --- | --- | --- | --- |
|  |  |  | β | 95%CI low | 95%CI upp | p-value | β | 95%CI low | 95%CI upp | p-value | β | 95%CI low | 95%CI upp | p-value |
| SII | Testosterone | Q1 | Reference | | | | Reference | | | | Reference | | | |
|  |  | Q2 | -24.8 | -40.1 | -9.5 | 0.002 | -26.4 | -42.1 | -10.7 | 0.001 | -17.4 | -32.6 | -2.3 | 0.024 |
|  |  | Q3 | -36.5 | -51.8 | -21.2 | <0.001 | -34.5 | -50.4 | -18.6 | <0.001 | -20.6 | -35.9 | -5.3 | 0.008 |
|  |  | Q4 | -54.9 | -70.2 | -39.6 | <0.001 | -47.3 | -63.3 | -31.2 | <0.001 | -29.9 | -45.4 | -14.3 | <0.001 |
|  |  | P for trend | <0.001 | | | | <0.001 | | | | <0.001 | | | |
|  | Estradiol | Q1 | Reference | | | | Reference | | | | Reference | | | |
|  |  | Q2 | -0.8 | -1.6 | 0.0 | 0.064 | -0.3 | -1.2 | 0.5 | 0.459 | -0.4 | -1.3 | 0.4 | 0.322 |
|  |  | Q3 | -0.9 | -1.7 | -0.1 | 0.034 | -0.4 | -1.2 | 0.5 | 0.431 | -0.5 | -1.4 | 0.4 | 0.268 |
|  |  | Q4 | -1.2 | -2.0 | -0.4 | 0.004 | -0.7 | -1.5 | 0.2 | 0.131 | -0.8 | -1.7 | 0.1 | 0.076 |
|  |  | P for trend | 0.004 | | | | 0.149 | | | | 0.086 | | | |
|  | SHBG | Q1 | Reference | | | | Reference | | | | Reference | | | |
|  |  | Q2 | -3.5 | -5.6 | -1.3 | 0.001 | -4.2 | -6.2 | -2.3 | <0.001 | -3.3 | -5.2 | -1.3 | 0.001 |
|  |  | Q3 | -3.5 | -5.7 | -1.4 | 0.001 | -4.1 | -6.1 | -2.2 | <0.001 | -2.6 | -4.5 | -0.6 | 0.009 |
|  |  | Q4 | -1.5 | -3.6 | 0.7 | 0.184 | -5.6 | -7.6 | -3.6 | <0.001 | -3.7 | -5.7 | -1.7 | <0.001 |
|  |  | P for trend | 0.202 | | | | <0.001 | | | | 0.001 | | | |
|  | FAI | Q1 | Reference | | | | Reference | | | | Reference | | | |
|  |  | Q2 | 34.3 | -14.8 | 83.5 | 0.171 | 46.0 | 3.1 | 88.9 | 0.036 | 51.5 | 6.3 | 96.8 | 0.026 |
|  |  | Q3 | 1.1 | -48.0 | 50.3 | 0.964 | 11.6 | -31.9 | 55.0 | 0.602 | 12.5 | -33.3 | 58.3 | 0.593 |
|  |  | Q4 | -81.8 | -130.9 | -32.6 | 0.001 | 19.9 | -24.0 | 63.8 | 0.374 | 24.0 | -22.5 | 70.6 | 0.311 |
|  |  | P for trend | <0.001 | | | | 0.743 | | | | 0.674 | | | |
|  | TT/E2 | Q1 | Reference | | | | Reference | | | | Reference | | | |
|  |  | Q2 | 0.1 | -0.7 | 0.8 | 0.894 | 0.0 | -0.8 | 0.7 | 0.964 | 0.0 | -0.7 | 0.7 | 0.941 |
|  |  | Q3 | -0.1 | -0.8 | 0.6 | 0.804 | -0.2 | -1.0 | 0.5 | 0.566 | 0.1 | -0.6 | 0.9 | 0.707 |
|  |  | Q4 | -0.8 | -1.6 | -0.1 | 0.027 | -0.4 | -1.2 | 0.4 | 0.303 | 0.2 | -0.6 | 0.9 | 0.673 |
|  |  | P for trend | 0.027 | | | | 0.252 | | | | 0.587 | | | |
| NLR | Testosterone | Q1 | Reference | | | | Reference | | | | Reference | | | |
|  |  | Q2 | -25.0 | -40.3 | -9.6 | 0.001 | -21.4 | -37.2 | -5.5 | 0.008 | -10.2 | -25.4 | 5.1 | 0.192 |
|  |  | Q3 | -33.4 | -48.7 | -18.0 | <0.001 | -28.5 | -44.5 | -12.6 | 0.001 | -11.8 | -27.3 | 3.7 | 0.136 |
|  |  | Q4 | -45.7 | -61.1 | -30.4 | <0.001 | -30.8 | -47.3 | -14.3 | <0.001 | -16.1 | -32.1 | -0.1 | 0.048 |
|  |  | P for trend | <0.001 | | | | <0.001 | | | | 0.054 | | | |
|  | Estradiol | Q1 | Reference | | | | Reference | | | | Reference | | | |
|  |  | Q2 | -0.6 | -1.4 | 0.2 | 0.142 | -0.1 | -0.9 | 0.8 | 0.899 | 0.0 | -0.8 | 0.9 | 0.940 |
|  |  | Q3 | -0.2 | -1.0 | 0.6 | 0.585 | 0.4 | -0.4 | 1.3 | 0.332 | 0.2 | -0.7 | 1.1 | 0.594 |
|  |  | Q4 | -0.5 | -1.3 | 0.3 | 0.208 | 0.0 | -0.9 | 0.9 | 0.967 | 0.1 | -0.9 | 1.0 | 0.922 |
|  |  | P for trend | 0.368 | | | | 0.747 | | |  | 0.81 | | | |
|  | SHBG | Q1 | Reference | | | | Reference | | | | Reference | | | |
|  |  | Q2 | -2.6 | -4.7 | -0.5 | 0.018 | -3.5 | -5.5 | -1.6 | <0.001 | -2.2 | -4.1 | -0.2 | 0.030 |
|  |  | Q3 | -2.4 | -4.5 | -0.3 | 0.028 | -5.1 | -7.0 | -3.1 | <0.001 | -3.1 | -5.0 | -1.1 | 0.002 |
|  |  | Q4 | 5.0 | 2.9 | 7.1 | <0.001 | -2.2 | -4.2 | -0.1 | 0.036 | -0.7 | -2.7 | 1.4 | 0.531 |
|  |  | P for trend | <0.001 | | | | 0.012 | | |  | 0.378 | | | |
|  | FAI | Q1 | Reference | | | | Reference | | | | Reference | | | |
|  |  | Q2 | -3.0 | -51.9 | 45.9 | 0.904 | 19.7 | -23.4 | 62.9 | 0.371 | 20.1 | -25.3 | 65.6 | 0.385 |
|  |  | Q3 | -19.4 | -68.2 | 29.5 | 0.438 | 51.2 | 7.8 | 94.7 | 0.021 | 52.8 | 6.6 | 99.0 | 0.025 |
|  |  | Q4 | -192.7 | -241.6 | -143.8 | <0.001 | -3.2 | -48.3 | 41.9 | 0.889 | 0.1 | -47.6 | 47.8 | 0.997 |
|  |  | P for trend | <0.001 | | | | 0.73 | | |  | 0.65 | | | |
|  | TT/E2 | Q1 | Reference | | | | Reference | | | | Reference | | | |
|  |  | Q2 | 0.0 | -0.8 | 0.7 | 0.931 | -0.1 | -0.9 | 0.7 | 0.785 | 0.2 | -0.5 | 1.0 | 0.518 |
|  |  | Q3 | 0.1 | -0.6 | 0.8 | 0.774 | 0.3 | -0.4 | 1.1 | 0.397 | 0.7 | 0.0 | 1.4 | 0.055 |
|  |  | Q4 | -1.1 | -1.8 | -0.4 | 0.004 | 0.0 | -0.8 | 0.8 | 0.977 | 0.5 | -0.3 | 1.2 | 0.210 |
|  |  | P for trend | 0.008 | | | | 0.734 | | |  | 0.11 | | | |
| PLR | Testosterone | Q1 | Reference | | | | Reference | | | | Reference | | | |
|  |  | Q2 | 6.0 | -9.4 | 21.4 | 0.443 | 8.7 | -6.9 | 24.3 | 0.275 | 6.4 | -8.7 | 21.5 | 0.405 |
|  |  | Q3 | 11.7 | -3.7 | 27.1 | 0.136 | 19.3 | 3.6 | 35.0 | 0.016 | 13.7 | -1.4 | 28.8 | 0.075 |
|  |  | Q4 | 19.6 | 4.2 | 35.0 | 0.013 | 27.8 | 12.0 | 43.6 | 0.001 | 18.1 | 2.9 | 33.4 | 0.020 |
|  |  | P for trend | 0.009 | | | | <0.001 | | | | 0.012 | | | |
|  | Estradiol | Q1 | Reference | | | | Reference | | | | Reference | | | |
|  |  | Q2 | -0.6 | -1.4 | 0.2 | 0.155 | -0.3 | -1.2 | 0.5 | 0.425 | -0.1 | -1.0 | 0.7 | 0.770 |
|  |  | Q3 | -0.8 | -1.6 | 0.0 | 0.046 | -0.7 | -1.5 | 0.2 | 0.117 | -0.3 | -1.2 | 0.6 | 0.480 |
|  |  | Q4 | -1.1 | -1.9 | -0.2 | 0.011 | -1.1 | -2.0 | -0.3 | 0.011 | -0.5 | -1.4 | 0.4 | 0.244 |
|  |  | P for trend | 0.01 | | | | 0.008 | | | | 0.218 | | | |
|  | SHBG | Q1 | Reference | | | | Reference | | | | Reference | | | |
|  |  | Q2 | -0.8 | -2.9 | 1.3 | 0.460 | 0.2 | -1.7 | 2.1 | 0.844 | -0.1 | -2.0 | 1.8 | 0.927 |
|  |  | Q3 | 0.7 | -1.4 | 2.8 | 0.514 | 0.8 | -1.1 | 2.8 | 0.403 | 0.4 | -1.5 | 2.4 | 0.661 |
|  |  | Q4 | 4.5 | 2.4 | 6.6 | <0.001 | 1.2 | -0.7 | 3.2 | 0.214 | 0.0 | -2.0 | 1.9 | 0.996 |
|  |  | P for trend | <0.001 | | | | 0.168 | | | | 0.869 | | | |
|  | FAI | Q1 | Reference | | | | Reference | | | | Reference | | | |
|  |  | Q2 | 46.1 | -3.1 | 95.3 | 0.067 | 28.8 | -13.8 | 71.4 | 0.185 | 28.8 | -16.2 | 73.8 | 0.209 |
|  |  | Q3 | 19.4 | -29.8 | 68.7 | 0.439 | 31.8 | -11.0 | 74.5 | 0.145 | 28.0 | -17.0 | 73.0 | 0.223 |
|  |  | Q4 | -52.3 | -101.5 | -3.1 | 0.038 | 28.7 | -14.4 | 71.7 | 0.192 | 28.6 | -17.0 | 74.2 | 0.219 |
|  |  | P for trend | 0.021 | | | | 0.197 | | | | 0.243 | | | |
|  | TT/E2 | Q1 | Reference | | | | Reference | | | | Reference | | | |
|  |  | Q2 | 1.0 | 0.3 | 1.7 | 0.007 | 1.1 | 0.3 | 1.8 | 0.005 | 0.9 | 0.2 | 1.6 | 0.017 |
|  |  | Q3 | 1.1 | 0.4 | 1.8 | 0.004 | 1.4 | 0.7 | 2.2 | <0.001 | 1.3 | 0.5 | 2.0 | 0.001 |
|  |  | Q4 | 0.6 | -0.1 | 1.4 | 0.091 | 1.3 | 0.6 | 2.1 | 0.001 | 1.1 | 0.4 | 1.8 | 0.002 |
|  |  | P for trend | 0.097 | | | | <0.001 | | | | 0.001 | | | |
| PPN | Testosterone | Q1 | Reference | | | | Reference | | | | Reference | | | |
|  |  | Q2 | -22.7 | -37.9 | -7.5 | 0.004 | -23.5 | -39.1 | -7.8 | 0.003 | -16.9 | -32.1 | -1.8 | 0.028 |
|  |  | Q3 | -49.4 | -64.6 | -34.2 | <0.001 | -46.8 | -62.6 | -31.1 | <0.001 | -31.9 | -47.3 | -16.5 | <0.001 |
|  |  | Q4 | -75.1 | -90.3 | -59.9 | <0.001 | -79.1 | -94.9 | -63.2 | <0.001 | -54.9 | -70.6 | -39.3 | <0.001 |
|  |  | P for trend | <0.001 | | | | <0.001 | | | | <0.001 | | | |
|  | Estradiol | Q1 | Reference | | | | Reference | | | | Reference | | | |
|  |  | Q2 | -0.8 | -1.6 | 0.0 | 0.061 | -0.1 | -1.0 | 0.7 | 0.787 | -0.3 | -1.2 | 0.6 | 0.482 |
|  |  | Q3 | -0.9 | -1.7 | -0.1 | 0.037 | -0.1 | -1.0 | 0.7 | 0.789 | -0.7 | -1.6 | 0.2 | 0.147 |
|  |  | Q4 | -1.3 | -2.1 | -0.5 | 0.002 | -0.6 | -1.4 | 0.3 | 0.209 | -1.1 | -2.0 | -0.2 | 0.015 |
|  |  | P for trend | 0.002 | | | | 0.23 | | | | 0.011 | | | |
|  | SHBG | Q1 | Reference | | | | Reference | | | | Reference | | | |
|  |  | Q2 | -5.6 | -7.7 | -3.4 | <0.001 | -4.9 | -6.9 | -3.0 | <0.001 | -4.4 | -6.3 | -2.4 | <0.001 |
|  |  | Q3 | -8.9 | -11.0 | -6.8 | <0.001 | -6.7 | -8.7 | -4.8 | <0.001 | -5.0 | -7.0 | -3.0 | <0.001 |
|  |  | Q4 | -10.0 | -12.1 | -7.9 | <0.001 | -8.5 | -10.5 | -6.6 | <0.001 | -5.9 | -7.9 | -3.9 | <0.001 |
|  |  | P for trend | <0.001 | | | | <0.001 | | | | <0.001 | | | |
|  | FAI | Q1 | Reference | | | | Reference | | | | Reference | | | |
|  |  | Q2 | 77.3 | 28.1 | 126.6 | 0.002 | 59.0 | 15.9 | 102.0 | 0.007 | 62.7 | 17.3 | 108.1 | 0.007 |
|  |  | Q3 | 70.0 | 20.8 | 119.3 | 0.005 | 20.3 | -23.2 | 63.8 | 0.362 | 23.8 | -22.4 | 70.0 | 0.313 |
|  |  | Q4 | 63.0 | 13.7 | 112.2 | 0.012 | 17.4 | -26.3 | 61.0 | 0.436 | 20.8 | -26.2 | 67.8 | 0.386 |
|  |  | P for trend | 0.022 | | | | 0.881 | | | | 0.781 | | | |
|  | TT/E2 | Q1 | Reference | | | | Reference | | | | Reference | | | |
|  |  | Q2 | -0.2 | -0.9 | 0.6 | 0.682 | -0.5 | -1.2 | 0.3 | 0.203 | -0.1 | -0.8 | 0.6 | 0.767 |
|  |  | Q3 | -0.2 | -0.9 | 0.5 | 0.559 | -0.9 | -1.7 | -0.2 | 0.019 | -0.3 | -1.0 | 0.5 | 0.446 |
|  |  | Q4 | -0.9 | -1.7 | -0.2 | 0.013 | -1.6 | -2.4 | -0.9 | <0.001 | -0.7 | -1.5 | 0.0 | 0.062 |
|  |  | P for trend | 0.016 | | | | <0.001 | | | | 0.055 | | | |
| LC | Testosterone | Q1 | Reference | | | | Reference | | | | Reference | | | |
|  |  | Q2 | 3.4 | -12.8 | 19.7 | 0.678 | -10.9 | -27.7 | 5.9 | 0.204 | -13.7 | -30.0 | 2.5 | 0.097 |
|  |  | Q3 | -5.8 | -21.4 | 9.8 | 0.465 | -21.5 | -37.8 | -5.1 | 0.010 | -14.7 | -30.6 | 1.1 | 0.068 |
|  |  | Q4 | -33.9 | -49.7 | -18.2 | <0.001 | -54.5 | -71.2 | -37.8 | <0.001 | -37.5 | -53.7 | -21.3 | <0.001 |
|  |  | P for trend | <0.001 | | | | <0.001 | | | | <0.001 | | | |
|  | Estradiol | Q1 | Reference | | | | Reference | | | | Reference | | | |
|  |  | Q2 | -0.4 | -1.2 | 0.5 | 0.431 | -0.1 | -1.0 | 0.8 | 0.850 | -0.6 | -1.6 | 0.3 | 0.198 |
|  |  | Q3 | 0.0 | -0.8 | 0.9 | 0.919 | 0.3 | -0.5 | 1.2 | 0.447 | -0.1 | -1.0 | 0.8 | 0.793 |
|  |  | Q4 | 0.1 | -0.7 | 0.9 | 0.818 | 0.4 | -0.6 | 1.3 | 0.441 | -0.5 | -1.4 | 0.5 | 0.309 |
|  |  | P for trend | 0.568 | | | | 0.286 | | | | 0.564 | | | |
|  | SHBG | Q1 | Reference | | | | Reference | | | | Reference | | | |
|  |  | Q2 | -9.0 | -11.2 | -6.8 | <0.001 | -3.5 | -5.6 | -1.5 | 0.001 | -3.6 | -5.7 | -1.5 | 0.001 |
|  |  | Q3 | -11.4 | -13.6 | -9.3 | <0.001 | -4.6 | -6.6 | -2.6 | <0.001 | -3.8 | -5.8 | -1.8 | <0.001 |
|  |  | Q4 | -13.9 | -16.1 | -11.8 | <0.001 | -5.7 | -7.8 | -3.7 | <0.001 | -3.8 | -5.9 | -1.8 | <0.001 |
|  |  | P for trend | <0.001 | | | | <0.001 | | | | 0.001 | | | |
|  | FAI | Q1 | Reference | | | | Reference | | | | Reference | | | |
|  |  | Q2 | 172.3 | 120.8 | 223.8 | <0.001 | 12.1 | -33.9 | 58.1 | 0.607 | 6.1 | -42.4 | 54.7 | 0.805 |
|  |  | Q3 | 239.2 | 189.6 | 288.7 | <0.001 | 48.0 | 3.3 | 92.7 | 0.036 | 47.5 | 0.2 | 94.9 | 0.049 |
|  |  | Q4 | 211.1 | 160.9 | 261.3 | <0.001 | -16.3 | -62.0 | 29.4 | 0.485 | -16.8 | -65.2 | 31.7 | 0.498 |
|  |  | P for trend | <0.001 | | | | 0.726 | | | | 0.793 | | | |
|  | TT/E2 | Q1 | Reference | | | | Reference | | | | Reference | | | |
|  |  | Q2 | 0.5 | -0.3 | 1.3 | 0.212 | -0.7 | -1.5 | 0.1 | 0.083 | -0.4 | -1.2 | 0.4 | 0.303 |
|  |  | Q3 | 0.9 | 0.1 | 1.6 | 0.021 | -0.6 | -1.4 | 0.2 | 0.129 | -0.2 | -1.0 | 0.5 | 0.570 |
|  |  | Q4 | -0.1 | -0.9 | 0.6 | 0.743 | -1.9 | -2.7 | -1.1 | <0.001 | -1.2 | -2.0 | -0.5 | 0.002 |
|  |  | P for trend | 0.883 | | | | <0.001 | | | | 0.003 | | | |
| NC | Testosterone | Q1 | Reference | | | | Reference | | | | Reference | | | |
|  |  | Q2 | -47.1 | -62.3 | -31.8 | <0.001 | -41.2 | -57.0 | -25.4 | <0.001 | -28.8 | -44.1 | -13.5 | <0.001 |
|  |  | Q3 | -63.6 | -79.2 | -47.9 | <0.001 | -53.4 | -69.7 | -37.2 | <0.001 | -33.8 | -49.8 | -17.8 | <0.001 |
|  |  | Q4 | -91.8 | -107.1 | -76.5 | <0.001 | -91.0 | -107.2 | -74.9 | <0.001 | -60.7 | -76.8 | -44.7 | <0.001 |
|  |  | P for trend | <0.001 | | | | <0.001 | | | | <0.001 | | | |
|  | Estradiol | Q1 | Reference | | | | Reference | | | | Reference | | | |
|  |  | Q2 | -1.2 | -2.0 | -0.4 | 0.004 | -0.5 | -1.4 | 0.3 | 0.219 | -0.9 | -1.8 | 0.0 | 0.055 |
|  |  | Q3 | -0.6 | -1.4 | 0.3 | 0.184 | 0.3 | -0.6 | 1.2 | 0.453 | -0.2 | -1.1 | 0.7 | 0.654 |
|  |  | Q4 | -1.0 | -1.8 | -0.2 | 0.019 | -0.2 | -1.1 | 0.7 | 0.661 | -1.0 | -1.9 | 0.0 | 0.046 |
|  |  | P for trend | 0.098 | | | | 0.815 | | | | 0.163 | | | |
|  | SHBG | Q1 | Reference | | | | Reference | | | | Reference | | | |
|  |  | Q2 | -5.3 | -7.4 | -3.1 | <0.001 | -6.3 | -8.2 | -4.3 | <0.001 | -4.7 | -6.7 | -2.8 | <0.001 |
|  |  | Q3 | -5.6 | -7.8 | -3.4 | <0.001 | -7.2 | -9.2 | -5.2 | <0.001 | -5.2 | -7.2 | -3.2 | <0.001 |
|  |  | Q4 | -7.5 | -9.7 | -5.4 | <0.001 | -9.6 | -11.6 | -7.6 | <0.001 | -6.2 | -8.2 | -4.1 | <0.001 |
|  |  | P for trend | <0.001 | | | | <0.001 | | | | <0.001 | | | |
|  | FAI | Q1 | Reference | | | | Reference | | | | Reference | | | |
|  |  | Q2 | 9.7 | -39.9 | 59.3 | 0.702 | 41.2 | -2.4 | 84.8 | 0.064 | 35.4 | -10.7 | 81.4 | 0.132 |
|  |  | Q3 | -31.3 | -82.1 | 19.6 | 0.229 | 32.0 | -13.0 | 77.0 | 0.163 | 36.4 | -11.6 | 84.4 | 0.137 |
|  |  | Q4 | -42.9 | -92.6 | 6.7 | 0.090 | 1.0 | -43.6 | 45.6 | 0.965 | 1.7 | -46.6 | 49.9 | 0.947 |
|  |  | P for trend | 0.03 | | | | 0.793 | | | | 0.936 | | | |
|  | TT/E2 | Q1 | Reference | | | | Reference | | | | Reference | | | |
|  |  | Q2 | 0.0 | -0.7 | 0.7 | 0.996 | -0.1 | -0.8 | 0.7 | 0.881 | 0.3 | -0.4 | 1.0 | 0.404 |
|  |  | Q3 | -0.7 | -1.5 | 0.0 | 0.053 | -0.6 | -1.4 | 0.2 | 0.114 | 0.2 | -0.6 | 0.9 | 0.686 |
|  |  | Q4 | -1.2 | -1.9 | -0.5 | 0.001 | -1.5 | -2.2 | -0.7 | <0.001 | -0.5 | -1.2 | 0.3 | 0.215 |
|  |  | P for trend | <0.001 | | | | <0.001 | | | | 0.15 | | | |
| PLT | Testosterone | Q1 | Reference | | | | Reference | | | | Reference | | | |
|  |  | Q2 | 4.8 | -10.6 | 20.2 | 0.541 | -4.9 | -20.7 | 10.9 | 0.545 | -2.4 | -17.7 | 12.9 | 0.759 |
|  |  | Q3 | -11.0 | -26.4 | 4.4 | 0.160 | -25.7 | -41.7 | -9.6 | 0.002 | -16.3 | -31.7 | -0.8 | 0.039 |
|  |  | Q4 | -20.5 | -35.9 | -5.1 | 0.009 | -32.8 | -48.8 | -16.8 | <0.001 | -19.3 | -34.8 | -3.7 | 0.015 |
|  |  | P for trend | 0.002 | | | | <0.001 | | | | 0.004 | | | |
|  | Estradiol | Q1 | Reference | | | | Reference | | | | Reference | | | |
|  |  | Q2 | -1.0 | -1.8 | -0.2 | 0.015 | -1.0 | -1.8 | -0.1 | 0.023 | -0.9 | -1.8 | 0.0 | 0.052 |
|  |  | Q3 | -1.1 | -1.9 | -0.2 | 0.011 | -1.1 | -1.9 | -0.2 | 0.018 | -1.2 | -2.1 | -0.3 | 0.010 |
|  |  | Q4 | -1.0 | -1.8 | -0.2 | 0.014 | -0.9 | -1.8 | 0.0 | 0.047 | -1.0 | -1.9 | -0.1 | 0.029 |
|  |  | P for trend | 0.018 | | | | 0.062 | | | | 0.026 | | | |
|  | SHBG | Q1 | Reference | | | | Reference | | | | Reference | | | |
|  |  | Q2 | -5.9 | -8.0 | -3.8 | <0.001 | -1.5 | -3.5 | 0.4 | 0.123 | -1.3 | -3.3 | 0.6 | 0.184 |
|  |  | Q3 | -10.3 | -12.4 | -8.2 | <0.001 | -4.2 | -6.2 | -2.3 | <0.001 | -3.0 | -5.0 | -1.0 | 0.003 |
|  |  | Q4 | -10.6 | -12.7 | -8.5 | <0.001 | -4.4 | -6.4 | -2.4 | <0.001 | -3.1 | -5.1 | -1.1 | 0.002 |
|  |  | P for trend | <0.001 | | | | <0.001 | | | | <0.001 | | | |
|  | FAI | Q1 | Reference | | | | Reference | | | | Reference | | | |
|  |  | Q2 | 118.3 | 69.3 | 167.2 | <0.001 | -5.5 | -48.7 | 37.7 | 0.804 | -2.2 | -47.9 | 43.4 | 0.923 |
|  |  | Q3 | 181.2 | 132.3 | 230.2 | <0.001 | 8.5 | -35.3 | 52.2 | 0.705 | 9.7 | -36.5 | 55.8 | 0.681 |
|  |  | Q4 | 188.6 | 139.7 | 237.6 | <0.001 | 13.6 | -30.2 | 57.4 | 0.543 | 18.7 | -27.7 | 65.2 | 0.429 |
|  |  | P for trend | <0.001 | | | | 0.431 | | | | 0.357 | | | |
|  | TT/E2 | Q1 | Reference | | | | Reference | | | | Reference | | | |
|  |  | Q2 | 0.5 | -0.2 | 1.3 | 0.159 | -0.3 | -1.0 | 0.5 | 0.455 | -0.1 | -0.8 | 0.7 | 0.854 |
|  |  | Q3 | 0.6 | -0.1 | 1.3 | 0.111 | -0.7 | -1.4 | 0.1 | 0.097 | -0.2 | -0.9 | 0.5 | 0.614 |
|  |  | Q4 | 0.4 | -0.3 | 1.1 | 0.292 | -0.9 | -1.6 | -0.1 | 0.028 | -0.4 | -1.2 | 0.3 | 0.269 |
|  |  | P for trend | 0.293 | | | | 0.018 | | | | 0.247 | | | |

Model 1: Unadjusted model. Model 2: Adjusts for age, race, education, marital status, family income to poverty ratio. Model 3: Adjusts for age, race, education, marital status, family income to poverty ratio, body mass index (kg/m^2^), smoking and drinking. TT, Testosterone; E_2_: Estradiol; SHBG: sex hormone-binding globulin; FAI: free androgen index; LC, lymphocyte count; NC, neutrophil count; NLR, neutrophil-to-lymphocyte ratio; PLT, platelet count; PLR, platelet-to-lymphocyte ratio; PPN, the product of platelet count and neutrophil count; SII, systemic immune-inflammation index. Bold fonts indicate P value < 0.05

**Supplementary Table S6.** Sensitivity analysis in Female

| Index | Outcome | Variable | Model1 | | | | Model 2 | | | | Model3 | | | |
| --- | --- | --- | --- | --- | --- | --- | --- | --- | --- | --- | --- | --- | --- | --- |
|  |  |  | β | 95%CI low | 95%CI upp | p-value | β | 95%CI low | 95%CI upp | p-value | β | 95%CI low | 95%CI upp | p-value |
| SII | Testosterone | Q1 | Reference | | | | Reference | | | | Reference | | | |
|  |  | Q2 | -0.2 | -1.8 | 1.5 | 0.861 | -0.1 | -1.8 | 1.6 | 0.928 | -0.2 | -2.0 | 1.6 | 0.848 |
|  |  | Q3 | -0.9 | -2.6 | 0.8 | 0.296 | -0.9 | -2.6 | 0.8 | 0.318 | -0.8 | -2.6 | 1.0 | 0.397 |
|  |  | Q4 | 0.1 | -1.6 | 1.7 | 0.943 | -0.1 | -1.8 | 1.7 | 0.934 | -0.9 | -2.7 | 1.0 | 0.359 |
|  |  | P for trend | 0.836 | | | | 0.723 | | | | 0.284 | | | |
|  | Estradiol | Q1 | Reference | | | | Reference | | | | Reference | | | |
|  |  | Q2 | 12.3 | -3.7 | 28.3 | 0.132 | 12.2 | -5.1 | 29.4 | 0.166 | 8.8 | 1.5 | 16.1 | 0.018 |
|  |  | Q3 | 21.2 | 5.2 | 37.2 | 0.009 | 21.5 | 4.1 | 38.9 | 0.016 | 11.6 | 4.2 | 19.0 | 0.002 |
|  |  | Q4 | 29.8 | 13.8 | 45.8 | <0.001 | 33.2 | 15.8 | 50.6 | 0.000 | 18.2 | 10.8 | 25.6 | <0.001 |
|  |  | P for trend | <0.001 | | | | <0.001 | | | | <0.001 | | | |
|  | SHBG | Q1 | Reference | | | | Reference | | | | Reference | | | |
|  |  | Q2 | -5.5 | -9.6 | -1.5 | 0.007 | -4.7 | -8.9 | -0.5 | 0.028 | -3.4 | -7.5 | 0.7 | 0.101 |
|  |  | Q3 | -10.0 | -14.0 | -6.0 | <0.001 | -9.8 | -14.1 | -5.6 | <0.001 | -6.1 | -10.2 | -2.0 | 0.004 |
|  |  | Q4 | -8.7 | -12.7 | -4.7 | <0.001 | -10.3 | -14.5 | -6.0 | <0.001 | -5.8 | -9.9 | -1.6 | 0.007 |
|  |  | P for trend | <0.001 | | | | <0.001 | | | | 0.003 | | | |
|  | FAI | Q1 | Reference | | | | Reference | | | | Reference | | | |
|  |  | Q2 | 3.8 | -0.2 | 7.7 | 0.060 | 1.7 | -1.5 | 4.9 | 0.300 | 0.9 | -2.4 | 4.2 | 0.585 |
|  |  | Q3 | 5.2 | 1.2 | 9.1 | 0.010 | 4.7 | 1.5 | 7.9 | 0.004 | 2.4 | -0.9 | 5.7 | 0.161 |
|  |  | Q4 | 6.0 | 2.1 | 9.9 | 0.003 | 5.9 | 2.7 | 9.1 | <0.001 | 1.3 | -2.0 | 4.6 | 0.441 |
|  |  | P for trend | 0.002 | | | | <0.001 | | | | 0.323 | | | |
|  | TT/E2 | Q1 | Reference | | | | Reference | | | | Reference | | | |
|  |  | Q2 | -0.1 | -0.3 | 0.1 | 0.299 | -0.1 | -0.2 | 0.1 | 0.553 | 0.0 | -0.2 | 0.2 | 0.919 |
|  |  | Q3 | -0.1 | -0.3 | 0.1 | 0.180 | -0.1 | -0.3 | 0.1 | 0.390 | 0.1 | -0.1 | 0.2 | 0.603 |
|  |  | Q4 | -0.1 | -0.3 | 0.0 | 0.111 | -0.2 | -0.3 | 0.0 | 0.096 | 0.0 | -0.2 | 0.2 | 0.877 |
|  |  | P for trend | 0.108 | | | | 0.096 | | | | 0.967 | | | |
| NLR | Testosterone | Q1 | Reference | | | | Reference | | | | Reference | | | |
|  |  | Q2 | -0.9 | -2.6 | 0.7 | 0.271 | -0.9 | -2.6 | 0.8 | 0.291 | -0.9 | -2.7 | 1.0 | 0.360 |
|  |  | Q3 | -0.9 | -2.6 | 0.7 | 0.279 | 0.0 | -1.7 | 1.7 | 0.990 | 0.0 | -1.8 | 1.9 | 0.964 |
|  |  | Q4 | 0.1 | -1.6 | 1.7 | 0.928 | 0.7 | -1.0 | 2.5 | 0.428 | -0.1 | -2.0 | 1.8 | 0.925 |
|  |  | P for trend | 0.927 | | | | 0.271 | | | | 0.821 | | | |
|  | Estradiol | Q1 | Reference | | | | Reference | | | | Reference | | | |
|  |  | Q2 | 14.3 | -1.7 | 30.3 | 0.081 | 14.3 | -3.1 | 31.7 | 0.107 | 9.3 | 2.0 | 16.6 | 0.013 |
|  |  | Q3 | 14.0 | -2.0 | 30.0 | 0.086 | 19.3 | 1.8 | 36.7 | 0.030 | 15.7 | 8.4 | 23.1 | <0.001 |
|  |  | Q4 | 34.6 | 18.6 | 50.7 | <0.001 | 49.5 | 31.9 | 67.1 | <0.001 | 24.6 | 17.2 | 32.0 | <0.001 |
|  |  | P for trend | <0.001 | | | | <0.001 | | | | <0.001 | | | |
|  | SHBG | Q1 | Reference | | | | Reference | | | | Reference | | | |
|  |  | Q2 | -6.8 | -10.9 | -2.8 | 0.001 | -7.4 | -11.6 | -3.1 | 0.001 | -6.4 | -10.6 | -2.3 | 0.002 |
|  |  | Q3 | -6.2 | -10.3 | -2.2 | 0.002 | -6.7 | -11.0 | -2.4 | 0.002 | -4.6 | -8.8 | -0.5 | 0.028 |
|  |  | Q4 | -3.4 | -7.4 | 0.6 | 0.099 | -6.0 | -10.4 | -1.7 | 0.006 | -4.4 | -8.6 | -0.2 | 0.039 |
|  |  | P for trend | 0.141 | | | | 0.014 | | | | 0.1 | | | |
|  | FAI | Q1 | Reference | | | | Reference | | | | Reference | | | |
|  |  | Q2 | 2.3 | -1.6 | 6.3 | 0.244 | 1.0 | -2.2 | 4.2 | 0.553 | 0.4 | -2.9 | 3.7 | 0.830 |
|  |  | Q3 | 2.6 | -1.3 | 6.6 | 0.186 | 4.1 | 0.9 | 7.3 | 0.013 | 3.2 | -0.2 | 6.5 | 0.062 |
|  |  | Q4 | 1.4 | -2.5 | 5.3 | 0.489 | 3.8 | 0.6 | 7.1 | 0.021 | 0.3 | -3.0 | 3.7 | 0.843 |
|  |  | P for trend | 0.48 | | | | 0.005 | | | | 0.485 | | | |
|  | TT/E2 | Q1 | Reference | | | | Reference | | | | Reference | | | |
|  |  | Q2 | -0.2 | -0.4 | 0.0 | 0.056 | -0.2 | -0.4 | 0.0 | 0.058 | -0.1 | -0.3 | 0.1 | 0.184 |
|  |  | Q3 | -0.1 | -0.3 | 0.1 | 0.364 | -0.1 | -0.3 | 0.1 | 0.297 | 0.0 | -0.2 | 0.2 | 0.859 |
|  |  | Q4 | 0.0 | -0.1 | 0.2 | 0.706 | -0.1 | -0.3 | 0.0 | 0.136 | -0.1 | -0.3 | 0.1 | 0.601 |
|  |  | P for trend | 0.499 | | | | 0.259 | | | | 0.906 | | | |
| PLR | Testosterone | Q1 | Reference | | | | Reference | | | | Reference | | | |
|  |  | Q2 | 0.5 | -1.2 | 2.1 | 0.593 | 0.7 | -1.0 | 2.4 | 0.392 | 1.0 | -0.8 | 2.8 | 0.283 |
|  |  | Q3 | 0.1 | -1.6 | 1.8 | 0.926 | -0.1 | -1.9 | 1.6 | 0.871 | 0.0 | -1.8 | 1.8 | 0.992 |
|  |  | Q4 | -0.6 | -2.3 | 1.1 | 0.476 | -0.1 | -1.8 | 1.6 | 0.917 | -0.3 | -2.2 | 1.5 | 0.725 |
|  |  | P for trend | 0.414 | | | | 0.674 | | | | 0.5 | | | |
|  | Estradiol | Q1 | Reference | | | | Reference | | | | Reference | | | |
|  |  | Q2 | 18.3 | 2.3 | 34.4 | 0.025 | 17.9 | 0.7 | 35.0 | 0.042 | 4.9 | -2.3 | 12.2 | 0.183 |
|  |  | Q3 | 4.8 | -11.2 | 20.8 | 0.558 | 3.7 | -13.7 | 21.0 | 0.680 | 2.1 | -5.2 | 9.4 | 0.578 |
|  |  | Q4 | 14.3 | -1.8 | 30.3 | 0.081 | 21.0 | 3.7 | 38.3 | 0.018 | 6.6 | -0.7 | 14.0 | 0.075 |
|  |  | P for trend | 0.257 | | | | 0.082 | | | | 0.148 | | | |
|  | SHBG | Q1 | Reference | | | | Reference | | | | Reference | | | |
|  |  | Q2 | 0.1 | -3.9 | 4.1 | 0.955 | 0.9 | -3.4 | 5.1 | 0.690 | -0.3 | -4.3 | 3.8 | 0.897 |
|  |  | Q3 | 2.6 | -1.4 | 6.7 | 0.198 | 1.3 | -2.9 | 5.6 | 0.541 | 1.2 | -2.9 | 5.3 | 0.574 |
|  |  | Q4 | 5.2 | 1.2 | 9.2 | 0.011 | 3.7 | -0.6 | 7.9 | 0.091 | 1.5 | -2.6 | 5.6 | 0.486 |
|  |  | P for trend | 0.005 | | | | 0.094 | | | | 0.38 | | | |
|  | FAI | Q1 | Reference | | | | Reference | | | | Reference | | | |
|  |  | Q2 | -0.5 | -4.4 | 3.4 | 0.801 | -1.2 | -4.4 | 2.0 | 0.469 | -0.1 | -3.3 | 3.2 | 0.972 |
|  |  | Q3 | -1.0 | -4.9 | 2.9 | 0.606 | -2.8 | -6.0 | 0.4 | 0.085 | -2.1 | -5.4 | 1.2 | 0.203 |
|  |  | Q4 | -6.3 | -10.2 | -2.4 | 0.002 | -4.8 | -8.0 | -1.6 | 0.004 | -4.3 | -7.6 | -1.1 | 0.010 |
|  |  | P for trend | 0.002 | | | | 0.002 | | | | 0.004 | | | |
|  | TT/E2 | Q1 | Reference | | | | Reference | | | | Reference | | | |
|  |  | Q2 | 0.0 | -0.2 | 0.2 | 0.975 | 0.0 | -0.2 | 0.2 | 0.719 | 0.0 | -0.2 | 0.2 | 0.696 |
|  |  | Q3 | 0.0 | -0.2 | 0.2 | 0.914 | 0.0 | -0.2 | 0.1 | 0.700 | 0.0 | -0.2 | 0.2 | 0.764 |
|  |  | Q4 | 0.1 | -0.1 | 0.2 | 0.450 | 0.0 | -0.2 | 0.2 | 0.752 | 0.0 | -0.2 | 0.2 | 0.874 |
|  |  | P for trend | 0.489 | | | | 0.593 | | | | 0.714 | | | |
| PPN | Testosterone | Q1 | Reference | | | | Reference | | | | Reference | | | |
|  |  | Q2 | 0.5 | -1.2 | 2.1 | 0.576 | 0.0 | -1.7 | 1.7 | 0.992 | 0.3 | -1.5 | 2.1 | 0.753 |
|  |  | Q3 | -0.6 | -2.3 | 1.1 | 0.487 | -1.2 | -2.9 | 0.6 | 0.182 | -1.3 | -3.1 | 0.6 | 0.169 |
|  |  | Q4 | 1.0 | -0.7 | 2.7 | 0.230 | -0.3 | -2.0 | 1.5 | 0.753 | -0.9 | -2.8 | 1.0 | 0.368 |
|  |  | P for trend | 0.458 | | | | 0.483 | | | | 0.17 | | | |
|  | Estradiol | Q1 | Reference | | | | Reference | | | | Reference | | | |
|  |  | Q2 | 20.4 | 4.4 | 36.4 | 0.013 | 16.5 | -0.9 | 33.8 | 0.062 | 4.5 | -2.9 | 11.8 | 0.236 |
|  |  | Q3 | 16.7 | 0.7 | 32.7 | 0.041 | 10.6 | -6.8 | 28.1 | 0.233 | 6.8 | -0.6 | 14.2 | 0.072 |
|  |  | Q4 | 33.6 | 17.6 | 49.6 | <0.001 | 23.3 | 5.7 | 40.8 | 0.010 | 13.8 | 6.2 | 21.5 | 0.000 |
|  |  | P for trend | <0.001 | | | | 0.025 | | | | <0.001 | | | |
|  | SHBG | Q1 | Reference | | | | Reference | | | | Reference | | | |
|  |  | Q2 | -7.9 | -11.9 | -4.0 | <0.001 | -7.9 | -12.1 | -3.6 | <0.001 | -5.9 | -10.0 | -1.8 | 0.005 |
|  |  | Q3 | -15.7 | -19.7 | -11.7 | <0.001 | -14.8 | -19.0 | -10.5 | <0.001 | -9.9 | -14.1 | -5.8 | <0.001 |
|  |  | Q4 | -18.0 | -22.0 | -14.0 | <0.001 | -17.8 | -22.0 | -13.5 | <0.001 | -9.8 | -14.1 | -5.5 | <0.001 |
|  |  | P for trend | <0.001 | | | | <0.001 | | | | <0.001 | | | |
|  | FAI | Q1 | Reference | | | | Reference | | | | Reference | | | |
|  |  | Q2 | 8.6 | 4.7 | 12.5 | <0.001 | 5.9 | 2.7 | 9.1 | <0.001 | 4.9 | 1.6 | 8.2 | 0.004 |
|  |  | Q3 | 9.1 | 5.2 | 13.0 | <0.001 | 7.1 | 3.9 | 10.3 | <0.001 | 3.9 | 0.6 | 7.2 | 0.021 |
|  |  | Q4 | 15.9 | 12.0 | 19.8 | <0.001 | 12.4 | 9.1 | 15.6 | <0.001 | 6.3 | 2.8 | 9.7 | <0.001 |
|  |  | P for trend | <0.001 | | | | <0.001 | | | | 0.001 | | | |
|  | TT/E2 | Q1 | Reference | | | | Reference | | | | Reference | | | |
|  |  | Q2 | -0.2 | -0.4 | 0.0 | 0.017 | -0.1 | -0.3 | 0.0 | 0.133 | -0.1 | -0.3 | 0.1 | 0.428 |
|  |  | Q3 | -0.3 | -0.4 | -0.1 | 0.005 | -0.1 | -0.3 | 0.1 | 0.334 | 0.0 | -0.2 | 0.2 | 0.692 |
|  |  | Q4 | -0.4 | -0.6 | -0.2 | <0.001 | -0.2 | -0.4 | -0.1 | 0.013 | 0.0 | -0.2 | 0.2 | 0.683 |
|  |  | P for trend | <0.001 | | | | 0.028 | | | | 0.984 | | | |
| LC | Testosterone | Q1 | Reference | | | | Reference | | | | Reference | | | |
|  |  | Q2 | 1.0 | -0.7 | 2.6 | 0.256 | -0.1 | -1.8 | 1.6 | 0.917 | -0.1 | -1.9 | 1.8 | 0.935 |
|  |  | Q3 | 1.9 | 0.3 | 3.6 | 0.025 | 0.3 | -1.5 | 2.0 | 0.750 | 1.0 | -0.9 | 2.8 | 0.311 |
|  |  | Q4 | 1.1 | -0.6 | 2.7 | 0.194 | -0.4 | -2.1 | 1.3 | 0.660 | -0.3 | -2.2 | 1.5 | 0.739 |
|  |  | P for trend | 0.121 | | | | 0.761 | | | | 0.988 | | | |
|  | Estradiol | Q1 | Reference | | | | Reference | | | | Reference | | | |
|  |  | Q2 | 19.9 | 3.8 | 36.0 | 0.015 | 11.0 | -6.4 | 28.3 | 0.217 | -0.6 | -7.9 | 6.8 | 0.876 |
|  |  | Q3 | 14.4 | -1.8 | 30.6 | 0.081 | -2.1 | -19.8 | 15.6 | 0.818 | 1.4 | -6.1 | 8.9 | 0.710 |
|  |  | Q4 | 5.1 | -10.7 | 21.0 | 0.526 | -15.0 | -32.4 | 2.4 | 0.091 | -7.1 | -14.5 | 0.3 | 0.060 |
|  |  | P for trend | 0.731 | | | | 0.033 | | | | 0.094 | | | |
|  | SHBG | Q1 | Reference | | | | Reference | | | | Reference | | | |
|  |  | Q2 | -9.4 | -13.4 | -5.4 | <0.001 | -7.5 | -11.8 | -3.3 | 0.001 | -4.8 | -8.9 | -0.7 | 0.023 |
|  |  | Q3 | -8.9 | -12.9 | -4.9 | <0.001 | -7.6 | -11.9 | -3.3 | 0.001 | -3.3 | -7.5 | 0.9 | 0.118 |
|  |  | Q4 | -18.3 | -22.2 | -14.3 | <0.001 | -15.8 | -20.1 | -11.6 | <0.001 | -8.4 | -12.5 | -4.2 | <0.001 |
|  |  | P for trend | <0.001 | | | | <0.001 | | | | <0.001 | | | |
|  | FAI | Q1 | Reference | | | | Reference | | | | Reference | | | |
|  |  | Q2 | 8.6 | 4.7 | 12.5 | <0.001 | 3.6 | 0.4 | 6.8 | 0.026 | 2.2 | -1.1 | 5.5 | 0.193 |
|  |  | Q3 | 9.0 | 5.1 | 13.0 | <0.001 | 4.0 | 0.7 | 7.2 | 0.017 | 2.8 | -0.6 | 6.1 | 0.104 |
|  |  | Q4 | 15.6 | 11.8 | 19.5 | <0.001 | 10.8 | 7.6 | 14.0 | <0.001 | 7.4 | 4.1 | 10.7 | <0.001 |
|  |  | P for trend | <0.001 | | | | <0.001 | | | | <0.001 | | | |
|  | TT/E2 | Q1 | Reference | | | | Reference | | | | Reference | | | |
|  |  | Q2 | -0.3 | -0.4 | -0.1 | 0.005 | -0.1 | -0.3 | 0.1 | 0.324 | -0.1 | -0.3 | 0.1 | 0.403 |
|  |  | Q3 | -0.4 | -0.6 | -0.2 | <0.001 | -0.1 | -0.3 | 0.1 | 0.142 | -0.1 | -0.3 | 0.1 | 0.401 |
|  |  | Q4 | -0.3 | -0.5 | -0.2 | <0.001 | 0.0 | -0.2 | 0.2 | 0.814 | 0.1 | -0.1 | 0.3 | 0.443 |
|  |  | P for trend | <0.001 | | | | 0.751 | | | | 0.437 | | | |
| NC | Testosterone | Q1 | Reference | | | | Reference | | | | Reference | | | |
|  |  | Q2 | -0.1 | -1.8 | 1.6 | 0.943 | 0.5 | -1.2 | 2.3 | 0.549 | 0.3 | -1.6 | 2.1 | 0.766 |
|  |  | Q3 | -1.0 | -2.7 | 0.7 | 0.258 | -0.7 | -2.5 | 1.0 | 0.413 | -1.1 | -3.1 | 0.8 | 0.246 |
|  |  | Q4 | 0.4 | -1.3 | 2.1 | 0.611 | 0.0 | -1.8 | 1.8 | 0.990 | -0.9 | -2.8 | 1.1 | 0.385 |
|  |  | P for trend | 0.855 | | | | 0.632 | | | | 0.192 | | | |
|  | Estradiol | Q1 | Reference | | | | Reference | | | | Reference | | | |
|  |  | Q2 | 7.8 | -8.5 | 24.0 | 0.350 | 11.0 | -6.7 | 28.7 | 0.224 | 5.4 | -2.1 | 12.9 | 0.155 |
|  |  | Q3 | 21.4 | 5.1 | 37.7 | 0.010 | 22.1 | 4.1 | 40.1 | 0.016 | 10.5 | 2.8 | 18.2 | 0.007 |
|  |  | Q4 | 32.4 | 16.2 | 48.7 | <0.001 | 33.6 | 15.5 | 51.6 | <0.001 | 20.2 | 12.3 | 28.0 | <0.001 |
|  |  | P for trend | <0.001 | | | | <0.001 | | | | <0.001 | | | |
|  | SHBG | Q1 | Reference | | | | Reference | | | | Reference | | | |
|  |  | Q2 | -7.9 | -12.0 | -3.9 | <0.001 | -7.6 | -11.9 | -3.3 | 0.001 | -6.0 | -10.2 | -1.8 | 0.005 |
|  |  | Q3 | -13.9 | -17.9 | -9.8 | <0.001 | -13.9 | -18.3 | -9.5 | <0.001 | -9.4 | -13.7 | -5.1 | <0.001 |
|  |  | Q4 | -17.6 | -21.7 | -13.6 | <0.001 | -18.7 | -23.1 | -14.3 | <0.001 | -11.4 | -15.8 | -7.1 | <0.001 |
|  |  | P for trend | <0.001 | | | | <0.001 | | | | <0.001 | | | |
|  | FAI | Q1 | Reference | | | | Reference | | | | Reference | | | |
|  |  | Q2 | 6.2 | 2.3 | 10.2 | 0.002 | 6.0 | 2.8 | 9.3 | <0.001 | 4.5 | 1.2 | 7.9 | 0.008 |
|  |  | Q3 | 8.0 | 4.0 | 12.0 | <0.001 | 8.5 | 5.2 | 11.8 | <0.001 | 5.4 | 1.9 | 8.8 | 0.002 |
|  |  | Q4 | 13.9 | 9.9 | 17.8 | <0.001 | 13.0 | 9.7 | 16.3 | <0.001 | 6.7 | 3.2 | 10.2 | <0.001 |
|  |  | P for trend | <0.001 | | | | <0.001 | | | | <0.001 | | | |
|  | TT/E2 | Q1 | Reference | | | | Reference | | | | Reference | | | |
|  |  | Q2 | -0.1 | -0.3 | 0.0 | 0.136 | -0.1 | -0.3 | 0.1 | 0.218 | -0.1 | -0.2 | 0.2 | 0.641 |
|  |  | Q3 | -0.1 | -0.3 | 0.1 | 0.160 | -0.1 | -0.3 | 0.1 | 0.483 | 0.1 | -0.1 | 0.3 | 0.517 |
|  |  | Q4 | -0.3 | -0.5 | -0.1 | 0.002 | -0.2 | -0.4 | -0.1 | 0.014 | -0.1 | -0.3 | 0.2 | 0.594 |
|  |  | P for trend | 0.004 | | | | 0.03 | | | | 0.867 | | | |
| PLT | Testosterone | Q1 | Reference | | | | Reference | | | | Reference | | | |
|  |  | Q2 | 0.5 | -1.2 | 2.2 | 0.555 | 0.3 | -1.4 | 2.0 | 0.708 | 0.4 | -1.4 | 2.2 | 0.673 |
|  |  | Q3 | 1.4 | -0.3 | 3.1 | 0.104 | 0.2 | -1.5 | 1.9 | 0.824 | 0.5 | -1.4 | 2.3 | 0.611 |
|  |  | Q4 | 0.7 | -1.0 | 2.4 | 0.423 | -0.8 | -2.5 | 0.9 | 0.368 | -1.0 | -2.9 | 0.9 | 0.293 |
|  |  | P for trend | 0.28 | | | | 0.358 | | | | 0.325 | | | |
|  | Estradiol | Q1 | Reference | | | | Reference | | | | Reference | | | |
|  |  | Q2 | -3.8 | -19.8 | 12.3 | 0.646 | -15.7 | -33.1 | 1.6 | 0.076 | -8.2 | -15.5 | -0.9 | 0.029 |
|  |  | Q3 | 7.5 | -8.7 | 23.6 | 0.364 | -8.0 | -25.4 | 9.5 | 0.371 | -2.0 | -9.3 | 5.4 | 0.604 |
|  |  | Q4 | 8.7 | -7.4 | 24.7 | 0.289 | -12.0 | -29.5 | 5.5 | 0.180 | -2.5 | -10.0 | 4.9 | 0.506 |
|  |  | P for trend | 0.148 | | | | 0.331 | | | | 0.933 | | | |
|  | SHBG | Q1 | Reference | | | | Reference | | | | Reference | | | |
|  |  | Q2 | -5.1 | -9.1 | -1.1 | 0.012 | -4.2 | -8.5 | 0.0 | 0.049 | -2.3 | -6.4 | 1.8 | 0.277 |
|  |  | Q3 | -13.7 | -17.7 | -9.6 | <0.001 | -12.9 | -17.1 | -8.6 | <0.001 | -8.6 | -12.7 | -4.5 | <0.001 |
|  |  | Q4 | -14.3 | -18.3 | -10.3 | <0.001 | -12.8 | -17.0 | -8.5 | <0.001 | -5.8 | -9.9 | -1.6 | 0.007 |
|  |  | P for trend | |  |  |  | <0.001 | | | | <0.001 | | | |
|  | FAI | Q1 | Reference | | | | Reference | | | | Reference | | | |
|  |  | Q2 | 4.5 | 0.6 | 8.4 | 0.023 | 3.3 | 0.1 | 6.5 | 0.046 | 2.0 | -1.3 | 5.3 | 0.235 |
|  |  | Q3 | 11.4 | 7.4 | 15.3 | <0.001 | 6.6 | 3.3 | 9.8 | <0.001 | 4.7 | 1.5 | 8.0 | 0.005 |
|  |  | Q4 | 10.4 | 6.5 | 14.3 | <0.001 | 5.5 | 2.3 | 8.7 | 0.001 | 1.4 | -2.0 | 4.7 | 0.418 |
|  |  | P for trend | <0.001 | | | | <0.001 | | | | 0.209 | | | |
|  | TT/E2 | Q1 | Reference | | | | Reference | | | | Reference | | | |
|  |  | Q2 | -0.2 | -0.3 | 0.0 | 0.080 | 0.0 | -0.2 | 0.2 | 0.911 | 0.1 | -0.1 | 0.3 | 0.461 |
|  |  | Q3 | -0.3 | -0.5 | -0.1 | 0.001 | -0.1 | -0.3 | 0.1 | 0.353 | 0.0 | -0.2 | 0.2 | 0.891 |
|  |  | Q4 | -0.4 | -0.6 | -0.3 | <0.001 | -0.1 | -0.3 | 0.1 | 0.199 | 0.0 | -0.2 | 0.2 | 0.887 |
|  |  | P for trend | <0.001 | | | | 0.12 | | | | 0.885 | | | |

Model 1: Unadjusted model. Model 2: Adjusts for age, race, education, marital status, family income to poverty ratio. Model 3: Adjusts for age, race, education, marital status, family income to poverty ratio, body mass index (kg/m^2^), smoking and drinking. TT, Testosterone; E_2_: Estradiol; SHBG: sex hormone-binding globulin; FAI: free androgen index; LC, lymphocyte count; NC, neutrophil count; NLR, neutrophil-to-lymphocyte ratio; PLT, platelet count; PLR, platelet-to-lymphocyte ratio; PPN, the product of platelet count and neutrophil count; SII, systemic immune-inflammation index. Bold fonts indicate P value < 0.05.

**Supplementary Table S7.** Association between Eosinophil and sex hormones in both gender

| Index | Outcome | Model1 | | | | Model 2 | | | | Model3 | | | |
| --- | --- | --- | --- | --- | --- | --- | --- | --- | --- | --- | --- | --- | --- |
| Male |  | β | 95%CI low | 95%CI upp | p-value | β | 95%CI low | 95%CI upp | p-value | β | 95%CI low | 95%CI upp | p-value |
| Eosinophil | Testosterone | -56.4 | -84.3 | -28.6 | <0.001 | -44.7 | -73.2 | -16.2 | 0.002 | -24.2 | -50.7 | 2.4 | 0.074 |
|  | Estradiol | 0.0 | -1.5 | 1.5 | 0.984 | 0.6 | -0.9 | 2.2 | 0.420 | 0.2 | -1.4 | 1.7 | 0.851 |
|  | SHBG | -1.9 | -5.8 | 2.0 | 0.335 | -4.9 | -8.4 | -1.4 | 0.007 | -2.6 | -6.0 | 0.8 | 0.129 |
|  | FAI | -69.2 | -158.7 | 20.2 | 0.129 | 42.3 | -35.4 | 119.9 | 0.286 | 45.6 | -32.7 | 123.9 | 0.254 |
|  | TT/E2 | -1.2 | -2.5 | 0.2 | 0.083 | -0.4 | -1.8 | 1.0 | 0.567 | 0.1 | -1.2 | 1.5 | 0.850 |
| Female |  |  |  |  |  |  |  |  |  |  |  |  |  |
| Eosinophil | Testosterone | 0.2 | -0.2 | 0.5 | 0.338 | 0.0 | -0.3 | 0.4 | 0.881 | -0.2 | -0.6 | 0.2 | 0.347 |
|  | Estradiol | 6.5 | 3.3 | 9.7 | <0.001 | 6.5 | 3.0 | 10.0 | <0.001 | 4.2 | 2.7 | 5.8 | <0.001 |
|  | SHBG | -3.0 | -3.8 | -2.2 | <0.001 | -3.2 | -4.0 | -2.3 | <0.001 | -1.7 | -2.6 | -0.9 | <0.001 |
|  | FAI | 2.6 | 1.8 | 3.4 | <0.001 | 2.4 | 1.8 | 3.1 | <0.001 | 1.1 | 0.4 | 1.7 | 0.003 |
|  | TT/E2 | -0.1 | -0.1 | 0.0 | 0.007 | 0.0 | -0.1 | 0.0 | 0.036 | 0.0 | 0.0 | 0.0 | 0.967 |

Model 1: Unadjusted model. Model 2: Adjusts for age, race, education, marital status, family income to poverty ratio. Model 3: Adjusts for age, race, education, marital status, family income to poverty ratio, body mass index (kg/m2), smoking and drinking.

**Supplementary Table S8.** Literature normal ranges of total sex hormones in serum

| Analyte - Sex | Group | Value Range |
| --- | --- | --- |
| **Total Testosterone** |  |  |
| Male | Adult | 280-1100 (ng/dL) |
| Female | Adult | 15-70 (ng/dL) |
| **Total Estradiol** |  |  |
| Male | Adult | 10 - 50 (pg/mL) |
| Female | Premenopausal | 20 - 350 (pg/mL) |
|  | Postmenopausal | ≤ 20 (pg/mL) |
| **SHBG** |  |  |
| Male | Adult | 10 – 57 (nmol/L) |
| Female | Adult | 18 – 144 (nmol/L) |

**Supplementary Table S9.** Association between [Inflammation](https://www.frontiersin.org/journals/immunology/sections/inflammation)-related Index and sex hormones in Female postmenopausal group

| Index | Outcome | Model1 | | | | Model 2 | | | | Model3 | | | |
| --- | --- | --- | --- | --- | --- | --- | --- | --- | --- | --- | --- | --- | --- |
|  |  | β | 95%CI low | 95%CI upp | p-value | β | 95%CI low | 95%CI upp | p-value | β | 95%CI low | 95%CI upp | p-value |
| Log2-SII | Testosterone | -1.2 | -2.2 | -0.2 | 0.018 | -1.1 | -2.1 | -0.2 | 0.025 | -1.2 | -2.2 | -0.2 | 0.022 |
|  | Estradiol | 1.4 | -0.4 | 3.1 | 0.120 | 1.8 | 0.2 | 3.5 | 0.033 | 1.6 | -0.1 | 3.3 | 0.057 |
|  | SHBG | -4.6 | -6.7 | -2.4 | <0.001 | -5.9 | -8.0 | -3.8 | <0.001 | -4.2 | -6.2 | -2.1 | <0.001 |
|  | FAI | 0.3 | -2.5 | 3.1 | 0.832 | 0.9 | -2.0 | 3.7 | 0.542 | 0.1 | -2.8 | 3.0 | 0.945 |
|  | TT/E2 | 0.0 | -0.1 | 0.2 | 0.597 | 0.0 | -0.1 | 0.2 | 0.834 | 0.1 | -0.1 | 0.2 | 0.314 |
| Log2-NLR | Testosterone | -1.3 | -2.4 | -0.1 | 0.027 | -1.3 | -2.4 | -0.1 | 0.035 | -1.3 | -2.5 | -0.1 | 0.032 |
|  | Estradiol | 0.1 | -1.9 | 2.1 | 0.914 | 2.4 | 0.5 | 4.3 | 0.015 | 2.2 | 0.3 | 4.2 | 0.024 |
|  | SHBG | -0.5 | -3.0 | 2.0 | 0.698 | -3.7 | -6.2 | -1.2 | 0.004 | -1.8 | -4.2 | 0.6 | 0.140 |
|  | FAI | -1.9 | -5.1 | 1.4 | 0.259 | -0.6 | -3.9 | 2.8 | 0.750 | -1.4 | -4.8 | 1.9 | 0.404 |
|  | TT/E2 | 0.1 | -0.1 | 0.3 | 0.297 | 0.0 | -0.2 | 0.2 | 0.961 | 0.1 | -0.1 | 0.2 | 0.460 |
| Log2-PLR | Testosterone | -1.0 | -2.4 | 0.4 | 0.159 | -1.2 | -2.6 | 0.2 | 0.103 | -1.2 | -2.6 | 0.2 | 0.103 |
|  | Estradiol | -0.3 | -2.7 | 2.2 | 0.844 | 0.4 | -2.0 | 2.7 | 0.741 | 0.5 | -1.9 | 2.9 | 0.671 |
|  | SHBG | 4.9 | 1.7 | 8.0 | 0.002 | 3.0 | -0.1 | 6.1 | 0.055 | 1.3 | -1.7 | 4.2 | 0.396 |
|  | FAI | -4.4 | -8.5 | -0.4 | 0.030 | -3.9 | -7.9 | 0.2 | 0.061 | -3.1 | -7.1 | 1.0 | 0.139 |
|  | TT/E2 | 0.3 | 0.1 | 0.5 | 0.013 | 0.2 | 0.0 | 0.4 | 0.041 | 0.2 | 0.0 | 0.4 | 0.103 |
| Log2-PPN | Testosterone | -1.0 | -2.0 | 0.0 | 0.058 | -0.9 | -1.9 | 0.2 | 0.106 | -0.9 | -2.0 | 0.1 | 0.089 |
|  | Estradiol | 3.0 | 1.2 | 4.8 | 0.001 | 1.8 | 0.1 | 3.6 | 0.040 | 1.5 | -0.2 | 3.3 | 0.090 |
|  | SHBG | -12.0 | -14.2 | -9.8 | <0.001 | -11.5 | -13.7 | -9.3 | <0.001 | -8.6 | -10.7 | -6.4 | <0.001 |
|  | FAI | 4.5 | 1.6 | 7.4 | 0.003 | 4.4 | 1.5 | 7.4 | 0.003 | 3.1 | 0.1 | 6.1 | 0.046 |
|  | TT/E2 | -0.1 | -0.3 | 0.0 | 0.104 | -0.1 | -0.2 | 0.1 | 0.272 | 0.0 | -0.1 | 0.2 | 0.799 |
| Log2-LC | Testosterone | 0.6 | -0.9 | 2.1 | 0.418 | 0.8 | -0.7 | 2.4 | 0.289 | 0.8 | -0.7 | 2.4 | 0.294 |
|  | Estradiol | 3.2 | 0.6 | 5.8 | 0.015 | -0.3 | -2.9 | 2.2 | 0.794 | -0.6 | -3.2 | 1.9 | 0.630 |
|  | SHBG | -15.1 | -18.4 | -11.9 | <0.001 | -11.1 | -14.4 | -7.8 | <0.001 | -8.3 | -11.4 | -5.1 | <0.001 |
|  | FAI | 8.9 | 4.7 | 13.1 | <0.001 | 7.5 | 3.2 | 11.9 | 0.001 | 6.3 | 1.9 | 10.7 | 0.005 |
|  | TT/E2 | -0.3 | -0.6 | -0.1 | 0.001 | -0.2 | -0.4 | 0.0 | 0.052 | -0.1 | -0.4 | 0.1 | 0.237 |
| Log2-NC | Testosterone | -1.4 | -2.8 | 0.0 | 0.051 | -1.2 | -2.6 | 0.3 | 0.116 | -1.3 | -2.8 | 0.2 | 0.092 |
|  | Estradiol | 3.0 | 0.6 | 5.4 | 0.016 | 3.3 | 0.9 | 5.7 | 0.006 | 3.0 | 0.5 | 5.5 | 0.017 |
|  | SHBG | -13.8 | -16.8 | -10.8 | <0.001 | -15.1 | -18.1 | -12.1 | <0.001 | -10.4 | -13.4 | -7.4 | <0.001 |
|  | FAI | 4.9 | 0.9 | 8.8 | 0.016 | 5.7 | 1.7 | 9.8 | 0.006 | 3.5 | -0.7 | 7.7 | 0.103 |
|  | TT/E2 | -0.2 | -0.4 | 0.0 | 0.093 | -0.2 | -0.4 | 0.0 | 0.080 | 0.0 | -0.2 | 0.2 | 0.840 |
| Log2-PLT | Testosterone | -1.0 | -3.0 | 1.1 | 0.351 | -1.0 | -3.1 | 1.1 | 0.342 | -1.0 | -3.1 | 1.1 | 0.332 |
|  | Estradiol | 5.6 | 2.0 | 9.2 | 0.002 | 0.2 | -3.2 | 3.7 | 0.895 | -0.1 | -3.5 | 3.4 | 0.977 |
|  | SHBG | -18.6 | -23.1 | -14.0 | <0.001 | -14.2 | -18.7 | -9.7 | <0.001 | -12.4 | -16.7 | -8.2 | <0.001 |
|  | FAI | 7.5 | 1.6 | 13.4 | 0.012 | 5.6 | -0.3 | 11.6 | 0.064 | 5.0 | -1.0 | 10.9 | 0.104 |
|  | TT/E2 | -0.1 | -0.4 | 0.2 | 0.429 | 0.1 | -0.2 | 0.4 | 0.724 | 0.1 | -0.2 | 0.4 | 0.429 |

Model 1: Unadjusted model. Model 2: Adjusts for age, race, education, marital status, family income to poverty ratio. Model 3: Adjusts for age, race, education, marital status, family income to poverty ratio, body mass index (kg/m^2^), smoking and drinking. TT, Testosterone; E_2_: Estradiol; SHBG: sex hormone-binding globulin; FAI: free androgen index; LC, lymphocyte count; NC, neutrophil count; NLR, neutrophil-to-lymphocyte ratio; PLT, platelet count; PLR, platelet-to-lymphocyte ratio; PPN, the product of platelet count and neutrophil count; SII, systemic immune-inflammation index. Bold fonts indicate P value < 0.05.

**Supplementary Table S10.** Association between [Inflammation](https://www.frontiersin.org/journals/immunology/sections/inflammation)-related Index and sex hormones in Female premenopausal group

| Index | Outcome | Model1 | | | | Model 2 | | | | Model3 | | | |
| --- | --- | --- | --- | --- | --- | --- | --- | --- | --- | --- | --- | --- | --- |
|  |  | β | 95%CI low | 95%CI upp | p-value | β | 95%CI low | 95%CI upp | p-value | β | 95%CI low | 95%CI upp | p-value |
| Log2-SII | Testosterone | 0.5 | -0.7 | 1.6 | 0.461 | 0.5 | -0.7 | 1.7 | 0.433 | 0.1 | -1.1 | 1.4 | 0.843 |
|  | Estradiol | 19.6 | 5.5 | 33.7 | 0.007 | 24.9 | 10.5 | 39.4 | 0.001 | 25.2 | 10.4 | 40.0 | 0.001 |
|  | SHBG | -4.6 | -7.7 | -1.6 | 0.003 | -4.9 | -8.0 | -1.9 | 0.002 | -1.4 | -4.4 | 1.7 | 0.382 |
|  | FAI | 4.5 | 2.2 | 6.9 | <0.001 | 4.8 | 2.5 | 7.2 | <0.001 | 1.3 | -1.1 | 3.6 | 0.283 |
|  | TT/E2 | -0.1 | -0.2 | 0.0 | 0.030 | -0.1 | -0.2 | -0.1 | 0.002 | -0.1 | -0.2 | 0.0 | 0.003 |
| Log2-NLR | Testosterone | 0.4 | -1.0 | 1.8 | 0.594 | 0.7 | -0.8 | 2.1 | 0.375 | 0.3 | -1.2 | 1.8 | 0.701 |
|  | Estradiol | 34.8 | 17.9 | 51.7 | <0.001 | 43.9 | 26.4 | 61.5 | <0.001 | 43.5 | 25.7 | 61.2 | <0.001 |
|  | SHBG | -1.3 | -4.9 | 2.3 | 0.476 | -2.2 | -5.9 | 1.5 | 0.245 | 0.4 | -3.2 | 4.0 | 0.835 |
|  | FAI | 2.5 | -0.3 | 5.3 | 0.079 | 3.7 | 0.8 | 6.5 | 0.012 | 0.9 | -1.8 | 3.7 | 0.509 |
|  | TT/E2 | -0.1 | -0.2 | 0.0 | 0.290 | -0.1 | -0.2 | 0.0 | 0.017 | -0.1 | -0.2 | 0.0 | 0.024 |
| Log2-PLR | Testosterone | -0.4 | -2.2 | 1.3 | 0.638 | 0.2 | -1.6 | 1.9 | 0.843 | 0.3 | -1.4 | 2.0 | 0.730 |
|  | Estradiol | 4.0 | -16.7 | 24.7 | 0.706 | 7.5 | -13.4 | 28.5 | 0.480 | 7.6 | -13.4 | 28.6 | 0.477 |
|  | SHBG | 2.1 | -2.3 | 6.5 | 0.353 | 2.0 | -2.4 | 6.4 | 0.372 | 2.1 | -2.2 | 6.4 | 0.346 |
|  | FAI | -5.0 | -8.4 | -1.5 | 0.005 | -3.2 | -6.6 | 0.2 | 0.064 | -3.1 | -6.4 | 0.2 | 0.067 |
|  | TT/E2 | -0.1 | -0.2 | 0.0 | 0.049 | -0.2 | -0.3 | 0.0 | 0.010 | -0.2 | -0.3 | 0.0 | 0.012 |
| Log2-PPN | Testosterone | 0.8 | -0.4 | 1.9 | 0.197 | 0.4 | -0.8 | 1.6 | 0.503 | -0.1 | -1.3 | 1.1 | 0.870 |
|  | Estradiol | 12.1 | -1.4 | 25.6 | 0.079 | 15.2 | 1.4 | 29.1 | 0.031 | 15.9 | 1.3 | 30.4 | 0.033 |
|  | SHBG | -8.5 | -11.4 | -5.7 | <0.001 | -8.6 | -11.4 | -5.7 | <0.001 | -3.9 | -6.8 | -0.9 | 0.011 |
|  | FAI | 8.8 | 6.5 | 11.0 | <0.001 | 8.0 | 5.7 | 10.2 | <0.001 | 3.3 | 1.0 | 5.6 | 0.005 |
|  | TT/E2 | -0.1 | -0.2 | 0.0 | 0.043 | -0.1 | -0.2 | 0.0 | 0.013 | -0.1 | -0.2 | 0.0 | 0.027 |
| Log2-LC | Testosterone | 1.0 | -1.0 | 2.9 | 0.318 | -0.1 | -2.1 | 1.8 | 0.891 | -0.6 | -2.6 | 1.4 | 0.558 |
|  | Estradiol | -16.9 | -39.8 | 6.1 | 0.151 | -21.5 | -44.9 | 1.8 | 0.071 | -22.3 | -46.1 | 1.4 | 0.066 |
|  | SHBG | -12.5 | -17.4 | -7.6 | <0.001 | -11.5 | -16.4 | -6.6 | <0.001 | -6.8 | -11.7 | -2.0 | 0.006 |
|  | FAI | 13.4 | 9.6 | 17.2 | <0.001 | 10.1 | 6.3 | 13.9 | <0.001 | 5.5 | 1.8 | 9.2 | 0.004 |
|  | TT/E2 | 0.0 | -0.1 | 0.1 | 0.931 | 0.1 | -0.1 | 0.2 | 0.349 | 0.1 | -0.1 | 0.2 | 0.255 |
| Log2-NC | Testosterone | 1.0 | -0.5 | 2.5 | 0.185 | 0.7 | -0.9 | 2.2 | 0.409 | -0.1 | -1.6 | 1.5 | 0.953 |
|  | Estradiol | 27.9 | 10.2 | 45.5 | 0.002 | 34.9 | 16.4 | 53.3 | <0.001 | 36.1 | 16.9 | 55.3 | <0.001 |
|  | SHBG | -8.8 | -12.6 | -5.0 | <0.001 | -9.6 | -13.5 | -5.7 | <0.001 | -4.0 | -7.9 | -0.1 | 0.046 |
|  | FAI | 10.7 | 7.8 | 13.6 | <0.001 | 10.4 | 7.4 | 13.4 | <0.001 | 4.7 | 1.7 | 7.7 | 0.002 |
|  | TT/E2 | -0.1 | -0.2 | 0.1 | 0.299 | -0.1 | -0.2 | 0.0 | 0.076 | -0.1 | -0.2 | 0.0 | 0.128 |
| Log2-PLT | Testosterone | 0.7 | -1.7 | 3.2 | 0.552 | 0.1 | -2.3 | 2.5 | 0.916 | -0.3 | -2.8 | 2.2 | 0.807 |
|  | Estradiol | -18.7 | -47.4 | 10.1 | 0.203 | -18.7 | -47.7 | 10.3 | 0.207 | -19.6 | -49.3 | 10.1 | 0.195 |
|  | SHBG | -15.5 | -21.6 | -9.3 | <0.001 | -13.8 | -19.9 | -7.8 | <0.001 | -6.5 | -12.6 | -0.4 | 0.036 |
|  | FAI | 11.5 | 6.7 | 16.2 | <0.001 | 9.4 | 4.6 | 14.1 | <0.001 | 2.5 | -2.2 | 7.1 | 0.296 |
|  | TT/E2 | -0.2 | -0.4 | -0.1 | 0.009 | -0.2 | -0.4 | 0.0 | 0.015 | -0.2 | -0.3 | 0.0 | 0.031 |

Model 1: Unadjusted model. Model 2: Adjusts for age, race, education, marital status, family income to poverty ratio. Model 3: Adjusts for age, race, education, marital status, family income to poverty ratio, body mass index (kg/m^2^), smoking and drinking. TT, Testosterone; E_2_: Estradiol; SHBG: sex hormone-binding globulin; FAI: free androgen index; LC, lymphocyte count; NC, neutrophil count; NLR, neutrophil-to-lymphocyte ratio; PLT, platelet count; PLR, platelet-to-lymphocyte ratio; PPN, the product of platelet count and neutrophil count; SII, systemic immune-inflammation index. Bold fonts indicate P value < 0.05.

**Supplementary Table S11.** Association between [inflammation](https://www.frontiersin.org/journals/immunology/sections/inflammation)-related index and sex hormones disorder in Male

| Index | Outcome | Model1 | | | | Model 2 | | | | Model3 | | | |
| --- | --- | --- | --- | --- | --- | --- | --- | --- | --- | --- | --- | --- | --- |
|  |  | OR | 95%CI low | 95%CI upp | p-value | OR | 95%CI low | 95%CI upp | p-value | OR | 95%CI low | 95%CI upp | p-value |
| Log2-SII | Testosterone deficiency (<280 ng/dL) | 1.3 | 1.2 | 1.4 | <0.001 | 1.3 | 1.2 | 1.4 | <0.001 | 1.2 | 1.1 | 1.3 | 0.001 |
|  | Excessive Testosterone (>1100 ng/dL) | 0.6 | 0.4 | 1.0 | 0.039 | 0.7 | 0.5 | 1.0 | 0.075 | 0.7 | 0.4 | 1.0 | 0.054 |
|  | Estradiol deficiency (<10 pg/mL) | 1.3 | 1.0 | 1.7 | 0.033 | 1.2 | 0.9 | 1.5 | 0.261 | 1.2 | 0.9 | 1.5 | 0.245 |
|  | Excessive Estradiol (>50 pg/mL) | 0.8 | 0.6 | 1.0 | 0.046 | 0.8 | 0.6 | 1.1 | 0.110 | 0.8 | 0.6 | 1.0 | 0.035 |
|  | SHBG deficiency (<10 nmol/L) | 0.8 | 0.4 | 1.7 | 0.615 | 1.0 | 0.5 | 2.2 | 0.966 | 0.7 | 0.3 | 1.6 | 0.389 |
|  | Excessive SHBG (>57 nmol/L) | 1.0 | 0.9 | 1.1 | 0.863 | 0.9 | 0.8 | 1.0 | 0.003 | 0.9 | 0.8 | 1.0 | 0.006 |
| Log2-NLR | Testosterone deficiency (<280 ng/dL) | 1.3 | 1.2 | 1.4 | <0.001 | 1.2 | 1.1 | 1.4 | 0.001 | 1.2 | 1.0 | 1.3 | 0.020 |
|  | Excessive Testosterone (>1100 ng/dL) | 0.9 | 0.5 | 1.5 | 0.549 | 0.9 | 0.6 | 1.6 | 0.798 | 0.9 | 0.5 | 1.5 | 0.678 |
|  | Estradiol deficiency (<10 pg/mL) | 1.4 | 1.1 | 1.9 | 0.021 | 1.1 | 0.8 | 1.5 | 0.461 | 1.1 | 0.8 | 1.5 | 0.442 |
|  | Excessive Estradiol (>50 pg/mL) | 0.9 | 0.7 | 1.2 | 0.478 | 1.0 | 0.7 | 1.3 | 0.728 | 0.9 | 0.6 | 1.2 | 0.421 |
|  | SHBG deficiency (<10 nmol/L) | 0.7 | 0.3 | 1.4 | 0.260 | 0.9 | 0.3 | 2.2 | 0.761 | 0.6 | 0.2 | 1.6 | 0.307 |
|  | Excessive SHBG (>57 nmol/L) | 1.3 | 1.1 | 1.4 | <0.001 | 0.9 | 0.8 | 1.0 | 0.092 | 0.9 | 0.8 | 1.0 | 0.147 |
| Log2-PLR | Testosterone deficiency (<280 ng/dL) | 0.9 | 0.8 | 1.1 | 0.222 | 0.9 | 0.8 | 1.0 | 0.112 | 1.0 | 0.8 | 1.1 | 0.660 |
|  | Excessive Testosterone (>1100 ng/dL) | 0.8 | 0.4 | 1.6 | 0.550 | 0.8 | 0.4 | 1.4 | 0.428 | 0.7 | 0.4 | 1.2 | 0.175 |
|  | Estradiol deficiency (<10 pg/mL) | 1.2 | 0.8 | 1.8 | 0.334 | 1.1 | 0.8 | 1.6 | 0.580 | 1.1 | 0.7 | 1.5 | 0.805 |
|  | Excessive Estradiol (>50 pg/mL) | 0.7 | 0.5 | 0.9 | 0.009 | 0.6 | 0.5 | 0.9 | 0.005 | 0.7 | 0.5 | 0.9 | 0.013 |
|  | SHBG deficiency (<10 nmol/L) | 0.7 | 0.3 | 1.6 | 0.368 | 0.8 | 0.2 | 2.4 | 0.650 | 0.7 | 0.2 | 2.2 | 0.504 |
|  | Excessive SHBG (>57 nmol/L) | 1.4 | 1.2 | 1.6 | <0.001 | 1.2 | 1.0 | 1.3 | 0.028 | 1.0 | 0.9 | 1.2 | 0.598 |
| Log2-PPN | Testosterone deficiency (<280 ng/dL) | 1.5 | 1.3 | 1.6 | <0.001 | 1.5 | 1.4 | 1.7 | <0.001 | 1.4 | 1.2 | 1.5 | <0.001 |
|  | Excessive Testosterone (>1100 ng/dL) | 0.5 | 0.3 | 0.7 | 0.001 | 0.5 | 0.3 | 0.8 | 0.004 | 0.5 | 0.3 | 0.8 | 0.008 |
|  | Estradiol deficiency (<10 pg/mL) | 1.3 | 1.0 | 1.7 | 0.104 | 1.2 | 0.9 | 1.6 | 0.190 | 1.3 | 1.0 | 1.7 | 0.101 |
|  | Excessive Estradiol (>50 pg/mL) | 0.8 | 0.6 | 1.1 | 0.101 | 0.9 | 0.7 | 1.2 | 0.352 | 0.8 | 0.6 | 1.0 | 0.065 |
|  | SHBG deficiency (<10 nmol/L) | 1.3 | 0.6 | 2.7 | 0.549 | 1.3 | 0.6 | 2.9 | 0.512 | 0.9 | 0.4 | 1.9 | 0.692 |
|  | Excessive SHBG (>57 nmol/L) | 0.7 | 0.6 | 0.8 | <0.001 | 0.7 | 0.6 | 0.8 | <0.001 | 0.8 | 0.7 | 0.8 | <0.001 |
| Log2-LC | Testosterone deficiency (<280 ng/dL) | 1.2 | 1.1 | 1.4 | 0.008 | 1.4 | 1.2 | 1.6 | <0.001 | 1.2 | 1.0 | 1.4 | 0.014 |
|  | Excessive Testosterone (>1100 ng/dL) | 0.6 | 0.3 | 1.1 | 0.102 | 0.6 | 0.3 | 1.2 | 0.118 | 0.7 | 0.3 | 1.5 | 0.327 |
|  | Estradiol deficiency (<10 pg/mL) | 0.8 | 0.6 | 1.2 | 0.367 | 1.0 | 0.7 | 1.5 | 0.911 | 1.1 | 0.8 | 1.6 | 0.638 |
|  | Excessive Estradiol (>50 pg/mL) | 1.2 | 0.8 | 1.7 | 0.509 | 1.3 | 0.8 | 1.9 | 0.270 | 1.1 | 0.7 | 1.7 | 0.591 |
|  | SHBG deficiency (<10 nmol/L) | 2.0 | 1.0 | 4.2 | 0.065 | 2.0 | 0.5 | 7.3 | 0.303 | 1.6 | 0.4 | 6.0 | 0.460 |
|  | Excessive SHBG (>57 nmol/L) | 0.5 | 0.4 | 0.5 | <0.001 | 0.7 | 0.6 | 0.8 | <0.001 | 0.8 | 0.7 | 0.9 | 0.004 |
| Log2-NC | Testosterone deficiency (<280 ng/dL) | 1.8 | 1.6 | 2.0 | <0.001 | 1.8 | 1.6 | 2.1 | <0.001 | 1.5 | 1.3 | 1.8 | <0.001 |
|  | Excessive Testosterone (>1100 ng/dL) | 0.5 | 0.3 | 0.9 | 0.027 | 0.6 | 0.3 | 1.1 | 0.091 | 0.6 | 0.3 | 1.2 | 0.166 |
|  | Estradiol deficiency (<10 pg/mL) | 1.5 | 1.0 | 2.1 | 0.043 | 1.2 | 0.8 | 1.8 | 0.284 | 1.4 | 0.9 | 2.0 | 0.136 |
|  | Excessive Estradiol (>50 pg/mL) | 1.0 | 0.7 | 1.4 | 0.783 | 1.1 | 0.8 | 1.6 | 0.571 | 0.9 | 0.6 | 1.4 | 0.616 |
|  | SHBG deficiency (<10 nmol/L) | 1.1 | 0.4 | 2.9 | 0.894 | 1.3 | 0.5 | 3.5 | 0.651 | 0.8 | 0.3 | 2.2 | 0.591 |
|  | Excessive SHBG (>57 nmol/L) | 0.7 | 0.7 | 0.8 | <0.001 | 0.6 | 0.5 | 0.7 | <0.001 | 0.7 | 0.6 | 0.8 | <0.001 |
| Log2-PLT | Testosterone deficiency (<280 ng/dL) | 1.2 | 1.0 | 1.5 | 0.061 | 1.4 | 1.2 | 1.8 | 0.001 | 1.3 | 1.1 | 1.6 | 0.008 |
|  | Excessive Testosterone (>1100 ng/dL) | 0.3 | 0.2 | 0.6 | 0.001 | 0.3 | 0.1 | 0.6 | 0.001 | 0.3 | 0.1 | 0.6 | 0.001 |
|  | Estradiol deficiency (<10 pg/mL) | 1.1 | 0.6 | 1.8 | 0.858 | 1.3 | 0.8 | 2.3 | 0.315 | 1.3 | 0.8 | 2.3 | 0.298 |
|  | Excessive Estradiol (>50 pg/mL) | 0.5 | 0.3 | 0.8 | 0.004 | 0.5 | 0.3 | 0.8 | 0.006 | 0.5 | 0.3 | 0.8 | 0.004 |
|  | SHBG deficiency (<10 nmol/L) | 2.2 | 0.5 | 10.3 | 0.315 | 1.7 | 0.3 | 9.3 | 0.516 | 1.0 | 0.2 | 5.6 | 0.986 |
|  | Excessive SHBG (>57 nmol/L) | 0.5 | 0.4 | 0.6 | <0.001 | 0.7 | 0.6 | 0.9 | 0.002 | 0.7 | 0.6 | 0.9 | 0.002 |

Model 1: Unadjusted model. Model 2: Adjusts for age, race, education, marital status, family income to poverty ratio. Model 3: Adjusts for age, race, education, marital status, family income to poverty ratio, body mass index (kg/m^2^), smoking and drinking. TT, Testosterone; E_2_: Estradiol; SHBG: sex hormone-binding globulin; FAI: free androgen index; LC, lymphocyte count; NC, neutrophil count; NLR, neutrophil-to-lymphocyte ratio; PLT, platelet count; PLR, platelet-to-lymphocyte ratio; PPN, the product of platelet count and neutrophil count; SII, systemic immune-inflammation index. Bold fonts indicate P value < 0.05.

**Supplementary Table S12.** Association between [Inflammation](https://www.frontiersin.org/journals/immunology/sections/inflammation)-related Index and sex hormones disorder in Female postmenopausal group

| Index | Outcome | Model1 | | | | Model 2 | | | | Model3 | | | |
| --- | --- | --- | --- | --- | --- | --- | --- | --- | --- | --- | --- | --- | --- |
|  |  | OR | 95%CI low | 95%CI upp | p-value | OR | 95%CI low | 95%CI upp | p-value | OR | 95%CI low | 95%CI upp | p-value |
| Log2-SII | Testosterone deficiency (<15 ng/dL) | 1.2 | 1.0 | 1.3 | 0.008 | 1.2 | 1.0 | 1.3 | 0.006 | 1.2 | 1.1 | 1.3 | 0.002 |
|  | Excessive Testosterone (>70 ng/dL) | 0.7 | 0.6 | 0.9 | 0.013 | 0.8 | 0.6 | 1.0 | 0.037 | 0.8 | 0.6 | 1.0 | 0.043 |
|  | Excessive Estradiol (>20 pg/mL) | 1.2 | 1.0 | 1.4 | 0.028 | 1.3 | 1.1 | 1.6 | 0.012 | 1.2 | 1.0 | 1.5 | 0.077 |
|  | SHBG deficiency (<18 nmol/L) | 1.0 | 0.6 | 1.4 | 0.839 | 0.8 | 0.5 | 1.3 | 0.463 | 0.7 | 0.5 | 1.2 | 0.207 |
|  | Excessive SHBG (>144 nmol/L) | 0.8 | 0.6 | 1.0 | 0.014 | 0.7 | 0.6 | 0.9 | 0.003 | 0.8 | 0.6 | 0.9 | 0.011 |
| Log2-NLR | Testosterone deficiency (<15 ng/dL) | 1.2 | 1.1 | 1.4 | 0.003 | 1.2 | 1.1 | 1.4 | 0.005 | 1.2 | 1.1 | 1.4 | 0.002 |
|  | Excessive Testosterone (>70 ng/dL) | 0.8 | 0.6 | 1.1 | 0.107 | 0.8 | 0.6 | 1.1 | 0.145 | 0.8 | 0.6 | 1.1 | 0.155 |
|  | Excessive Estradiol (>20 pg/mL) | 1.2 | 1.0 | 1.4 | 0.122 | 1.5 | 1.2 | 1.9 | <0.001 | 1.4 | 1.1 | 1.8 | 0.003 |
|  | SHBG deficiency (<18 nmol/L) | 1.0 | 0.6 | 1.6 | 0.945 | 1.0 | 0.6 | 1.7 | 0.876 | 0.8 | 0.5 | 1.5 | 0.526 |
|  | Excessive SHBG (>144 nmol/L) | 1.0 | 0.8 | 1.2 | 0.868 | 0.8 | 0.7 | 1.1 | 0.165 | 0.9 | 0.7 | 1.1 | 0.258 |
| Log2-PLR | Testosterone deficiency (<15 ng/dL) | 1.2 | 1.0 | 1.4 | 0.034 | 1.2 | 1.0 | 1.4 | 0.019 | 1.2 | 1.0 | 1.4 | 0.033 |
|  | Excessive Testosterone (>70 ng/dL) | 0.7 | 0.5 | 1.0 | 0.043 | 0.7 | 0.5 | 1.0 | 0.043 | 0.7 | 0.5 | 1.0 | 0.039 |
|  | Excessive Estradiol (>20 pg/mL) | 1.1 | 0.8 | 1.4 | 0.594 | 1.1 | 0.8 | 1.5 | 0.467 | 1.1 | 0.8 | 1.5 | 0.485 |
|  | SHBG deficiency (<18 nmol/L) | 0.4 | 0.3 | 0.7 | <0.001 | 0.3 | 0.2 | 0.5 | <0.001 | 0.3 | 0.2 | 0.6 | <0.001 |
|  | Excessive SHBG (>144 nmol/L) | 1.1 | 0.8 | 1.4 | 0.739 | 1.0 | 0.7 | 1.3 | 0.888 | 0.9 | 0.6 | 1.2 | 0.323 |
| Log2-PPN | Testosterone deficiency (<15 ng/dL) | 1.1 | 1.0 | 1.2 | 0.195 | 1.1 | 1.0 | 1.2 | 0.141 | 1.1 | 1.0 | 1.3 | 0.036 |
|  | Excessive Testosterone (>70 ng/dL) | 0.7 | 0.6 | 1.0 | 0.026 | 0.8 | 0.6 | 1.1 | 0.106 | 0.8 | 0.6 | 1.1 | 0.135 |
|  | Excessive Estradiol (>20 pg/mL) | 1.3 | 1.1 | 1.5 | 0.005 | 1.2 | 1.0 | 1.4 | 0.138 | 1.1 | 0.9 | 1.3 | 0.631 |
|  | SHBG deficiency (<18 nmol/L) | 1.7 | 1.1 | 2.7 | 0.015 | 1.5 | 0.9 | 2.3 | 0.118 | 1.2 | 0.7 | 1.9 | 0.515 |
|  | Excessive SHBG (>144 nmol/L) | 0.6 | 0.5 | 0.7 | <0.001 | 0.6 | 0.5 | 0.7 | <0.001 | 0.7 | 0.5 | 0.8 | <0.001 |
| Log2-LC | Testosterone deficiency (<15 ng/dL) | 0.8 | 0.7 | 1.0 | 0.032 | 0.8 | 0.7 | 1.0 | 0.041 | 0.9 | 0.7 | 1.0 | 0.090 |
|  | Excessive Testosterone (>70 ng/dL) | 1.1 | 0.7 | 1.6 | 0.625 | 1.2 | 0.8 | 1.7 | 0.356 | 1.2 | 0.8 | 1.8 | 0.309 |
|  | Excessive Estradiol (>20 pg/mL) | 1.1 | 0.9 | 1.4 | 0.454 | 0.8 | 0.6 | 1.1 | 0.103 | 0.7 | 0.5 | 1.0 | 0.043 |
|  | SHBG deficiency (<18 nmol/L) | 2.5 | 1.6 | 4.1 | <0.001 | 3.0 | 1.7 | 5.3 | <0.001 | 2.6 | 1.4 | 4.7 | 0.002 |
|  | Excessive SHBG (>144 nmol/L) | 0.5 | 0.4 | 0.7 | <0.001 | 0.7 | 0.5 | 0.9 | 0.010 | 0.8 | 0.6 | 1.1 | 0.167 |
| Log2-NC | Testosterone deficiency (<15 ng/dL) | 1.1 | 1.0 | 1.3 | 0.094 | 1.1 | 1.0 | 1.3 | 0.117 | 1.2 | 1.0 | 1.4 | 0.019 |
|  | Excessive Testosterone (>70 ng/dL) | 0.8 | 0.5 | 1.1 | 0.131 | 0.8 | 0.6 | 1.2 | 0.331 | 0.8 | 0.6 | 1.3 | 0.394 |
|  | Excessive Estradiol (>20 pg/mL) | 1.4 | 1.1 | 1.7 | 0.010 | 1.5 | 1.1 | 1.9 | 0.007 | 1.3 | 1.0 | 1.7 | 0.075 |
|  | SHBG deficiency (<18 nmol/L) | 2.9 | 1.6 | 5.1 | <0.001 | 2.6 | 1.4 | 4.9 | 0.003 | 1.9 | 1.0 | 3.7 | 0.056 |
|  | Excessive SHBG (>144 nmol/L) | 0.6 | 0.4 | 0.8 | <0.001 | 0.5 | 0.4 | 0.7 | <0.001 | 0.6 | 0.5 | 0.9 | 0.005 |
| Log2-PLT | Testosterone deficiency (<15 ng/dL) | 1.0 | 0.8 | 1.3 | 0.898 | 1.1 | 0.9 | 1.4 | 0.502 | 1.1 | 0.9 | 1.4 | 0.408 |
|  | Excessive Testosterone (>70 ng/dL) | 0.6 | 0.4 | 0.9 | 0.023 | 0.6 | 0.4 | 1.0 | 0.068 | 0.6 | 0.4 | 1.1 | 0.083 |
|  | Excessive Estradiol (>20 pg/mL) | 1.4 | 1.0 | 2.0 | 0.069 | 0.8 | 0.5 | 1.2 | 0.261 | 0.7 | 0.5 | 1.1 | 0.095 |
|  | SHBG deficiency (<18 nmol/L) | 0.9 | 0.4 | 2.0 | 0.763 | 0.6 | 0.2 | 1.4 | 0.212 | 0.5 | 0.2 | 1.2 | 0.128 |
|  | Excessive SHBG (>144 nmol/L) | 0.4 | 0.3 | 0.6 | <0.001 | 0.5 | 0.3 | 0.7 | <0.001 | 0.5 | 0.3 | 0.7 | 0.001 |

Model 1: Unadjusted model. Model 2: Adjusts for age, race, education, marital status, family income to poverty ratio. Model 3: Adjusts for age, race, education, marital status, family income to poverty ratio, body mass index (kg/m^2^), smoking and drinking. TT, Testosterone; E_2_: Estradiol; SHBG: sex hormone-binding globulin; FAI: free androgen index; LC, lymphocyte count; NC, neutrophil count; NLR, neutrophil-to-lymphocyte ratio; PLT, platelet count; PLR, platelet-to-lymphocyte ratio; PPN, the product of platelet count and neutrophil count; SII, systemic immune-inflammation index. Bold fonts indicate P value < 0.05.

**Supplementary Table S13** Association between [Inflammation](https://www.frontiersin.org/journals/immunology/sections/inflammation)-related Index and sex hormones disorder in Female premenopausal group

| Index | Outcome | Model1 | | | | Model 2 | | | | Model3 | | | |
| --- | --- | --- | --- | --- | --- | --- | --- | --- | --- | --- | --- | --- | --- |
|  |  | OR | 95%CI low | 95%CI upp | p-value | OR | 95%CI low | 95%CI upp | p-value | OR | 95%CI low | 95%CI upp | p-value |
| Log2-SII | Testosterone deficiency (<15 ng/dL) | 1.0 | 0.9 | 1.2 | 0.758 | 1.0 | 0.9 | 1.2 | 0.672 | 1.1 | 0.9 | 1.2 | 0.437 |
|  | Excessive Testosterone (>70 ng/dL) | 0.9 | 0.6 | 1.5 | 0.753 | 0.9 | 0.6 | 1.5 | 0.789 | 0.9 | 0.6 | 1.4 | 0.604 |
|  | Estradiol deficiency (<20 pg/mL) | 0.8 | 0.7 | 0.9 | 0.004 | 0.7 | 0.6 | 0.9 | <0.001 | 0.8 | 0.7 | 0.9 | 0.002 |
|  | Excessive Estradiol (>350 pg/mL) | 1.4 | 1.0 | 2.0 | 0.060 | 1.5 | 1.1 | 2.1 | 0.018 | 1.6 | 1.2 | 2.3 | 0.006 |
|  | SHBG deficiency (<18 nmol/L) | 1.7 | 1.2 | 2.4 | 0.005 | 1.8 | 1.2 | 2.5 | 0.002 | 1.4 | 0.9 | 2.1 | 0.108 |
|  | Excessive SHBG (>144 nmol/L) | 0.9 | 0.7 | 1.1 | 0.188 | 0.9 | 0.7 | 1.1 | 0.196 | 1.0 | 0.8 | 1.2 | 0.754 |
| Log2-NLR | Testosterone deficiency (<15 ng/dL) | 1.0 | 0.9 | 1.2 | 0.949 | 1.0 | 0.8 | 1.2 | 0.805 | 1.0 | 0.9 | 1.2 | 0.967 |
|  | Excessive Testosterone (>70 ng/dL) | 0.9 | 0.5 | 1.6 | 0.818 | 1.0 | 0.6 | 1.7 | 0.945 | 0.9 | 0.5 | 1.6 | 0.729 |
|  | Estradiol deficiency (<20 pg/mL) | 0.8 | 0.7 | 1.0 | 0.019 | 0.7 | 0.6 | 0.8 | <0.001 | 0.7 | 0.6 | 0.9 | 0.001 |
|  | Excessive Estradiol (>350 pg/mL) | 1.9 | 1.2 | 2.8 | 0.003 | 2.1 | 1.4 | 3.2 | <0.001 | 2.2 | 1.4 | 3.2 | <0.001 |
|  | SHBG deficiency (<18 nmol/L) | 1.5 | 1.0 | 2.3 | 0.075 | 1.6 | 1.1 | 2.6 | 0.028 | 1.4 | 0.9 | 2.2 | 0.183 |
|  | Excessive SHBG (>144 nmol/L) | 1.0 | 0.8 | 1.2 | 0.723 | 0.9 | 0.7 | 1.2 | 0.598 | 1.0 | 0.8 | 1.3 | 0.998 |
| Log2-PLR | Testosterone deficiency (<15 ng/dL) | 1.1 | 0.9 | 1.4 | 0.213 | 1.0 | 0.8 | 1.2 | 0.805 | 1.0 | 0.8 | 1.2 | 0.844 |
|  | Excessive Testosterone (>70 ng/dL) | 0.8 | 0.4 | 1.7 | 0.595 | 0.9 | 0.4 | 1.7 | 0.639 | 0.9 | 0.5 | 1.7 | 0.739 |
|  | Estradiol deficiency (<20 pg/mL) | 1.0 | 0.8 | 1.2 | 0.610 | 0.8 | 0.6 | 1.0 | 0.015 | 0.8 | 0.6 | 1.0 | 0.018 |
|  | Excessive Estradiol (>350 pg/mL) | 1.1 | 0.6 | 1.8 | 0.795 | 1.1 | 0.7 | 1.8 | 0.741 | 1.1 | 0.7 | 1.8 | 0.692 |
|  | SHBG deficiency (<18 nmol/L) | 0.7 | 0.4 | 1.3 | 0.255 | 0.8 | 0.4 | 1.3 | 0.302 | 0.7 | 0.4 | 1.2 | 0.235 |
|  | Excessive SHBG (>144 nmol/L) | 1.0 | 0.8 | 1.3 | 0.834 | 1.0 | 0.8 | 1.4 | 0.856 | 1.0 | 0.8 | 1.3 | 0.956 |
| Log2-PPN | Testosterone deficiency (<15 ng/dL) | 1.0 | 0.9 | 1.1 | 0.864 | 1.1 | 0.9 | 1.2 | 0.400 | 1.1 | 1.0 | 1.3 | 0.174 |
|  | Excessive Testosterone (>70 ng/dL) | 1.0 | 0.6 | 1.5 | 0.943 | 1.0 | 0.6 | 1.5 | 0.880 | 0.9 | 0.6 | 1.4 | 0.611 |
|  | Estradiol deficiency (<20 pg/mL) | 0.8 | 0.7 | 0.9 | 0.001 | 0.8 | 0.7 | 0.9 | 0.004 | 0.9 | 0.8 | 1.0 | 0.086 |
|  | Excessive Estradiol (>350 pg/mL) | 1.2 | 0.9 | 1.7 | 0.286 | 1.3 | 0.9 | 1.8 | 0.140 | 1.5 | 1.0 | 2.1 | 0.035 |
|  | SHBG deficiency (<18 nmol/L) | 2.4 | 1.6 | 3.4 | <0.001 | 2.5 | 1.7 | 3.5 | <0.001 | 1.8 | 1.2 | 2.6 | 0.004 |
|  | Excessive SHBG (>144 nmol/L) | 0.8 | 0.7 | 1.0 | 0.036 | 0.8 | 0.7 | 1.0 | 0.031 | 0.9 | 0.8 | 1.1 | 0.511 |
| Log2-LC | Testosterone deficiency (<15 ng/dL) | 0.9 | 0.8 | 1.1 | 0.427 | 1.1 | 0.9 | 1.4 | 0.465 | 1.1 | 0.9 | 1.4 | 0.335 |
|  | Excessive Testosterone (>70 ng/dL) | 1.2 | 0.5 | 2.5 | 0.697 | 1.1 | 0.5 | 2.3 | 0.853 | 1.0 | 0.5 | 2.2 | 0.996 |
|  | Estradiol deficiency (<20 pg/mL) | 0.9 | 0.7 | 1.1 | 0.314 | 1.2 | 1.0 | 1.6 | 0.104 | 1.3 | 1.0 | 1.7 | 0.028 |
|  | Excessive Estradiol (>350 pg/mL) | 0.7 | 0.4 | 1.2 | 0.216 | 0.7 | 0.4 | 1.2 | 0.185 | 0.7 | 0.4 | 1.3 | 0.305 |
|  | SHBG deficiency (<18 nmol/L) | 2.9 | 1.6 | 5.4 | 0.001 | 2.8 | 1.5 | 5.2 | 0.001 | 2.0 | 1.1 | 3.8 | 0.034 |
|  | Excessive SHBG (>144 nmol/L) | 0.8 | 0.6 | 1.1 | 0.153 | 0.8 | 0.6 | 1.1 | 0.110 | 0.9 | 0.7 | 1.3 | 0.563 |
| Log2-NC | Testosterone deficiency (<15 ng/dL) | 1.0 | 0.8 | 1.1 | 0.499 | 1.0 | 0.9 | 1.2 | 0.747 | 1.1 | 0.9 | 1.3 | 0.405 |
|  | Excessive Testosterone (>70 ng/dL) | 1.0 | 0.6 | 1.8 | 0.952 | 1.0 | 0.6 | 1.9 | 0.942 | 0.9 | 0.5 | 1.7 | 0.711 |
|  | Estradiol deficiency (<20 pg/mL) | 0.8 | 0.6 | 0.9 | 0.001 | 0.8 | 0.6 | 0.9 | 0.003 | 0.8 | 0.7 | 1.0 | 0.058 |
|  | Excessive Estradiol (>350 pg/mL) | 1.6 | 1.0 | 2.5 | 0.033 | 1.9 | 1.2 | 3.0 | 0.007 | 2.2 | 1.4 | 3.5 | 0.001 |
|  | SHBG deficiency (<18 nmol/L) | 2.9 | 1.8 | 4.7 | <0.001 | 3.3 | 2.0 | 5.3 | <0.001 | 2.4 | 1.4 | 4.0 | 0.001 |
|  | Excessive SHBG (>144 nmol/L) | 0.8 | 0.7 | 1.1 | 0.142 | 0.8 | 0.6 | 1.0 | 0.075 | 0.9 | 0.7 | 1.2 | 0.653 |
| Log2-PLT | Testosterone deficiency (<15 ng/dL) | 1.1 | 0.9 | 1.4 | 0.461 | 1.2 | 0.9 | 1.6 | 0.211 | 1.2 | 0.9 | 1.6 | 0.137 |
|  | Excessive Testosterone (>70 ng/dL) | 0.9 | 0.3 | 2.3 | 0.803 | 0.8 | 0.3 | 2.1 | 0.666 | 0.8 | 0.3 | 2.0 | 0.637 |
|  | Estradiol deficiency (<20 pg/mL) | 0.8 | 0.6 | 1.0 | 0.049 | 0.8 | 0.6 | 1.1 | 0.186 | 0.9 | 0.7 | 1.3 | 0.568 |
|  | Excessive Estradiol (>350 pg/mL) | 0.7 | 0.3 | 1.3 | 0.233 | 0.7 | 0.4 | 1.3 | 0.241 | 0.8 | 0.4 | 1.6 | 0.479 |
|  | SHBG deficiency (<18 nmol/L) | 2.9 | 1.3 | 6.3 | 0.007 | 2.8 | 1.3 | 6.0 | 0.010 | 1.5 | 0.7 | 3.3 | 0.330 |
|  | Excessive SHBG (>144 nmol/L) | 0.7 | 0.5 | 1.0 | 0.038 | 0.7 | 0.5 | 1.0 | 0.079 | 0.9 | 0.6 | 1.3 | 0.514 |

Model 1: Unadjusted model. Model 2: Adjusts for age, race, education, marital status, family income to poverty ratio. Model 3: Adjusts for age, race, education, marital status, family income to poverty ratio, body mass index (kg/m^2^), smoking and drinking. TT, Testosterone; E_2_: Estradiol; SHBG: sex hormone-binding globulin; FAI: free androgen index; LC, lymphocyte count; NC, neutrophil count; NLR, neutrophil-to-lymphocyte ratio; PLT, platelet count; PLR, platelet-to-lymphocyte ratio; PPN, the product of platelet count and neutrophil count; SII, systemic immune-inflammation index. Bold fonts indicate P value < 0.05.
